# Elevated recombinant *clyA* gene expression in the uropathogenic *Escherichia coli* strain 536*,* a clue to explain pathoadaptive mutations in a subset of extraintestinal *E. coli* strains

**DOI:** 10.1186/s12866-014-0216-4

**Published:** 2014-09-02

**Authors:** Constance Oben Ayuk Enow, Jan Oscarsson, Nikola Zlatkov, Marie Westermark, Marylise Duperthuy, Sun Nyunt Wai, Bernt Eric Uhlin

**Affiliations:** Department of Molecular Biology, the Laboratory for Molecular Infection Medicine Sweden (MIMS), Umeå University, S-90187 Umeå, Sweden; Present address: Department of Odontology, Oral Microbiology, Umeå University, S-90187 Umeå, Sweden

**Keywords:** ClyA cytolysin, Pathoadaptive mutations, *clyA* gene expression, Extraintestinal *Escherichia coli*, SfaX regulatory protein

## Abstract

**Background:**

Analysis of the *Escherichia coli* collection of reference strains (ECOR) for the presence of the gene locus *clyA*, which encodes the pore-forming protein ClyA (cytolysin A), revealed that a non-functional *clyA* locus is common among certain extraintestinal pathogenic *E. coli* (ExPEC). In fact, all 15 ECOR group B2 strains and several additionally examined extraintestinal pathogenic (uropathogenic (UPEC) and neonatal meningitis (NBM)) *E. coli* strains contained various Δ*clyA* alleles.

**Results:**

There are at least four different variants of Δ*clyA*, suggesting that such deletions in *clyA* have arisen at more than one occasion. On the basis of this occurrence of the truncated *clyA* genes, we considered that there may be a patho-adaptive selection for deletions in *clyA* in extraintestinal pathogenic *E. coli*. In *E. coli* K-12 the *clyA* gene has been viewed as “cryptic” since it is tightly silenced by the nucleoid structuring protein H-NS. We constructed a restored *clyA*^+^ locus in derivatives of the UPEC strain 536 for further investigation of this hypothesis and, in particular, how the gene would be expressed. Our results show that the level of *clyA*^+^ expression is highly increased in the UPEC derivatives in comparison with the non-pathogenic *E. coli* K-12. Transcription of the *clyA*^+^ gene was induced to even higher levels when the SfaX regulatory protein was overproduced. The derivative with a restored *clyA*^*+*^ locus displayed a somewhat slower growth than the parental UPEC strain 536 when a sub-inhibitory concentration of the antimicrobial peptide Polymyxin B was added to the growth medium.

**Conclusions:**

Taken together, our findings show that the *clyA*^+^ locus is expressed at an elevated level in the UPEC strain and we conclude that this is at least in part due to the effect of the SfaX/PapX transcriptional regulators.

**Electronic supplementary material:**

The online version of this article (doi:10.1186/s12866-014-0216-4) contains supplementary material, which is available to authorized users.

## Background

A majority of *Escherichia coli* strains are benign residents of the intestinal tract of mammals, however a minority of *E. coli* isolates are pathogenic and cause a variety of diseases ranging from diarrhea to urinary tract infections and to meningitis. Genes encoding virulence factors such as adhesins, invasins, and toxins that allow pathogenic *E. coli* to colonize, invade, and damage host cells, are often coordinately regulated and tend to be clustered in the genome.

The ability to lyse erythrocytes (hemolysis) by expression of hemolysins is a common feature among *E. coli* strains causing extraintestinal infections. One of the most characterized hemolysins is HlyA or α-hemolysin which is produced by uropathogenic *E. coli* (UPEC), although several other types of hemolysins have been described for *E. coli* from different patho-groups [1]. The *clyA* gene, located at 26.5 min on the *E. coli* chromosome, encodes a 34-kDa protein, ClyA (also referred to as HlyE and SheA) which causes lysis of mammalian cells by pore formation in a calcium-independent fashion. ClyA is the only cytolytic factor found in non-pathogenic strains of *E. coli* including the K-12 strains commonly used in laboratory studies [2-7]. The *clyA*^+^ transcription is known to be subjected to transcriptional silencing by the H-NS nucleoid protein in *E. coli* K-12 [8] and it can be activated by the transcriptional regulator SlyA [2,4,5,8]. Considering the strict regulation of *clyA* in non-pathogenic *E. coli* laboratory strains it is of interest to understand how this gene locus functions in other *E. coli* isolates. Sequences homologous to the *clyA* gene have been identified in a number of pathogenic isolates of *E. coli* [2,6,9,10]. In addition, upon screening of several different *Salmonella enterica* serovars, functional homologues to the *clyA* gene were identified in the typhoid *Salmonella* serovars Typhi and Paratyphi A [9]. The presence of the *clyA* gene in wild-type isolates of *Salmonella* suggests a conserved function of the gene product although its role in pathogenesis is unclear. Nevertheless, ClyA appears to be associated with virulence in *S. enterica*. The role of ClyA in *Salmonella* virulence was analyzed using the *S. enterica* serovars Brandenburg, Indiana, Panama, and Schwarzengrund; 21 different serotypes of the strains were examined and the presence of ClyA was suggested to be associated with virulence in these *S. enterica* serovars [11].

In bacteria, the process of adapting to a host may involve not only acquisition of virulence determinants but also loss of gene functions. Pathogenicity-adaptive, or patho-adaptive, mutations may represent a genetic means for enhancing bacterial virulence without horizontal transfer of specific virulence factors, i. e. genes that are detrimental to a pathogenic lifestyle are deleted [12]. Such patho-adaptive mutations, which occur following the acquisition of new genes, may represent fine-tuning of the genome repertoire of a newly created pathogen to adapt to its new pathogenic lifestyle [13]. Earlier studies have provided genetic and/or phenotypic evidence for naturally occurring mutations that are either required for, or dramatically increase the ability of bacteria to enter, spread within, or sustain themselves in a virulence niche [14,15]. For instance the spontaneous deletion of *cadA*, encoding lysine decarboxylase, substantially increases the virulence of entero-invasive *E. coli* (EIEC) and *Shigella spp.* [13].

*E. coli* is considered to be clonal, and phylogenetic analyses of this species have shown that the strains fall into four main groups: A, B1, B2 and D [16]. Recent attempts to establish a link between phylogeny and virulence suggest that the A and B1 phylogenetic groups should be considered to represent the normal flora of different vertebrates, and most human commensal strains originate from these groups [17-19]. The standard *Escherichia coli* collection of reference strains (ECOR), a set of *E. coli* strains isolated from diverse hosts and geographic locations, was designed to represent genotypic variation in *E. coli* [20]. The collection contains 72 wild-type *E. coli* isolates from human and 16 other mammalian species, obtained from a larger collection of approximately 2600 isolates [21]. Our previous results clearly showed that the *clyA* locus was truncated due to deletion mutations in several of the *E. coli* isolates [22]. One or more deletions in the *clyA* locus were found in 15 of 15 tested ECOR B2 strains [22]. Furthermore, deletion mutations in the *clyA* locus were identified in two of the 25 (8%) ECOR A strains, one of the 12 (8%) ECOR D strains and one of the four (25%) ECOR E strains. In contrast, an intact *clyA* locus was found in 100% of the 16 ECOR B1 isolates examined. The B2 phylogenetic group represents *E. coli* strains involved in extra-intestinal infections [19]. The uropathogenic *E. coli* isolates 536, J96, and five additional (strains AD110, DS-17, IA-2, IH11128 and IHE3034; see Table 1) previously described extra-intestinal pathogenic (uropathogenic *E. coli* and newborn meningitis *E. coli* (NMEC)) carried a truncated *clyA* gene. At least four different variants of Δ*clyA* exist among such strains suggesting that the deletions in *clyA* arose on more than one occasion [22]. Similar findings have been reported from additional surveys of *E. coli* isolates from different sources [23-25]. Furthermore, the recent study by Murase and co-workers showed that gene inactivation at the *clyA* locus also has occurred in strains of the ECOR B1 phylogroup [23-25].

**Table 1:** **Bacterial strains used in this work**

**Strain**	**Genotype/relevant characteristics, serotype**	**Reference/source**
ECOR collection	*E. coli* reference collection	[20]
536	Clinical UTI isolate, O6:K15:H31	[26]
J96	Clinical UTI isolate, O4:K6	[27]
JON47	J96 *clyA* ^+^ (Km^r^)	This work
JON53	536 *clyA* ^+^ (Km^r^)	This work
COE2	JON53 *clyA-luxAB*	This work
COE3	COE3/pAES1 (*sfaX* ^*+*^ clone)	This work
COE4	COE2/pBR322	This work
COE6	COE2/pHMG94 (*papI* ^*+*^ clone)	This work
AD110	Clinical UTI isolate, O6:K2:H1:F7	[28]
DS-17	Clinical UTI isolate, O6:K5	[29]
IA-2	Clinical UTI isolate, O6:H¯	[30]
IH11128	Clinical UTI isolate, O75:K5:H¯	[31]
IHE3034	Clinical NBM isolate, O18:K1	[32]
AES1	*sfaX::kan* (Km^r^) mutant of IHE3034	[33]
AES153	∆ *sfaY* mutant of IHE3034	This laboratory
DH5α	*endA1 hsdR17* (r_k_¯m_k_^+^) *supE44 thi-1 recA1 gyrA relA1 Δ(lacZYA-argF)*	[34]
MG1655	*E. coli* K-12 wildtype	[35]
MC1061	*araD*139 *Δ(ara leu)7697 ΔlacX74galU galK hsr hsm* ^*+*^ *strA*	[36]
M182	*Δ*(*lacIPOZY*) X74 *galK galU strA*	[36]
MC4100	*araD139Δ*(*lac*)U169 *strA thi*	[37]
BSN26	MC4100 *trp::tet* (Tc^r^)	[38]
JON33	BSN26 *clyA::luxAB*	[8]
MWK11	MC4100 *clyA* ^++^	[8]
MWK7	M182 Δ*clyA*	This laboratory

In this report we present findings with derivatives of *E. coli* K-12 and the UPEC isolate *E. coli* 536 in which we constructed a restored *clyA*^+^ locus as well as a *clyA-lux* chimeric operon to quantitatively measure expression at the transcriptional level under different growth conditions.

## Results

### Deletion mutations at the *clyA* locus of *Escherichia coli*

**Figure 1:**
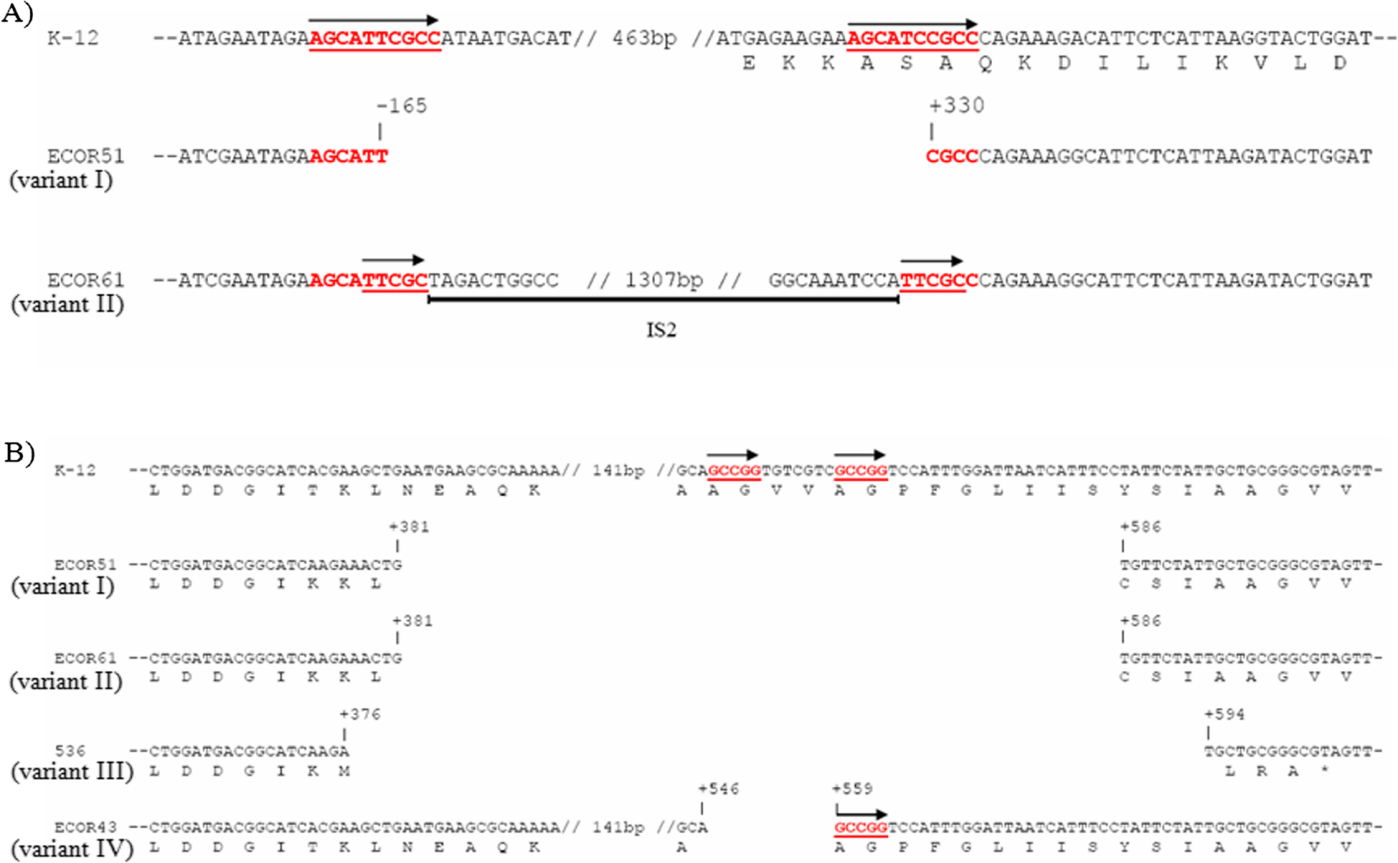
**Sequence representation of the different** ***clyA*** **deletion variants (indicated by roman numerals I-IV) found among different** ***E. coli*** **isolates with mutant** ***clyA*** **loci: ECOR51 (Δ** ***clyA*** **I), ECOR61 (Δ** ***clyA*** **II), 536 (Δ** ***clyA*** **III) and ECOR43 (Δ** ***clyA*** **IV).** Position coordinates are relative to the *clyA* start codon in *E. coli* K-12. Sequence repeats are indicated by red underlined bold face and are highlighted with arrows. The extent of the IS2 element is indicated by a black bar. **(A)** Deletions in the *clyA* upstream region and N-terminal coding sequences. **(B)** Deletions in the *clyA* C-terminal coding sequences.

Analysis of the DNA sequences revealed four different variants (denoted I to IV) of the Δ*clyA* alleles and differences in their distribution in the *E. coli* isolates [22-25]. The strains used in this study are summarized in Table 2 and sequence details are shown in Figure 1. Deletion variant I include two deletions: one major 493-bp deletion spanning from 164 bp upstream to 329 bp downstream of the *clyA* translational start codon, and one minor 204-bp deletion spanning from 382 to 585 bp downstream of the *clyA* translational start codon. Variant I was found in 13 of the 15 ECOR B2 strains, in ECOR23 and ECOR24 of group A, and in the UPEC/NBM isolates AD110, DS17, IA2, IH11128, IHE3034 and J96. Deletion variant II is similar to variant I, but with an intact 1327-bp IS-2 element positioned adjacent to the upstream 493-bp deletion in the opposite orientation of the *clyA* coding sequence. Variant II was found in ECOR61 and ECOR62 of group B2. Deletion variant III is similar to variant I with a deletion in the promoter region but in addition had a deletion in the *clyA* coding region. Variant III was identified in the UTI isolate 536. Deletion variant IV is a 12-bp-long in-frame deletion from 547 to 558 bp downstream of the *clyA* translational start. Variant IV was found in the ECOR43 and ECOR44 group E and D, respectively.

**Table 2:** **Distribution of truncated and intact** ***clyA*** **loci in the strains of the ECOR collection and additional extraintestinal** ***E. coli*** **isolates**

**Bacterial strain**	**Group** ^**1)**^	**Serotype**	***clyA*** **locus present** ^**2)**^	**Deletion variant** ^**3)**^
ECOR1-22, 25	A		+	
ECOR23-24	A		Δ	I
ECOR26-30, 32–34, 45, 58, 67-72	B1		+	
ECOR51-57, 59–60, 63-66	B2		Δ	I
ECOR61-62	B2		Δ	II
ECOR35-36, 38–41, 46-50	D		+	
ECOR44	D		Δ	IV
ECOR31, 37, 42	E		+	
ECOR43	E		Δ	IV
J96	UTI	O4:K6	Δ	I
536	UTI	O6:K15:H31	Δ	III
IH11128	UTI	O75:K5:H¯	Δ	I
AD110	UTI	O6:K2:H1:F7	Δ	I
DS-17	UTI	O6:K5	Δ	I
IA-2	UTI	O6:H¯	Δ	I
IHE3034	NBM	O18:K1:H7	Δ	I

^1)^The ECOR subgroups are as defined previously [16].

UTI, urinary tract infection. NBM, newborn meningitis.

^2)^ + denotes that an intact *clyA* locus is present; Δ denotes that some deletion(s) had ocurred.

^3)^I-IV denotes the different deletion variants within the *clyA* locus as described in the text and in Figure 1.

**Figure 2:**
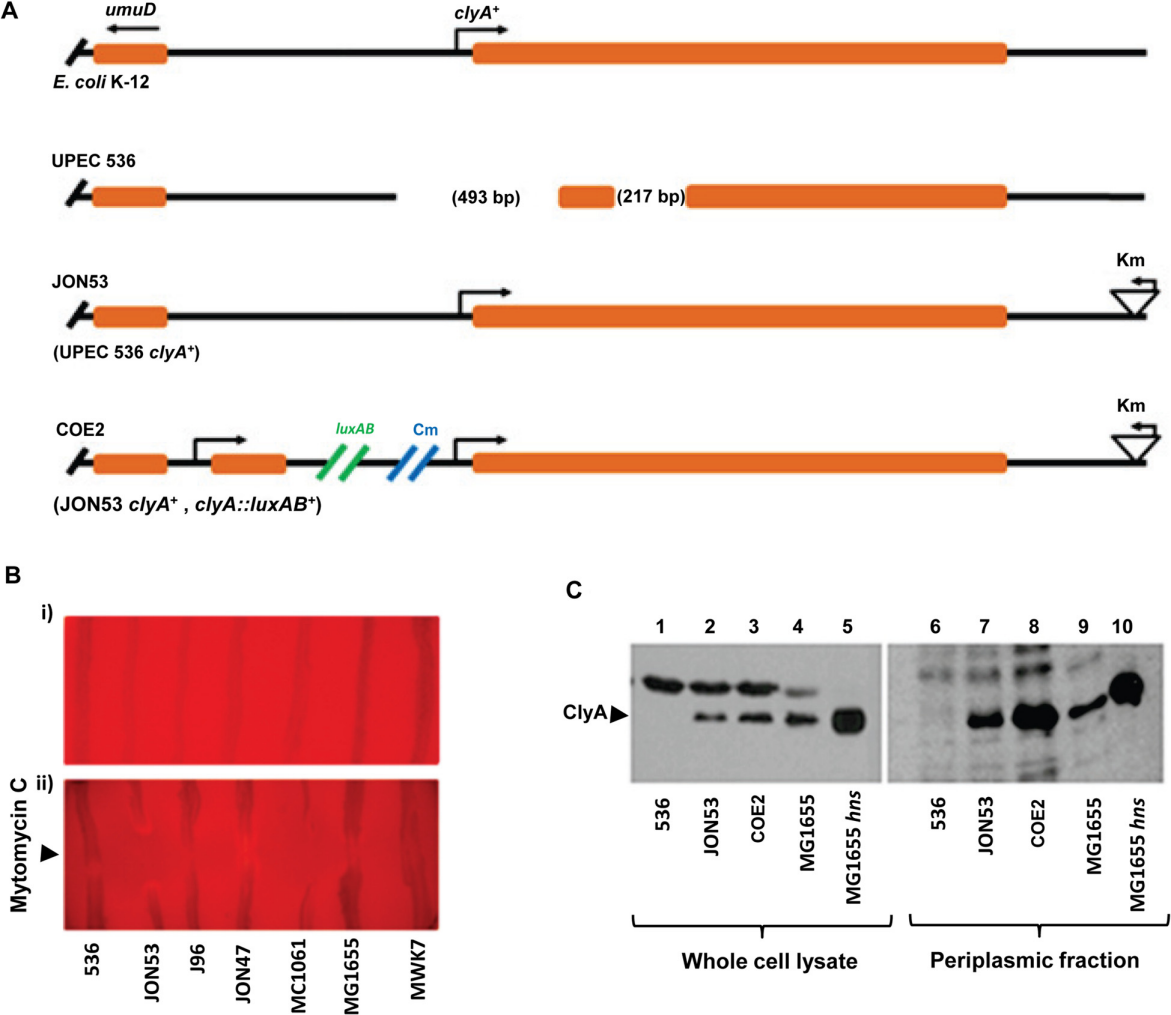
**Introduction of a** ***clyA*** ^**+**^ **locus in UPEC strains. (A) Schematic representation of the** ***clyA*** **loci in** ***E. coli*** **K-12 and in derivatives of UPEC strain 536.** In strain JON53 the *clyA* locus was restored in UPEC isolate 536 as described in Materials and Methods and a kanamycin resistance gene was inserted downstream of *clyA*. The strain COE2 is a derivative of JON53 with a chimeric *clyA::luxAB* operon included adjacent to the restored *clyA* ^*+*^ locus. **(B)** Hemolysis activity tests with *E. coli* K-12 and UPEC derivatives on blood plates under Ca^2+^ depleted conditions due to addition of Na-oxalate (final concentration 2 mM). Strains: 536 (UPEC, Δ*clyA*), JON53 (UPEC, *clyA* ^+^), J96 (UPEC, Δ*clyA*), JON47 (UPEC, *clyA* ^*+*^) MC1061 (K-12; *clyA* ^+^), MG1655 ( K-12; *clyA* ^*+*^), MWK7 (K-12; Δ*clyA*). Tests were performed in absence (i) and in presence (ii) of Mitomycin C applied onto the center of horizontally streaked rows of the *E. coli* strains. The images show the center part of the blood agar plates. **(C)** Detection of ClyA proteins by Western immunoblotting using a polyclonal ClyA antiserum. The immunoreactive bands corresponding to ClyA are indicated with an arrow. Whole cell lysates (lanes 1–5) and periplasmic fractions (lanes 6–10; prepared using osmolysis as described in Materials and Methods) were obtained from samples collected at OD_600_ = 2.0. Lanes 1 & 6: UPEC strain 536. Lanes 2 & 7: strain JON53. Lanes 3 & 8: strain COE2. Lanes 4 & 9: strain MG1655. Lanes 5 & 10: strain MG1655 *hns*.

The presence of a nearly perfect 10-bp repeat (AGCATTCGCC) immediately upstream of the major 493-bp deletion, and overlapping with the 3′ end of the deleted segment (AGCATCCGCC) (Figure 1A), suggested that the variant I deletion was the result of a recombination event. We found no such DNA repeats that would explain the internal 204-bp or 217-bp deletions in the *clyA* coding sequence (Figure 1B). As shown in the case of variant IV (Figure 1B) a short 5-bp repeat (GCCGG) was identified at the junctions of the 12-bp internal deletion observed in ECOR43 and ECOR44, indicating that this deletion is likely to be the result of a recombination event. The site of insertion of the IS2 element and the generated 5-bp direct repeats (TTCGC) in ECOR61 and ECOR62 suggested that the IS2 element was inserted after the deletion was generated (Figure 2A).

### Construction of a restored *clyA*^+^ locus in the UPEC strain *E. coli* 536

To study whether or not the absence of the *clyA* gene in extra-intestinal *E. coli* isolates reflects that this gene may be disadvantageous for the bacterial cells, *clyA*^+^ derivatives of the UPEC strains 536 and J96 were constructed. We used a suicide plasmid derivative (pJON176) and allelic exchange to introduce the *clyA* wild type allele, together with a kanamycin resistance cassette as a selectable marker at 350 bp downstream of the *clyA* stop codon, resulting in the strains JON47 (J96 *clyA*^*+*^) and JON53 (536 *clyA*^*+*^), respectively. A schematic illustration of the construct JON53 with restored *clyA*^+^ locus is shown in Figure 2A. The strains harboring the *clyA*^+^ allele at the correct position on the chromosome was confirmed by PCR analysis and DNA sequencing (data not shown). The restored *clyA* wild type allele in the UPEC strains was constructed with DNA sequences from *E. coli* K-12 and it was evident that these sequences were highly conserved among different *E. coli*. We performed a multiple sequence alignment of the *clyA* promoter region and coding sequences using a large set of publicly available *E. coli* genome sequences (NCBI Reference Sequence: NC_000913.3). It included sequences from different *E. coli* pathotypes and from commensally occurring isolates (see Additional file 1). Inspection of the upstream region revealed that sequences corresponding to the transcriptional and translational start sequences with the regulatory sequence elements (−35, −10, and Shine-Dalgarno sequences) and binding sites for regulatory proteins (CRP/FNR, SlyA), as defined from studies of *E. coli* K-12 derivatives, are conserved among the many different ExPEC and non-ExPEC *E. coli* isolates with intact *clyA* loci.

To assess the level of expression and activity of ClyA in JON47 and JON53, the hemolytic activity of the strains was scored on the blood agar plates. We observed a calcium-dependent hemolytic phenotype in strains JON47 and JON53 when they were grown on blood agar plates. When the Ca^2+^ chelator Na-oxalate was added (final concentration 2 mM), all strains showed a non-hemolytic phenotype (Figure 2B, panel i). In our earlier experiments, we showed that lysis of the host bacterial strains promote the release of ClyA using lytic bacteriophages or mitomycin C [9]. To deliberately provoke lysis of the bacterial cells, we placed 2 μl of mitomycin C (from a 1 mg ml^−1^ stock solution) onto horizontally streaked rows of the strains 536 and J96, and the *clyA*^+^ derivatives JON47 and JON53 on the blood agar plates (see Methods). Zones of hemolysis appeared for both JON53 (536 *clyA*^+^) and JON47 (J96 *clyA*^+^), but not for the parental strains 536 and J96 (Figure 2B, panel ii). The results clearly indicated that the restored *clyA* locus in these derivatives (JON53 and JON47) of strains 536 and J96 was expressed and our further tests verified that they could produce the ClyA protein at a detectable level (Figure 2C, lane 2 for strain JON53 and data not shown for strain JON47). The presence of ClyA protein in the *clyA*^+^ derivatives was confirmed by Western immunoblotting analysis using polyclonal anti-ClyA antiserum (Figure 2C). Subcellular localization experiments showed that ClyA protein was present in the periplasmic fraction of UPEC derivatives to the same extent as has been found in the case of *E. coli* K-12 (Figure 2C, lane 7 for strain JON53 and data not shown for strain JON47).

**Figure 3:**
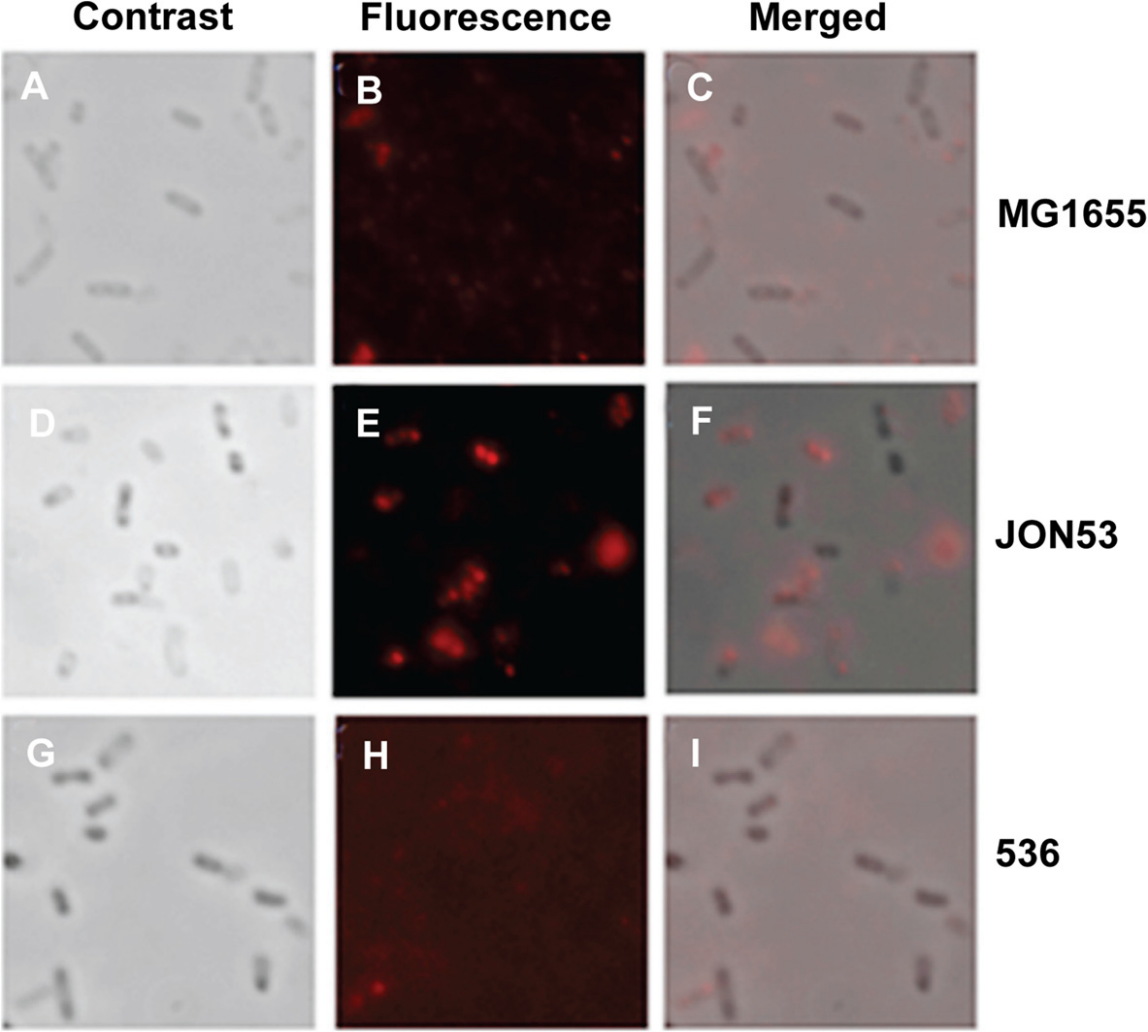
**Immunofluorescence microscopy detection of ClyA on bacterial cells.** Analyses were done with the *E. coli* K-12 strain MG1655 **(A, B C)**, the *clyA* ^*+*^ UPEC derivative JON53 **(D, E, F)** and the parental UPEC strain 536 **(G, H, I)**. Panels **A**, **D**, **G** show images obtained by by phase contrast microscopy. Panels **B**, **E**, **H** show images obtained from immunofluorescence analysis using polyclonal ClyA antiserum and AlexaFluor ^555^ –conjugated secondary antibody to enable visualization of ClyA as a red fluorescence signal. Panels **C**, **F**, **I** show the merged images.

We showed in earlier studies that in *clyA*^+^*E. coli* K-12 derivatives, a small subpopulation of bacterial cells (ca 1-2%) apparently expressed a high enough level of surface-exposed ClyA to be visible by immunofluorescence microscopy [39]. In order to compare the surface expression and export of ClyA protein in individual bacterial cells of the *clyA*^+^ UPEC derivative JON53 with the standard *E. coli* K-12 strain MG1655, we performed immunofluorescence microscopy with anti-ClyA antibodies and with the UPEC strain 536 used as negative control. As shown in Figure 3, ClyA was detected on the surface of several JON53 cells whereas there was very little immunofluorescence detected in case of the *E. coli* K-12 strain MG1655. Taken together, the studies of ClyA expression indicated that protein might be present at a somewhat higher level and/or exposed more abundantly on the surface of the JON53 bacterial cells in comparison with that of the *E coli* K-12 cells.

### Growth phase dependent expression of *clyA* in JON53

**Figure 4:**
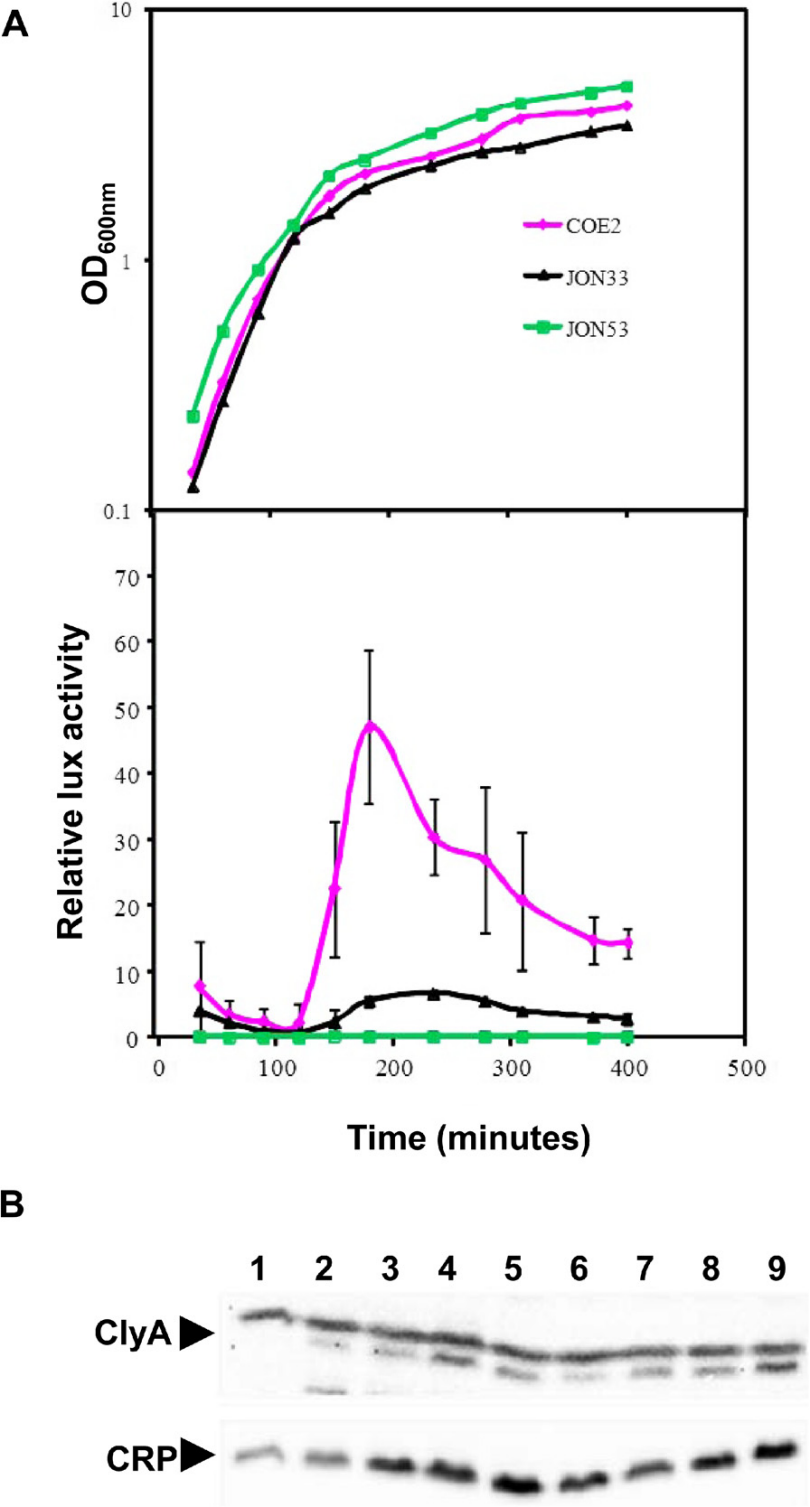
**Growth phase dependent** ***clyA*** **transcription.** Bacteria were grown in LB medium **(A)** at 37°C and samples were assayed for luciferase activity as described in Materials and Methods. The strains tested were: the UPEC derivative COE2 (*clyA::luxAB*), the *E. coli* K-12 strain BSN26 (*clyA::luxAB*) and the UPEC strain 536 *clyA* ^*+*^ (JON53) which was serving as a luciferase negative control. The growth curves (upper panel) are presented from a representative experiment. The data curves for luciferase activity (lower panel) represent the average value of three independent experiments and vertical bars indicate the standard deviations. **(B)** Immunoblot detection of ClyA and CRP proteins. The immunoreactive bands corresponding to ClyA and CRP are indicated with arrows. Bacteria were grown in LB medium at 37°C. Whole cell lysates were prepared from samples collected during different growth phases from the logarithmic phase of growth until the stationary phase. Lane 1: UPEC strain 536. Lanes 2–5: strain JON53, OD_600_ = 1.6 (lane2), OD_600_ = 1.74 (lane 3), OD_600_ = 2.24 (lane 4) and OD_600_ = 2.7 (lane 5). Lanes 6–9: COE2, OD_600_ = 1.5 (lane 6), OD_600_ = 1.8 (lane 7), OD_600_ = 2.04 (lane 8) and OD_600_ = 2.5 (lane 9).

In order to quantitatively monitor the transcriptional expression level of *clyA*, a *clyA::luxAB* operon construct was integrated in tandem to the *clyA*^+^ locus in the chromosome of JON53 as described in the Methods and schematically depicted in Figure 2A. The resulting strain was designated COE2 (JON53 *clyA::luxAB*). In our earlier studies, we observed the growth phase dependent expression of the *clyA* gene in the *E. coli* K-12 derivative carrying the *clyA::luxAB* construct (strain JON33) [8]. The transcription level of the *clyA::luxAB* operon in strain COE2 was therefore compared to the level in strain JON33 throughout the growth cycle. As shown in Figure 4A, the luciferase activity of the UPEC derivative COE2 increased continuously during the exponential growth phase and peaked at the late logarithmic phase of growth where it showed a more than five-fold increase compared to the *E. coli* K-12 *clyA::luxAB* operon derivative JON33.

### Transcription of *clyA* can be activated by the SfaX/PapX transcriptional regulatory protein family encoded by fimbrial gene clusters in UPEC strains

In *E. coli* K-12 the transcriptional regulator SlyA is known to activate *clyA*^+^ transcription by counteracting the silencing the H-NS nucleoid protein is causing [2,4,5,8]. However, the *slyA* gene seems to be present in most, if not all, *E. coli* and the same is true for the *hns* gene, suggesting that their role in *clyA* transcriptional regulation (anti-silencing vs. silencing) would be conserved among different non-pathogenic and pathogenic *E. coli*. Nevertheless, we performed a multiple sequence alignment of the *slyA* promoter region and coding sequences using a large set of publicly available *E. coli* genome sequences (NCBI Reference Sequence: NC_000913.3) to see if there would be any differences hinting to altered regulation of *slyA* expression among different *E. coli*. It included sequences from different *E. coli* pathotypes (ExPEC and non-ExPEC) and from commensally occurring isolates. Inspection of the upstream region revealed that sequences corresponding to the transcriptional and translational start sequences with the regulatory sequence elements (−35, −10, and Shine-Dalgarno sequences), as defined from studies of *E. coli* K-12 derivatives, are completely conserved among the many different *E. coli* isolates indicating that there would be no obvious difference in *slyA* expression and regulation (data not shown).

**Figure 5:**
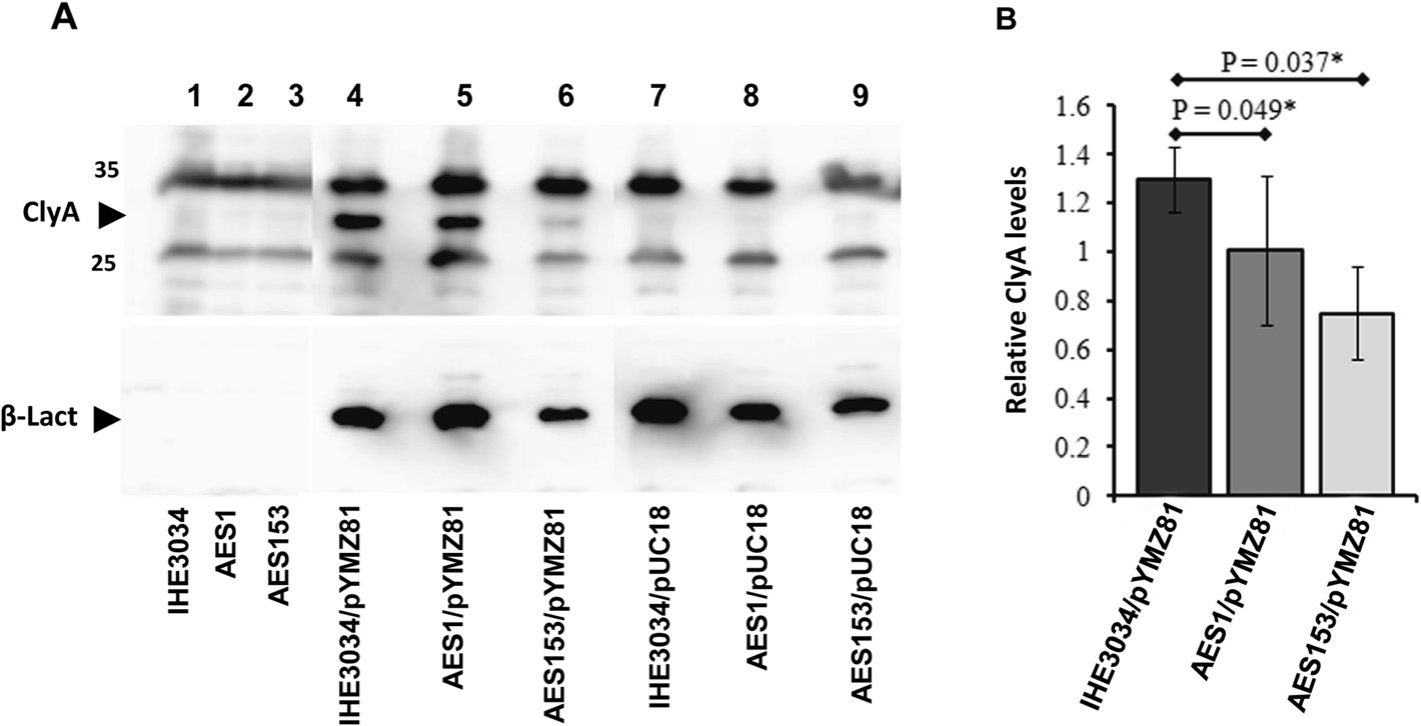
**ClyA protein levels in derivatives of ExPEC strain IHE3034. (A)** Effect of the *sfaX-Y* genes on the relative levels of ClyA protein. Samples were taken from cultures grown in LB medium at 37°C of the following strains: lane 1, IHE3034 (*sfaX* ^*+*^); lane 2, AES1 (*sfaX::kan*); lane 3, AES153 (∆ *sfaY*); lane 4, IHE3034/pYMZ81; lane 5, AES1/pYMZ81; lane 6, AES153/pYMZ81; lane 7, IHE3034/pUC18; lane 8, AES1/pUC18; lane 9, AES153/pUC18. The immunoreactive bands corresponding to ClyA and the plasmid encoded β - Lactamase (used as the control in this experiment) are indicated with arrowheads. **(B)** Quantification of relative ClyA levels in the strains IHE3034/pYMZ81 and AES1/pYMZ81 calculated from the ratios of ClyA and β –Lactamase levels as monitored in the image analyzer (see Methods). The data represent the average value of three independent experiments and vertical bars indicate the standard deviations. An asterik indicates significance in difference according to Student’s *t*-test.

Recent studies have shown that there are regulatory proteins (the SfaX/PapX protein family) that show resemblance to the SlyA protein and are encoded by genes in fimbrial gene clusters typically occurring in UPEC isolates but which are absent in *E. coli* K-12 [40-42]. We therefore decided to investigate if the SlyA-like regulator SfaX might influence the expression of the *clyA* locus. In UPEC strain 536 there are two fimbrial gene clusters, *sfa* and *prs* that include genes for such regulatory proteins, SfaX and PrsX, respectively. In order to specifically test if the SfaX protein can influence expression of *clyA* we performed tests with the *sfaX*^*+*^ wildtype and a mutant derivative of the ExPEC strain IHE3034 which is known to have only one copy of the *sfaX* gene family [41]. A plasmid construct with the *clyA*^*+*^ gene under control of its natural promoter region was introduced into strain IHE3034 and the *sfaX::kan* derivative AES1. A mutant derivative (AES153) defective in the *sfaY* gene located immediately upstream of *sfaX* in the same operon was also included. The SfaY protein is predicted to function as a c-di-GMP phosphodiesterase and thereby indirectly involved also in the activity of the transcriptional regulator SfaX [41]. It is also postulated that the *sfaY* mutation is causing a polar effect that may reduce *sfaX* expression. The phenotypic test of ClyA-mediated hemolytic activity showed that colonies of the wildtype strain IHE3034 caused stronger hemolysis than the mutant derivatives indicating that its expression and/or release of ClyA was higher than that of AES1 and AES153 (Table 3). Western immunoblot analysis of ClyA protein levels, using the plasmid encoded enzyme β-lactamase as reference, indicated that the *sfaX*^*+*^*sfaY*^*+*^ wild type bacteria produced a somewhat higher level of ClyA than the mutant derivatives (Figure 5).

**Table 3:** **Phenotypic test performed on blood agar plate** ^**1)**^

**Bacterial strain**	**Hemolytic phenotypes**
IHE3034 (*sfaX* ^*+*^ *)*	-
AES1 (*sfaX::kan*)	-
AES153 (∆ *sfaY*)	-
IHE3034/pYMZ81	+++
AES1/pYMZ81	++
AES153/pYMZ81	+
IHE3034/pUC18	-
AES1/pUC18	-
AES153/pUC18	-

^1)^The strains were grown on double blood agar plate and incubated overnight at 37°C prior to analysis of the phenotypes. (+++ = Strong hemolysis, ++ = Weak hemolysis, + = very weak hemolysis and - = No hemolysis).

**Figure 6:**
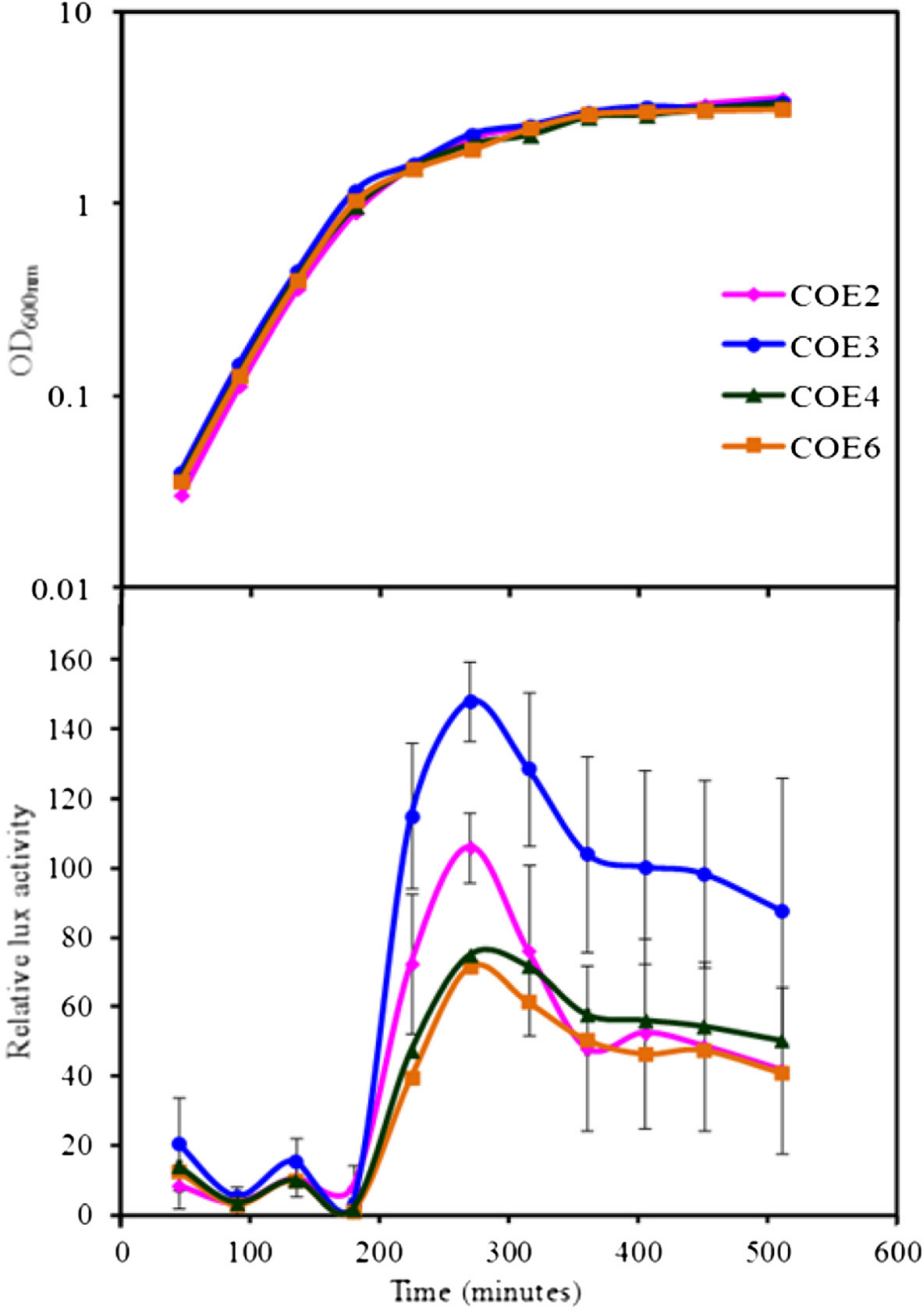
**Effect of SfaX protein overproduction on expression of** ***clyA.*** Transcription of *clyA* was monitored by luciferase measurements with the *clyA::luxAB* UPEC restored construct COE2 carrying different plasmids as follows: COE2 carrying the *sfaX* ^*+*^ plasmid clone pAES1 (COE3), COE2 carrying the vector pBR322 (COE4) and COE2 carrying the *papI* ^+^ plasmid clone pHMG95 (COE6). The bacteria were grown in LB medium (Figure 6) and the growth curve data are from representative experiments. The data showing relative Lux activities represent the average values of three independent experiments and vertical bars indicate the standard deviations.

As a more direct assessment of the potential influence of the *sfaX/papX* regulatory gene products on *clyA* gene transcription in a UPEC strain we tested what effect overproduction of SfaX protein might have. A plasmid with the cloned *sfaX*^*+*^ gene was introduced into the UPEC derivative COE2 carrying the *clyA::luxAB* operons construct in the chromosome. For comparison we included a test with another regulator, the PapI protein known to be part of a regulatory protein complex at the major promoter region of the *pap* and *sfa* fimbrial gene clusters in UPEC isolates [41]. The strain carrying the plasmid with the *sfaX*^*+*^ gene (pAES1) was named COE3, a vector plasmid control strain was named COE4, and the strain carrying the plasmid with the *papI*^*+*^ gene (pHMG95) was named COE6. Samples for Luciferase activity measurements were taken during the growth of these strains in LB medium (Figure 6A). The results demonstrated that the UPEC strain carrying the *sfaX*^*+*^ plasmid (strain COE3) had a greatly enhanced level of *clyA* transcription. No such effect was seen with the *papI*^*+*^ clone (strain COE6). These results strongly support the suggestion that the presence of SfaX protein in UPEC isolates can cause up-regulation of *clyA* gene expression and that the effect could be stronger with higher levels of SfaX/PapX proteins in such *E. coli* strains.

### The *clyA*^+^ derivative of UPEC strain *E. coli* 536 has increased susceptibility to the antimicrobial peptide Polymyxin B

**Figure 7:**
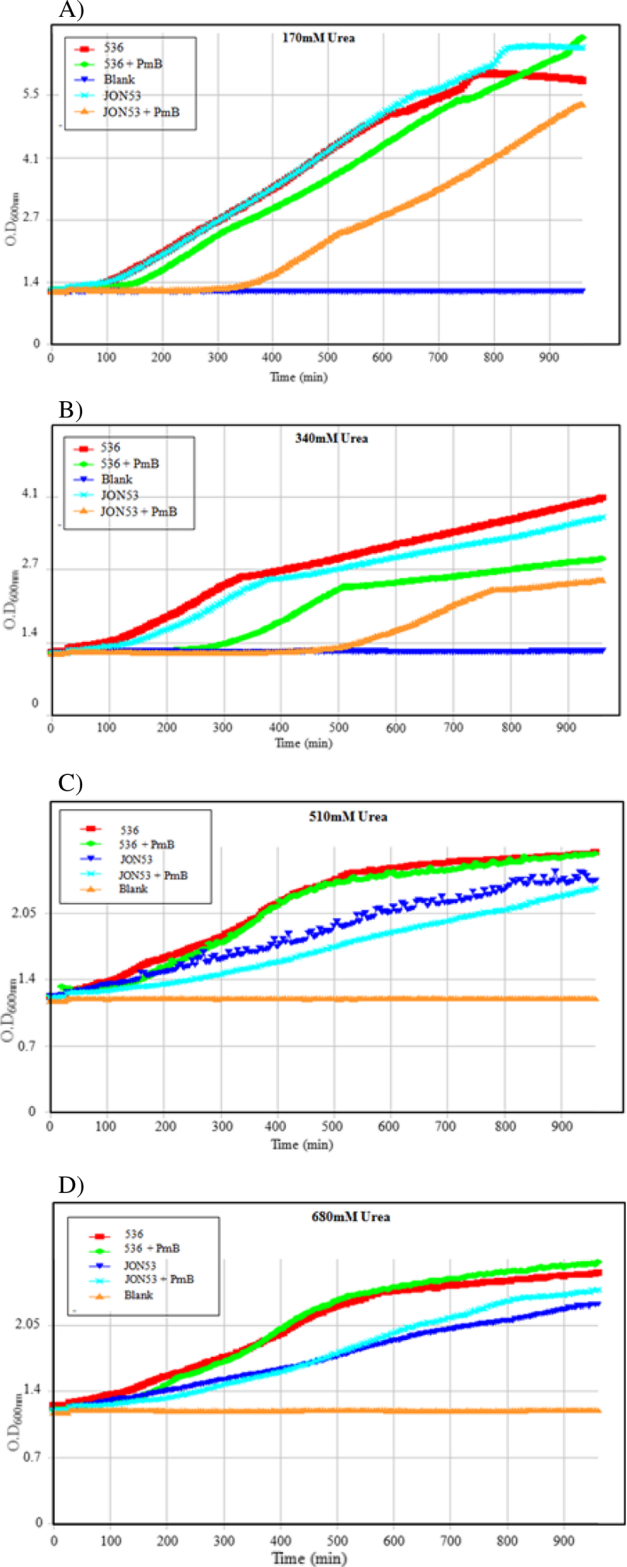
**Effect of Polymyxin B at a sub-inhibitory concentration on the growth of UPEC strain 536 and the** ***clyA*** ^***+***^ **derivative JON53.** Note that the color codes for the strain JON53 and the blank are changed between panels **A**-**B** to **C**-**D**. The growth was monitored as optical density at OD_600nm_ and the plots show growth in presence of 0.39 μg/ml of Polymyxin B which is a concentration at half the observed MIC value (0.78 μg/ml) in AUM medium with the following concentrations of urea: **A)** 170 mM; **B)** 340 mM; **D)** 510 mM; **D)** 680 mM.

Considering the relatively high expression levels of the pore-forming protein in the UPEC derivatives, we decided to test the possibility that restoration of the *clyA* locus in strain 536 might influence its cell wall or membrane properties and perhaps change its susceptibility to antimicrobial compounds directed towards membranes. The strain JON53 was therefore cultured in medium supplemented with serially diluted concentrations of different antimicrobial peptides (Polymyxin B, β-defensin and LL-37), and with different concentrations of urea or creatinine. We compared the MIC values for each antimicrobial compound with the parental strain 536 and the *clyA*^+^ derivative JON53. Our preliminary studies indicated that there was no difference in MIC values between the two strains for either of the tested compounds (Polymyxin B, β-defensin or LL-37; data not shown). Likewise, there were no differences detected when different concentrations of creatinine in the medium were tested. However, when a sub-inhibitory concentration (0,39 μg/ml, i.e. half the concentration of the observed MIC value 0,78 μg/ml) was present in the medium the *clyA*^+^ derivative JON53 displayed a somewhat slower growth in presence of Polymyxin B, manifested as a more prolonged lag, than the parental UPEC strain 536 (Figure 7A). The normal AUM medium contained 170 mM urea. At a two-fold higher concentration (340 mM) of urea also the parental UPEC strain 536 seemed more susceptible to the effect of the sub-inhibitory concentration of Polymyxin B (Figure 7B). At even higher concentrations of urea (510 mM or 680 mM) the presence of Polymyxin B *per se* did not seem to alter the growth much but both strains showed reduced growth. Notably, it was evident that the higher concentrations of urea affected growth of the *clyA*^+^ derivative JON53 more than that of the parental *ΔclyA* UPEC isolate (Figure 7C & D).

The effect of Polymyxin B on growth was also monitored with the *E. coli* K-12 strains MC4100 and MWK11, the latter being a derivative of MC4100 with a constitutive expression of ClyA protein due to an improved binding site near the promoter for the cAMP-CRP activating complex [8]. These strains showed a similar MIC value (0,78 μg/ml) for Polymyxin B as the UPEC strains. In the growth test with Polymyxin B at half the MIC value (0,39 μg/ml) the higher ClyA expression did not cause any apparent disadvantage but when Polymyxin B was added at a somewhat higher concentration (0,48 μg/ml) the ClyA^+^ phenotype was correlated to reduced growth also in the case of the *E. coli* K-12 derivatives (see Additional file 2).

## Discussion and conclusions

In *Escherichia coli* several different pore-forming cytolysins have been identified. The one most extensively studied is α-hemolysin (HlyA), which is produced by many uropathogenic *E. coli* (UPEC) strains and contributes to virulence as shown in several animal models [43]. The biological role of the ClyA protein, representing a novel family of non-RTX toxins in *Enterobacteriaceae*, remains to be discovered, although the distribution of the *clyA* locus in a wide array of isolates would indicate that it has an evolutionarily conserved function. The *clyA* gene is clearly conserved in Enterobacteria, i. e., in many *E. coli* isolates and in typhoid serovars of *Salmonella enterica* [2,6,9,10].

A functional *clyA* gene was evidently lost through deletion(s) in several *E. coli* strains, e. g., in 19 of the 72 ECOR strains, and the Δ*clyA* alleles were present in a number of different variants. Some strains carried various deletions in the *clyA* coding sequence and in the upstream region, resulting in truncated *clyA* loci. In the majority of cases (Δ*clyA* versions I, II and III), the function of the *clyA* locus may be effectively abolished by two deletions, removing the promoter region and portions of the N-terminal and central regions of the coding sequence. In ECOR43 and ECOR44, a 12-bp in-frame deletion (corresponding to amino acids 183–186 in ClyA) results in the expression, at a level similar to that of the *clyA*^*+*^ derivative JON53, of a ClyA polypeptide that was found to have no or very low hemolytic activity (our unpublished data). The presence of short repetitive DNA sequences at the junctions of the major 493-bp deletion in Δ*clyA* variants I-III (Figure 1A) and the 12-bp deletion of Δ*clyA* variant IV (Figure 1B) suggests that these deletions may have been formed as a result of slippage mispairing. The role of direct repeats in deletion formation has been demonstrated by sequence analysis of several deletion mutants in *E. coli* [44-46]. Since no such repeats were identified at the junctions of the other deletions, i. e. the 203- or 217-bp deletions in Δ*clyA* variants I-III (Figure 1B), these deletions appear to have arisen by some other mechanism(s). They could be the result of subunit exchange between DNA gyrase molecules, since this model has been suggested for deletions terminating in sequences that are neither directly nor inversely repeated [47,48]. It also appears that after the functionality of a *clyA* locus was lost, further alterations occurred, e.g., insertion of an IS-2 element (strains ECOR61 and ECOR62), and non-conservative point mutations in the *clyA* coding sequence.

Considering the possibility that there might be some patho-adaptive selection for mutations in *clyA* of some *E. coli* we restored, and studied the expression of, a functional gene locus in the chromosome of the UPEC isolate 536.

In order to determine if regulatory genes of fimbrial gene clusters typically present in the chromosome of UPEC can upregulate the expression of *clyA*, a plasmid carrying the *sfaX* gene was introduced into the restored UPEC derivative and we observed that the level of ClyA was upregulated at the early stationary phase of growth. Therefore, the *sfaX* gene does upregulate *clyA* expression.

Although the prevalence of *E. coli* strains with an intact *clyA* gene in the ECOR A and B1 groups would support the argument that ClyA has no direct role in virulence, it cannot be ruled out that pathogenic rather than non-pathogenic isolates under certain conditions may express higher levels of ClyA and/or more efficiently translocate the protein. In contrast to the commensal strains, extraintestinal pathogenic *E. coli* are mostly derived from group B2 and, to a lesser extent, from group D [17-19]. There is a phylogenetically clustered distribution of the virulence-associated determinants alpha-hemolysin (*hly*), type-II capsule (*kps*), and P (*pap*) and S (*sfa*) fimbriae in the *E. coli* B2 and D groups [18], and it has been suggested that the B2 strains should be considered highly virulent as evidenced by a mouse model of extra-intestinal virulence [19]. Hence, the fact that every one of the 15 strains in the B2 group of the ECOR collection contained a Δ*clyA* allele [22] strongly suggests that *E. coli* strains adapted for an extraintestinal lifestyle have the *clyA* gene deleted. On the other hand, it does not necessarily imply that some selective condition(s) or mechanism(s) must be found in the extraintestinal locations where *E. coli* may reside.

In a study using multilocus enzyme electrophoresis and sequencing of the *mdh* housekeeping gene, it was concluded that pathogenic strains of *E. coli* do not have a single evolutionary origin but have arisen on several occasions [49]. Similarly, it appears unlikely that there is a common ancestor for all Δ*clyA* strains, e. g. two ECOR strains in the A group and one in the E group were Δ*clyA*. The presence of different variants of the truncated *clyA* locus suggests that *clyA* is a preferred target for deletion mutations, and that such deletions have arisen on several independent occasions. The adaptation to a particular host may involve loss of virulence traits that are important for infection of a wider spectrum of animals [50]. Similarly, it is possible that the *clyA* locus could be inactivated in the process of acquisition of new genes (e. g., genes in PAIs) that would promote transition to an extra-intestinal lifestyle. One hypothesis consistent with our present findings would be that the strict regulation of *clyA* could be lost during this transition. In *E. coli* K-12 the *clyA* locus is strictly regulated and its expression is silenced by the H-NS protein [39]. One plausible explanation for the strict regulation in case of such genes appearing as cryptic/silenced would be that less strictly controlled alleles of such genes are disadvantageous to the bacteria under certain environmental conditions [51]. In this context it may be interesting to carry out further studies with strains from ECOR group D that seem to have an intact *clyA*^*+*^ locus whereas it remains to be established if they produce ClyA protein and how expression is controlled.

In previous studies, it was demonstrated that epithelial cells of the urinary tract in humans and mice secrete an antimicrobial peptide, cathelicidin, upon contact with UPEC and that the severity of the bacterial invasion is linked to bacterial resistance to cathelicidin [52]. While we did not detect any effect of the tested cathelicidin (LL-37) on the growth of the bacteria, it remains to be determined if the *clyA*^*+*^ UPEC derivative is more susceptible to such peptides under *in vivo* conditions. We may hypothesize that the mutations in the *clyA* gene were selected for either under growth-limiting antimicrobial peptide stress conditions in the urinary tract to which bacterial cells are maladapted or under some other condition of stress for example in the intestine where likewise the combination of a *clyA*^*+*^ allele and some other trait(s) might be unfavourable. The mutations in the *clyA* gene may adapt the bacteria to this stress condition and thereby contribute to the bacterial survival as pathoadaptive events. The constructs described in this work should allow us to test such hypotheses in suitable model infection systems.

In order to test the potential impairment of membrane integrity in the restored strain, the JON53 strain was cultured in presence of the antimicrobial peptide Polymyxin B which can disrupt the integrity of the bacterial cell membrane by interacting with its phospholipids. The MIC value for Polymyxin B of JON53 was not altered in comparison with the wild type UPEC strain 536 although there was a clearly detectable negative effect on the growth of the *clyA*^*+*^ derivative JON53 when Polymyxin B was added at a sub-inhibitory concentration. Also higher concentrations of urea in the medium caused this differential effect on the growth of the two strains.

Although a rather preliminary finding that will need to be studied further, the observed higher susceptibility to the antimicrobial peptide Polymyxin B that was seen in the case of the UPEC with a restored *clyA*^*+*^ locus would suggest that such a phenotype is more of a disadvantage in the intestinal locations where other bacteria, e.g. *Bacillus*, are producing antimicrobial peptides.

## Methods

### Bacterial strains and growth conditions

Sources and relevant characteristics of the bacterial strains and plasmids used in this study are listed in Table 1 and Table 4, respectively. Bacterial strains were grown aerobically at 37°C on LB broth solidified with 1.5% (w/v) agar, or in Luria-Bertani (LB) broth, Poor Broth (PB) [53], or artificial urine medium (AUM) [54]. Blood agar plates consisted of 5% horse erythrocytes solidified with 1% (w/v) Columbia-Agar (base) (Merck), which according to the manufacturer contains 2.3% (w/v) special nutrient substrate. Ca^2+^ depleted conditions were obtained by supplementing the blood agar plates with Na-oxalate (final concentration 2 mM). Antibiotic selection was performed using 30 μg/ml kanamycin, 12.5 μg/ml chloramphenicol, 50 μg/ml carbenicillin or 15 μg/ml tetracycline.

**Table 4:** **Plasmids used in this work**

**Plasmid**	**Relevant characteristics**	**Reference/source**
pBR322	Cloning vector, Tc^r^	[55]
pGEM®-T Easy	T-vector for cloning of PCR-fragments, Cb^r^	Promega
pJON77	3.1-kb subclone of *clyA* in pSL1180, Cb^r^	This work
pJON78	3.1-kb subclone of *clyA* in pKO3, Cm^r^	This work
pJON176	*clyA* ^+^ *kan* in pJON78, Cm^r^ Km^r^	This work
pKO3	Gene replacement vector, Cm^r^	[56]
pMWK4	*clyA::luxAB* in pCH257, Cm^r^	[8]
pAES1	pBR322, *sfaX*_II_ gene from *sfa*_II_ operon	[41]
pHMG95	pBR322, *lacP*(UV5)*papI* ^*+*^ clone	[57]
pSL1180	Cloning vector, Cb^r^	[58]
pUC4K	Kanamycin resistance gene cartridge plasmid, Kan^r^	[59]
pUC18	Cloning vector, Cb^r^	[60]
pYMZ81	A 1.6-kb *clyA* locus in pUC18	[8]
pYMZ62	3.5-kb subclone of *clyA* in pUC18	[8]

### Genetic techniques

Oligonucleotides were obtained from DNA Technology, Aarhus, Denmark or from TAG Copenhagen, Copenhagen, Denmark. DNA sequencing was performed using the ABI PRISM™ Dye Terminator Cycle Sequencing Ready Reaction Kit with AmpliTaq® DNA Polymerase, and an ABI PRISM™ 377 DNA Sequencer. For PCR-screening, cloning and sequencing of *clyA*-like genes we used the oligonucleotide primers umu1 (5′-AATATTTGTCGCTGC-3′) and p79 (5′-TGTCAACAGGTAACTCTC-3′). The primers umu1 and p79 amplify a 1292-bp fragment starting 293 bp upstream of the *clyA* start codon and ending 87 bp downstream of the stop codon, based on the sequence of the *E. coli* K-12 *clyA* locus [2,61] and our data. Cloning of PCR-amplified *clyA*-like sequences was performed using the pGEM®-T Easy Vector System of Promega, as specified by the manufacturer, and DH5α as a host strain.

### Plasmid and strain construction

To construct *clyA*^+^ derivatives of *E. coli* 536 and J96, we used the suicide plasmid pJON176 containing the *clyA* wild type allele and a kanamycin resistance cassette located 350 bp downstream of the *clyA* stop codon. The construction of pJON176 was done as follows: A 3.5-kb *Pvu*II restriction fragment from plasmid pYMZ62 containing the *clyA* locus region (between nucleotide positions 1,227,641 and 1,231,223 in the *E. coli* K-12 genome) was inserted into *Eco*RV-digested pSL1180 which resulted in the construction of pJON77. The plasmid pJON77 was digested to yield a 3.2-kb *Pvu*II-*Bam*HI fragment that was subsequently ligated into the *Bam*HI-*Sma*I-digested pKO3 suicide donor plasmid thus resulting to the construct pJON78. A 1.3-kb *Pst*I restriction fragment which contains the kanamycin resistance gene from the plasmid pUC4K was then ligated into the *Nsi*I restriction site of pJON78, i. e. 350 bp downstream of the *clyA* stop codon, to generate the construct pJON176. The clone included the entire 0,4 kb intercistronic region and sequences into the *umuD* gene upstream of *clyA* such that the otherwise deleted promoter region in the UPEC strains could be restored. Using pJON176 the *clyA* locus and kanamycin resistance gene was introduced into the chromosome of the *E. coli* strains 536 and J96, as previously described [54], to generate the strains JON53 and JON47, respectively.

For the purpose of quantitatively determining the level of *clyA* gene transcription, we introduced a *clyA-luxAB* operon construct using the suicide plasmid pMWK4 [8]. The pMWK4 plasmid contains DNA corresponding to the sequence 290 bp upstream of *clyA* and 76 bp into the *clyA* coding sequence. The pMWK4 plasmid was integrated in tandem to *clyA* in the chromosome of JON53 by a single recombination event. The resulting strain was designated COE2 (JON53 - *clyA::luxAB*).

The plasmids pAES1 and pHMG95 were introduced into the strain COE2 by electroporation and the transformants were selected on LB agar plates containing 12.5 μg/ml chloramphenicol, 30 μg/ml kanamycin and 50 μg/ml carbenicillin as appropriate. The resulting strains were designated COE3 (COE2/pAES1), COE4 (COE2/pBR322) and COE6 (COE2/pHMG95).

### ClyA expression assays

Lytic activity of the UPEC and K-12 strains were analysed using a double horse blood agar plate. The blood agar plate was supplemented with 2 mM of Sodium–oxalate ( Ca^2+^ chelator ). Bacteria were streaked into vertical lines across the plate and 1.5 μl of 0.5 mg/ml of Mitomycin C was dripped onto the horizontally streaked rows of strains prior to incubation at 37°C for maximum 16 hours. Mitomycin C triggers the bacterial SOS response and hence induces the proliferation of lysogenic phages (Walker, 1996) and thus may cause release of cytolysin A that lyses the blood [9].

### Immuno-fluroescence assay

Approximately five bacterial colonies grown on an LB plate overnight were suspended in 100 μl water or PBS, and 10 to 20 μl of the suspension was placed on a glass slide and air-dried. The cells were fixed with 4% paraformaldehyde (PFA) in PBS for 10 minutes and wash with PBS. The slides were covered for 20 min with 0.1 M glycine diluted in PBS, followed by washes in PBS then blocked with 1% BSA in PBS for 30 min. Polyclonal ClyA antiserum was diluted in a blocking solution and then added to the bacteria on the slide at a final dilution of 1:3,000 and incubated for 2 hours at room temperature or overnight in 4°C. Alexa Fluor 555 anti-rabbit IgG (Invitrogen) diluted 1:500 in blocking solution was applied to the bacterial cells for 1 hour at room temperature. Washes in 1 × PBS for 15 min were repeated five times and the bacteria were then mounted in a fluorescence mounting medium (Dako). The slides were examined at 1000× magnification with a Nikon Intensilight C-HGFI system microscope equipped for fluorescence; the images were obtained with a Hamamatsu DRCA-ER camera.

### Measurement of luciferase (lux) activity

Colonies of the bacterial strains MG1655 (K-12), JON53-*clyA*^*+*^*::luxAB* (COE2), COE3, COE4 and COE6, from plates incubated overnight were inoculated into LB broth medium. The bacteria were cultured aerobically at 37°C. The optical density (OD_600nm_) was measured during growth at different time points. In parallel, the transcriptional activity was measured in a Sirius luminometer using decanal as a substrate. One hundred μl of a 0.1% v/v suspension of decanal (Sigma) in water was added to 100 μl of bacterial culture as programmed by the Sirius instrument and light emission was measured. Triplicates were measured for each sample and specific activity (lux activity units/OD_600nm_) was calculated and plotted.

### Sub-cellular localization of ClyA in K-12 and UPEC derivatives

Sub-cellular fractionation was performed essentially as explained before [62]. For sub-cellular localization of ClyA, *E. coli* cells were grown in LB medium at 37°C until late logarithmic phase (OD_600nm_ ≈ 2). To prepare whole cell lysate fractions, the bacteria (1 ml) were centrifuged at 12,000 × g for 5 min and the bacterial pellet was resuspended in (80 ul) 20 mM Tris–HCl pH 8.0 buffer. SDS-polyacrylamide gel electrophoresis (SDS-PAGE) analyses of proteins were performed as described previously [63]. Five μl (from 80 μl) of the bacterial suspensions were loaded in the well. To prepare periplasmic fractions, bacterial suspensions (1.5 ml) with a cell density of approximately 5 × 10^9^/ml were harvested by centrifugation. The pellet was washed twice with 10 mM Tris–HCl (pH 8.0) three times and resuspended in 20 mM Tris–HCl (pH 8.0), 20% (wt/vol) sucrose, and 0.1 mM EDTA at 25°C. After 10 min the cells were pelleted and resuspended in sterile distilled water. After incubation on ice for 10 min, the cells were removed by centrifugation at 12,000 × g. The supernatant was used as the periplasmic fraction. Periplasmic proteins were concentrated by precipitation with ice-cold 10% trichloroacetic acid containing 1 mg/ml deoxycholate. The precipitated proteins were collected by centrifugation at 12,000 × g, washed with acetone, dried under vacuum, and dissolved in sample buffer (50 mM Tris–HCl (pH 6.8), 10% glycerol, 5% β-mercaptoethanol, 2% sodium dodecyl sulfate (SDS), 0.05% bromophenol blue). Samples were neutralized by addition of saturated Tris solution and boiled for 5 min at 100°C.

### Western blot analysis

Western immunoblotting was performed as previously described [64]. The proteins were detected using different primary polyclonal antisera: polyclonal anti-ClyA antiserum [6], and the anti-CRP polyclonal antiserum [62] recognizing *E. coli* cyclic AMP receptor protein (CRP), which was used as an internal control for cytoplasmic protein and antiserum recognizing TEM-β lactamase [62] which was used as the periplasmic protein loading control for strains carrying carbenicillin resistance plasmids. The immunoreactive bands were visualized by scanning using a luminescent image analyzer LAS 4000 IR multi-color (Fujifilm) and/or by exposure on the Hyper film (Amersham Biosciences).

### Antimicrobial assays

MIC determination: Liquid growth inhibition assays were performed essentially as described earlier [53]. Briefly, ~5 bacterial colonies from an overnight culture plate were suspended in Poor Broth (PB) medium (1% Bactotryptone, pH 7.5) or artificial urine media (AUM) and grown aerobically at 37°C overnight. Bacterial suspensions were diluted to an OD_600nm_ = 0.01 and grown to approximately OD_600nm_ 0.2 - 0.4 to obtain an exponential phase culture. In a flat-bottom 96-well plate (Falcon), 95 μl of the medium was added to all wells, 5 μl of the bacterial culture (OD_600nm_ = 0.2) was added to each well at a final OD_600nm_ of 0.001 (1 × 10^5^ cells/110 μl) and a 10 μl sample of two-fold serially diluted solutions of the peptide (polymyxin B, β-defensin or LL-37) was added to obtain a range from highest (50 μg/ml) to lowest (0.097 μg/ml) concentrations in the series of wells containing bacteria and medium. To determine the effects of some of the components in AUM, media containing higher concentrations of urea (i.e. 340 mM – 680 mM) and twice the concentration of creatinine (i.e. 14 mM) were used.

The positive control wells contained only bacteria and medium while the negative control well contained medium and water. The plate was incubated for 16 hours aerobically at 37°C in a TECAN Infinite M200 fluorescence multiplate reader and bacterial growth was monitored by measuring absorbance at OD_600nm_ at 5 minute intervals. MIC values are defined as the lowest concentration that causes 100% growth inhibition. The following Polymyxin B susceptible and resistant organisms were used as controls: *V. cholerae* strain 569B (MIC = 1.5 μg/ml), *V. cholerae* strain A1552 (MIC = 50 μg/ml), *E. coli* strain MC4100 (MIC = 7.8 μg/ml), *E. coli* strain MWK11 (MIC = 7.8 μg/ml).

## Additional files

Additional file 1:
**The open access software programs BLAST (** http://blast.ncbi.nlm.nih.gov/Blast.cgi **) to search for sequences and strains with similarities to the** ***clyA*** **/** ***hlyE*** **gene, and ClustalW (** http://pbil.ibcp.fr/htm/index.php **) were used for alignment of the sequences with the** ***clyA*** **gene sequences of** ***E. coli*** **strain MG1655 (accession number of NC_000913.3) as the reference sequence.** It includes the 1.2 kb region between nucleotide coordinates 1229483 and 1230694 and represents the entire *clyA* coding sequence and 300 base pairs upstream of the ATG start codon. The sequence of *clyA* was compared with the corresponding sequences of *clyA* in other *E. coli* strains (both IPEC and ExPEC). Black nucleotide lettering and dashes indicates position where there are differences in the otherwise red letter compilation. The translational start sequences (ATG and ribosome binding motif) are marked in green. The transcriptional start sequences −35 and −10 motifs and the initiating nucleotide are marked in blue. The binding site for the CRP/FNR regulators is marked in grey. The binding sequences for SlyA are underlined. Additional file 2:
**Effect of Polymyxin B on the growth of** ***E. coli*** **K-12 derivatives MC4100 and MWK11 in batch cultures of medium AUM at 37°C.**
