# Supplementary material for: Elevated recombinant clyA gene expression in the uropathogenic Escherichia coli strain 536, a clue to explain pathoadaptive mutations in a subset of extraintestinal E. coli strains
Source: BMC Microbiol. 2014 Sep 2;14:216. doi: 10.1186/s12866-014-0216-4 (PMC4164713; doi:10.1186/s12866-014-0216-4)
Supplement: Additional file 1: — The open access software programs BLAST ( http://blast.ncbi.nlm.nih.gov/Blast.cgi ) to search for sequences and strains with similarities to the clyA / hlyE gene, and ClustalW ( http://pbil.ibcp.fr/htm/index.php ) were used for alignment of the sequences with the clyA gene sequences of E. coli strain MG1655 (accession number of NC_000913.3) as the reference sequence. It includes the 1.2 kb region between nucleotide coordinates 1229483 and 1230694 and represents the entire clyA coding sequence and 300 base pairs upstream of the ATG start codon. The sequence of clyA was compared with the corresponding sequences of clyA in other E. coli strains (both IPEC and ExPEC). Black nucleotide lettering and dashes indicates position where there are differences in the otherwise red letter compilation. The translational start sequences (ATG and ribosome binding motif) are marked in green. The transcriptional start sequences −35 and −10 motifs and the initiating nucleotide are marked in blue. The binding site for the CRP/FNR regulators is marked in grey. The binding sequences for SlyA are underlined. [file 12866_2014_216_MOESM1_ESM.pdf]

```
                                     10        20        30        40        50        60
                                      |         |         |         |         |         |
MG1655 (1229483)..   GGCTATCAATATTTGTCGCTGCATAGGCTGCTGATTTTTCGTTCTCTTATCTTGTGCTCA
MDS42                GGCTATCAATATTTGTCGCTGCATAGGCTGCTGATTTTTCGTTCTCTTATCTTGTGCTCA
W3110                GGCTATCAATATTTGTCGCTGCATAGGCTGCTGATTTTTCGTTCTCTTATCTTGTGCTCA
DH1                  GGCTATCAATATTTGTCGCTGCATAGGCTGCTGATTTTTCGTTCTCTTATCTTGTGCTCA
ETEC                 GGCTATCAATATTTGTCGCTGCATAGGCTGCTGATTTTTCGTTCTCTTATCTTGTGCTCA
P12b                 GGCTATCAATATTTGTCGCTGCATAGGCTGCTGATTTTTCGTTCTCTTATCTTGTGCTCA
BW2952               GGCTATCAATATTTGTCGCTGCATAGGCTGCTGATTTTTCGTTCTCTTATCTTGTGCTCA
DH10B                GGCTATCAATATTTGTCGCTGCATAGGCTGCTGATTTTTCGTTCTCTTATCTTGTGCTCA
C321                 GGCTATCAATATTTGTCGCTGCATAGGCTGCTGATTTTTCGTTCTCTTATCTTGTGCTCA
Wxxxxx20             GGCTATCAATATTTGTCGCTGCATAGACTGCTGATTTTTCGTTCTCTTATCCTGTGCTCA
KO11FL               GGCTATCAATATTTGTCGCTGCATAGACTGCTGATTTTTCGTTCTCTTATCCTGTGCTCA
LY180x19             GGCTATCAATATTTGTCGCTGCATAGACTGCTGATTTTTCGTTCTCTTATCCTGTGCTCA
E24377A              GGCTATCAATATTTGTCGCTGCATAGACTGCTGATTTTTCGTTCTCTTATCCTGTGCTCA
SE11xx14             GGCTATCAATATTTGTCGCTGCATAGACTGCTGATTTTTCGTTCTCTTATCCTGTGCTCA
2009EL               GGCTATCAATATTTGTCGCTGCATAGACTGCTGATTTTTCGTTCTCTTATCCTGTGCTCA
2011C_3493           GGCTATCAATATTTGTCGCTGCATAGACTGCTGATTTTTCGTTCTCTTATCCTGTGCTCA
55989x18             GGCTATCAATATTTGTCGCTGCATAGACTGCTGATTTTTCGTTCTCTTATCCTGTGCTCA
IAI1xx13             GGCTATCAATATTTGTCGCTGCATAGACTGCTGATTTTTCGTTCTCTTATCCTGTGCTCA
O103H2               GGCTATCAATATTTGTCGCTGCATAGACTGCTGATTTTTCGTTCTCTTATCCTGTGCTCA
APECxx11             GGCTATCAATATTTGTCGCTGCATAGACTGCTGATTTTTCGTTCTCTTATCCTGTGCTCA
UMNK88               GGCTATCAATATTTGTCGCTGCATAGACTGCTGATTTTTCGTTCTCTTATCCTGTGCTCA
HSxxxx39             GGCTATCAATATTTGTCGCTGCATAGACTGCTGATTTTTCGTTCTCTTATCCTGTGCTCA
BL21DE3              GGCTATCAATATTTGTCGCTGCATAGACTGCTGATTTTTCGTTCTCTTATCCTGTGCTCA
REL606               GGCTATCAATATTTGTCGCTGCATAGACTGCTGATTTTTCGTTCTCTTATCCTGTGCTCA
BL21_GoldDE3pLysS    GGCTATCAATATTTGTCGCTGCATAGACTGCTGATTTTTCGTTCTCTTATCCTGTGCTCA
ATCCxx37             GGCTATCAATATTTGTCGCTGCATAGACTGCTGATTTTTCGTTCTCTTATCCTGTGCTCA
O111H_               GGCTATCAATATTTGTCGCTGCATAGACTGCTGATTTTTCGTTCTCTTATCCTGTGCTCA
O26H11               GGCTATCAATATTTGTCGCTGCATAGACTGCTGATTTTTCGTTCTCTTATCCTGTGCTCA
UMN026               GGCTATCAATATTTGTCGCTGCATAGACTGCTGATTTTTCGTTCTCTTATCCTGTGCTCA
042NC_0176261        GGCTATCAATATTTGTCGCTGCATAGACTGCTGATTTTTCGTTCTCTTATCCTGTGCTCA
IAI39x23             GGCTATCAATATTTGTCGCTGCATAGACTGCTGATTTTTCGTTCTCTTATCCTGTGCTCA
O7K1xx29             GGCTATCAATATTTGTCGCTGCATAGACTGCTGATTTTTCGTTCTCTTATCCTGTGCTCA
SMS_3_5              GGCTATCAATATTTGTCGCTGCATAGACTGCTGATTTTTCGTTATCTTATCCTGTGCTCA
RM12579              GGCTATCAATATTTGTCGCTGCATAGACTGCTGATTTTTCGTTCTCTTATCCTGTGCTCA
CB9615               GGCTATCAATATTTGTCGCTGCATAGACTGCTGATTTTTCGTTCTCTTATCCTGTGCTCA
Xuzhou21             GGCTATCAATATTTGTCGCTGCATAGACTGCTGATTTTTCGTTCTCTTATCCTGTGCTCA
TW14359              GGCTATCAATATTTGTCGCTGCATAGACTGCTGATTTTTCGTTCTCTTATCCTGTGCTCA
EC4115               GGCTATCAATATTTGTCGCTGCATAGACTGCTGATTTTTCGTTCTCTTATCCTGTGCTCA
EDL933               GGCTATCAATATTTGTCGCTGCATAGACTGCTGATTTTTCGTTCTCTTATCCTGTGCTCA
Sakaix28             GGCTATCAATATTTGTCGCTGCATAGACTGCTGATTTTTCGTTCTCTTATCCTGTGCTCA
                     ************************** **************** ******* ********

Prim.cons.           GGCTATCAATATTTGTCGCTGCATAGACTGCTGATTTTTCGTTCTCTTATCCTGTGCTCA

                                     70        80        90       100       110       120
                                      |         |         |         |         |         |
MG1655               CGTGGCCTTCTGGCGACGACGCTCATCCAGCAGAAATGAAAAATATCACCCGGCTAAAAA
MDS42x1              CGTGGCCTTCTGGCGACGACGCTCATCCAGCAGAAATGAAAAATATCACCCGGCTAAAAA
W3110x2              CGTGGCCTTCTGGCGACGACGCTCATCCAGCAGAAATGAAAAATATCACCCGGCTAAAAA
DH1NC_0176381        CGTGGCCTTCTGGCGACGACGCTCATCCAGCAGAAATGAAAAATATCACCCGGCTAAAAA
ETECxx4              CGTGGCCTTCTGGCGACGACGCTCATCCAGCAGAAATGAAAAATATCACCCGGCTAAAAA
P12bxx5              CGTGGCCTTCTGGCGACGACGCTCATCCAGCAGAAATGAAAAATATCACCCGGCTAAAAA
BW2952               CGTGGCCTTCTGGCGACGACGCTCATCCAGCAGAAATGAAAAATATCACCCGGCTAAAAA
DH10Bx7              CGTGGCCTTCTGGCGACGACGCTCATCCAGCAGAAATGAAAAATATCACCCGGCTAAAAA
C321deltaA           CGTGGCCTTCTGGCGACGACGCTCATCCAGCAGAAATGAAAAATATCACCCGGCTAAAAA
Wxxxxx20             CGTGGCCTTCTGGCGACGACGCTCATCCAGCAGAAATGAAAAATATCACCCGGCAAAAAA
KO11FL               CGTGGCCTTCTGGCGACGACGCTCATCCAGCAGAAATGAAAAATATCACCCGGCAAAAAA
LY180x19             CGTGGCCTTCTGGCGACGACGCTCATCCAGCAGAAATGAAAAATATCACCCGGCAAAAAA
E24377A              CGTGGCCTTCTGGCGACGACGCTCATCCAGCAGAAATGAAAAATATCACCCGGCAAAAAA
SE11xx14             CGTGGCCTTCTGGCGACGACGCTCATCCAGCAGAAATGAAAAATATCACCCGGCAAAAAA
2009EL_2050          CGTGGCCTTCTGGCGACGACGCTCATCCAGCAGAAATGAAAAATATCACCCGGCAAAAAA
2011C_3493           CGTGGCCTTCTGGCGACGACGCTCATCCAGCAGAAATGAAAAATATCACCCGGCAAAAAA
55989x18             CGTGGCCTTCTGGCGACGACGCTCATCCAGCAGAAATGAAAAATATCACCCGGCAAAAAA
IAI1xx13             CGTGGCCTTCTGGCGACGACGCTCATCCAGCAGAAATGAAAAATATCACCCGGCAAAAAA
O103H2               CGTGGCCTTCTGGCGACGACGCTCATCCAGCAGAAATGAAAAATATCACCCGGCAAAAAA
APECxx11             CGTGGCCTTCTGGCGACGACGCTCATCCAGCAGAAATGAAAAATATCACCCGGCAAAAAA
UMNK88               CGTGGCCTTCTGGCGACGACGCTCATCCAGCAGAAATGAAAAATATCACCCGGCAAAAAA
```

```
HSxxxx39              CGTGGCCTTCTGGCGACGACGCTCATCCAGCAGAAATGAAAAATATCACCCGGCAAAAAA
BL21DE3               CGTGGCCTTCTGGCGACGACGCTCATCCAGCAGAAATGAAAAATATCACCCGGCAAAAAA
REL606                CGTGGCCTTCTGGCGACGACGCTCATCCAGCAGAAATGAAAAATATCACCCGGCAAAAAA
BL21_GoldDE3pLysS     CGTGGCCTTCTGGCGACGACGCTCATCCAGCAGAAATGAAAAATATCACCCGGCAAAAAA
ATCCxx37              CGTGGCCTTCTGGCGACGACGCTCATCCAGCAGAAATGAAAAATATCACCCGGCAAAAAA
O111H_                CGCGGCCTTCTGGCGACTACGCTCATCCAGCAGAAATGAAAAATATTACCCGGCTAAAAA
O26H11                CGCGGCCTTCTGGCGACTACGCTCATCCAGCAGAAATGAAAAATATTACCCGGCTAAAAA
UMN026                CGCGGCCTTCTGGCGACGACGCTCATCCAGCAGAAATGAAAAATATCACCCGGCTAAAAA
042NC_0176261         CGCGGCCTTCTGGCGACGACGCTCATCCAGCAGAAATGAAAAATATCACCCGGCTAAAAA
IAI39x23              CGCG-CCTTCTGGCGACGACGCTCATCCAGCAGAAATGAAAAATATCACCCGGCTAAAAA
O7K1xx29              CGCG-CCTTCTGGCGACGACGCTCATCCAGCAGAAATGAAAAATATCACCCGGCTAAAAA
SMS_3_5               CGCG-CCTTCTGGCGACGACGCTCATCCAGCAGAAATGAAAAATATCACCCGGCTAAAAA
RM12579               CGCGGCCTTCTGGCGACGACGCTCATCCAGCAGAAATGAAAAATATCACCCGGCTAAAAA
CB9615                CGCGGCCTTCTGGCGACGACGCTCATCCAGCAGAAATGAAAAATATCACCCGGCTAAAAA
Xuzhou21              CGCGGCCTTCTGGCGACGACGCTCATCCAGCAGAAATGAAAAATATCACCCGGCTAAAAA
TW14359               CGCGGCCTTCTGGCGACGACGCTCATCCAGCAGAAATGAAAAATATCACCCGGCTAAAAA
EC4115                CGCGGCCTTCTGGCGACGACGCTCATCCAGCAGAAATGAAAAATATCACCCGGCTAAAAA
EDL933                CGCGGCCTTCTGGCGACGACGCTCATCCAGCAGAAATGAAAAATATCACCCGGCTAAAAA
Sakaix28              CGCGGCCTTCTGGCGACGACGCTCATCCAGCAGAAATGAAAAATATCACCCGGCTAAAAA
                      ** * ************ **************************** ******* *****
Prim.cons.            CGTGGCCTTCTGGCGACGACGCTCATCCAGCAGAAATGAAAAATATCACCCGGCTAAAAA

                                130       140       150       160       170       180
                                  |         |         |         |         |         |
MG1655                ATAGAATAGAAGCATTCGCCATAATGACATTAAACATTGTTTGATATTTATCATATTAAT
MDS42x1               ATAGAATAGAAGCATTCGCCATAATGACATTAAACATTGTTTGATATTTATCATATTAAT
W3110x2               ATAGAATAGAAGCATTCGCCATAATGACATTAAACATTGTTTGATATTTATCATATTAAT
DH1NC_0176381         ATAGAATAGAAGCATTCGCCATAATGACATTAAACATTGTTTGATATTTATCATATTAAT
ETECxx4               ATAGAATAGAAGCATTCGCCATAATGACATTAAACATTGTTTGATATTTATCATATTAAT
P12bxx5               ATAGAATAGAAGCATTCGCCATAATGACATTAAACATTGTTTGATATTTATCATATTAAT
BW2952                ATAGAATAGAAGCATTCGCCATAATGACATTAAACATTGTTTGATATTTATCATATTAAT
DH10Bx7               ATAGAATAGAAGCATTCGCCATAATGACATTAAACATTGTTTGATATTTATCATATTAAT
C321deltaA            ATAGAATAGAAGCATTCGCCATAATGACATTAAACATTGTTTGATATTTATCATATTAAT
Wxxxxx20              ATAGAATAGAAGCATTCGCCATAATGACATTAAACATTGTTTGATATTTATCATATTAAT
KO11FL                ATAGAATAGAAGCATTCGCCATAATGACATTAAACATTGTTTGATATTTATCATATTAAT
LY180x19              ATAGAATAGAAGCATTCGCCATAATGACATTAAACATTGTTTGATATTTATCATATTAAT
E24377A               ATAGAATAGAAGCATTCGCCATAATGACATTAAACATTGTTTGATATTTATCATATTAAT
SE11xx14              ATAGAATAGAAGCATTCGCCATAATGACATTAAACATTGTTTGATATTTATCATATTAAT
2009EL_2050           ATAGAATAGAAGCATTCGCCATAATGACATTAAACATTGTTTGATATTTATCATATTAAT
2011C_3493            ATAGAATAGAAGCATTCGCCATAATGACATTAAACATTGTTTGATATTTATCATATTAAT
55989x18              ATAGAATAGAAGCATTCGCCATAATGACATTAAACATTGTTTGATATTTATCATATTAAT
IAI1xx13              ATAGAATAGAAGCATTCGCCATAATGACATTAAACATTGTTTGATATTTATCATATTAAT
O103H2                ATAGAATAGAAGCATTCGCCATAATGACATTAAACATTGTTTGATATTTATCATATTAAT
APECxx11              ATAGAATAGAAGCATTCGCCATAATGACATTAAACATTGTTTGATATTTATCATATTAAT
UMNK88                ATAGAATAGAAGCATTCGCCATAATGACATTAAACATTGTTTGATATTTATCATATTAAT
HSxxxx39              ATAGAATAGAAGCATTCGCCATAATGACATTAAACATTGTTTGATATTTATCATATTAAT
BL21DE3               ATAGAATAGAAGCATTCGCCATAATGACATTAAACATTGTTTGATATTTATCATATTAAT
REL606                ATAGAATAGAAGCATTCGCCATAATGACATTAAACATTGTTTGATATTTATCATATTAAT
BL21_GoldDE3pLysS     ATAGAATAGAAGCATTCGCCATAATGACATTAAACATTGTTTGATATTTATCATATTAAT
ATCCxx37              ATAGAATAGAAGCATTCGCCATAATGACATTAAACATTGTTTGATATTTATCATATTAAT
O111H_                ATAGAATAGAAGCATTCGCCATAATGACATTAAACATTGTTTGATATTTATCATATTAAT
O26H11                ATAGAATAGAAGCATTCGCCATAATGACATTAAACATTGTTTGATATTTATCATATTAAT
UMN026                -TAGAATAGAAGCATTCGCCATAATGACATTAAACATTGTTTGATATTTATCATATTAAT
042NC_0176261         ATAGAATAGAAGCATTCGCCATAATGACATTAAACATTGTTTGATATTTATCATATTAAT
IAI39x23              ATAGAATAGAAGCATCAGCCATAATGACATTAAACATTGTTTGATATTTATCATATTAAT
O7K1xx29              ATAGAATAGAAGCATCAGCCATAATGACATTAAACATTGTTTGATATTTATCATATTAAT
SMS_3_5               ATAGAATAGAAGCATCAGCCATAATGACATTAAACATTGTTTGATATTTATCATATTAAT
RM12579               ATAGAATAGAAGCATTCGCCATAATGACATTAAACATTGTTTGATATTTATCATATTAAT
CB9615                ATAGAATAGAAGCATTCGCCATAATGACATTAAACATTGTTTGATATTTATCATATTAAT
Xuzhou21              ATAGAATAGAAGCATTCGCCATAATGACATTAAACATTGTTTGATATTTATCATATTAAT
TW14359               ATAGAATAGAAGCATTCGCCATAATGACATTAAACATTGTTTGATATTTATCATATTAAT
EC4115                ATAGAATAGAAGCATTCGCCATAATGACATTAAACATTGTTTGATATTTATCATATTAAT
EDL933                ATAGAATAGAAGCATTCGCCATAATGACATTAAACATTGTTTGATATTTATCATATTAAT
Sakaix28              ATAGAATAGAAGCATTCGCCATAATGACATTAAACATTGTTTGATATTTATCATATTAAT
                       **************  *******************************************
Prim.cons.            ATAGAATAGAAGCATTCGCCATAATGACATTAAACATTGTTTGATATTTATCATATTAAT

                                190       200       210       220       230       240
```

```
                              |         |         |         |         |         |
MG1655                        AGAAATAAAGACATTGACGCATCCCGCCCGGCTAACTATGAATTAGATGAAGTAAAATTT
MDS42x1                       AGAAATAAAGACATTGACGCATCCCGCCCGGCTAACTATGAATTAGATGAAGTAAAATTT
W3110x2                       AGAAATAAAGACATTGACGCATCCCGCCCGGCTAACTATGAATTAGATGAAGTAAAATTT
DH1NC_0176381                 AGAAATAAAGACATTGACGCATCCCGCCCGGCTAACTATGAATTAGATGAAGTAAAATTT
ETECxx4                       AGAAATAAAGACATTGACGCATCCCGCCCGGCTAACTATGAATTAGATGAAGTAAAATTT
P12bxx5                       AGAAATAAAGACATTGACGCATCCCGCCCGGCTAACTATGAATTAGATGAAGTAAAATTT
BW2952                        AGAAATAAAGACATTGACGCATCCCGCCCGGCTAACTATGAATTAGATGAAGTAAAATTT
DH10Bx7                       AGAAATAAAGACATTGACGCATCCCGCCCGGCTAACTATGAATTAGATGAAGTAAAATTT
C321deltaA                    AGAAATAAAGACATTGACGCATCCCGCCCGGCTAACTATGAATTAGATGAAGTAAAATTT
Wxxxxx20                      AGAAATAAAGACATTGACGCATCCCGCCCGGCTAACTATGAATTAGATGAAGTAAAATTT
KO11FL                        AGAAATAAAGACATTGACGCATCCCGCCCGGCTAACTATGAATTAGATGAAGTAAAATTT
LY180x19                      AGAAATAAAGACATTGACGCATCCCGCCCGGCTAACTATGAATTAGATGAAGTAAAATTT
E24377A                       AGAAATAAAGACATTGACGCATCCCGCCCGGCTAACTATGAATTAGATGAAGTAAAATTT
SE11xx14                      AGAAATAAAGACATTGACGCATCCCGCCCGGCTAACTATGAATTAGATGAAGTAAAATTT
2009EL_2050                   AGAAATAAAGACATTGACGCATCCCGCCCGGCTAACTATGAATTAGATGAAGTAAAATTT
2011C_3493                    AGAAATAAAGACATTGACGCATCCCGCCCGGCTAACTATGAATTAGATGAAGTAAAATTT
55989x18                      AGAAATAAAGACATTGACGCATCCCGCCCGGCTAACTATGAATTAGATGAAGTAAAATTT
IAI1xx13                      AGAAATAAAGACATTGACGCATCCCGCCCGGCTAACTATGAATTAGATGAAGTAAAATTT
O103H2                        AGAAATAAAGACATTGACGCATCCCGCCCGGCTAACTATGAATTAGATGAAGTAAAATTT
APECxx11                      AGAAATAAAGACATTGACGCATCCCGCCCGGCTAACTATGAATTAGATGAAGTAAAATTT
UMNK88                        AGAAATAAAGACATTGACGCATCCCGCCCGGCTAACTATGAATTAGATGAAGTAAAATTT
HSxxxx39                      AGAAATAAAGACATTGACGCATCCCGCCCGGCTAACTATGAATTAGATGAAGTAAAATTT
BL21DE3                       AGAAATAAAGACATTGACGCATCCCGCCCGGCTAACTATGAATTAGATGAAGTAAAATTT
REL606                        AGAAATAAAGACATTGACGCATCCCGCCCGGCTAACTATGAATTAGATGAAGTAAAATTT
BL21_GoldDE3pLysS             AGAAATAAAGACATTGACGCATCCCGCCCGGCTAACTATGAATTAGATGAAGTAAAATTT
ATCCxx37                      AGAAATAAAGACATTGACGCATCCCGCCCGGCTAACTATGAATTAGATGAAGTAAAATTT
O111H_                        AGAAATAAAGACATTGACGCATCCCGCCCGGCTAACTATGAATTAGATGAAGTAAAATTT
O26H11                        AGAAATAAAGACATTGACGCATCCCGCCCGGCTAACTATGAATTAGATGAAGTAAAATTT
UMN026                        AGAAATAAAGACATTGACGCATCTCGCCCGGCTAACTATGAATTAGATGAAGTAAAATTC
042NC_0176261                 AGAAATAAAGACATTGACGCATCTCGCCCGGCTAACTATGAATTAGATGAAGTAAAATTC
IAI39x23                      AGAAATAAAGACATTGACGCATCCCGCCCGGCTAACTATGAATTAGATGAAGTAAAATTC
O7K1xx29                      AGAAATAAAGACATTGACGCATCCCGCCCGGCTAACTATGAATTAGATGAAGTAAAATTC
SMS_3_5                       AGAAATAAAGACATTGACGCATCCCGCCCGGCTAACTATGAATTAGATGATGTAAAATTC
RM12579                       AGAAATAAAGACATTGACGCATCTCTCCCGGCTAACTATGAATTAGATGAAGTAAAATTC
CB9615                        AGAAATAAAGACATTGACGCATCTCTCCCGGCTAACTATGAATTAGATGAAGTAAAATTC
Xuzhou21                      AGAAATAAAGACATTGACGCATCTCTCCCGGCTAACTATGAATTAGATGAAGTAAAATTC
TW14359                       AGAAATAAAGACATTGACGCATCTCTCCCGGCTAACTATGAATTAGATGAAGTAAAATTC
EC4115                        AGAAATAAAGACATTGACGCATCTCTCCCGGCTAACTATGAATTAGATGAAGTAAAATTC
EDL933                        AGAAATAAAGACATTGACGCATCTCTCCCGGCTAACTATGAATTAGATGAAGTAAAATTC
Sakaix28                      AGAAATAAAGACATTGACGCATCTCTCCCGGCTAACTATGAATTAGATGAAGTAAAATTC
                              *********************** * ************************ ******** 

Prim.cons.                    AGAAATAAAGACATTGACGCATCCCGCCCGGCTAACTATGAATTAGATGAAGTAAAATTT

                                       250       260       270       280       290       300
                                         |         |         |         |         |         |
MG1655                        ATTAATAGTTGTAAAACAGGAGTTTCATTACAATTTATATATTTAAAGAGGCGAATGATT
MDS42x1                       ATTAATAGTTGTAAAACAGGAGTTTCATTACAATTTATATATTTAAAGAGGCGAATGATT
W3110x2                       ATTAATAGTTGTAAAACAGGAGTTTCATTACAATTTATATATTTAAAGAGGCGAATGATT
DH1NC_0176381                 ATTAATAGTTGTAAAACAGGAGTTTCATTACAATTTATATATTTAAAGAGGCGAATGATT
ETECxx4                       ATTAATAGTTGTAAAACAGGAGTTTCATTACAATTTATATATTTAAAGAGGCGAATGATT
P12bxx5                       ATTAATAGTTGTAAAACAGGAGTTTCATTACAATTTATATATTTAAAGAGGCGAATGATT
BW2952                        ATTAATAGTTGTAAAACAGGAGTTTCATTACAATTTATATATTTAAAGAGGCGAATGATT
DH10Bx7                       ATTAATAGTTGTAAAACAGGAGTTTCATTACAATTTATATATTTAAAGAGGCGAATGATT
C321deltaA                    ATTAATAGTTGTAAAACAGGAGTTTCATTACAATTTATATATTTAAAGAGGCGAATGATT
Wxxxxx20                      ATTAATAGTTGTAAAACAGGAGTTTCATTACAATTTATATATTTAAAGAGGCGAATGATT
KO11FL                        ATTAATAGTTGTAAAACAGGAGTTTCATTACAATTTATATATTTAAAGAGGCGAATGATT
LY180x19                      ATTAATAGTTGTAAAACAGGAGTTTCATTACAATTTATATATTTAAAGAGGCGAATGATT
E24377A                       ATTAATAGTTGTAAAACAGGAGTTTCATTACAATTTATATATTTAAAGAGGCGAATGATT
SE11xx14                      ATTAATAGTTGTAAAACAGGAGTTTCATTACAATTTATATATTTAAAGAGGCGAATGATT
2009EL_2050                   ATTAATAGTTGTAAAACAGGAGTTTCATTACAATTTATATATTTAAAGAGGCGAATGATT
2011C_3493                    ATTAATAGTTGTAAAACAGGAGTTTCATTACAATTTATATATTTAAAGAGGCGAATGATT
55989x18                      ATTAATAGTTGTAAAACAGGAGTTTCATTACAATTTATATATTTAAAGAGGCGAATGATT
IAI1xx13                      ATTAATAGTTGTAAAACAGGAGTTTCATTACAATTTATATATTTAAAGAGGCGAATGATT
O103H2                        ATTAATAGTTGTAAAACAGGAGTTTCATTACAATTTATATATTTAAAGAGGCGAATGATT
APECxx11                      ATTAATAGTTGTAAAACAGGAGTTTCATTACAATTTATATATTTAAAGAGGCGAATGATT
UMNK88                        ATTAATAGTTGTAAAACAGGAGTTTCATTACAATTTATATATTTAAAGAGGCGAATGATT
HSxxxx39                      ATTAATAGTTGTAAAACAGGAGTTTCATTACAATTTATATATTTAAAGAGGCGAATGATT
```

```
BL21DE3              ATTAATAGTTGTAAAACAGGAGTTTCATTACAATTTATATATTTAAAGAGGCGAATGATT
REL606               ATTAATAGTTGTAAAACAGGAGTTTCATTACAATTTATATATTTAAAGAGGCGAATGATT
BL21_GoldDE3pLysS    ATTAATAGTTGTAAAACAGGAGTTTCATTACAATTTATATATTTAAAGAGGCGAATGATT
ATCCxx37             ATTAATAGTTGTAAAACAGGAGTTTCATTACAATTTATATATTTAAAGAGGCGAATGATT
O111H_               ATTAATAGTTGTAAAACAGGAGTTTCATTACAATTTATATATTTAAAGAGGCGAATGATT
O26H11               ATTAATAGTTGTAAAACAGGAGTTTCATTACAATTTATATATTTAAAGAGGCGAATGATT
UMN026               ATTAATAGTTGTAAAACAGGAGTTTCATTACAATTTATATATTTAAAGAGGCGAATGATT
042NC_0176261        ATTAATAGTTGTAAAACAGGAGTTTCATTACAATTTATATATTTAAAGAGGCGAATGATT
IAI39x23             TTTAATAGTTGTAAAACAGGAGTTTCATTACAATTTATATATTTAAAGAGGCGAATGATT
O7K1xx29             TTTAATAGTTGTAAAACAGGAGTTTCATTACAATTTATATATTTAAAGAGGCGAATGATT
SMS_3_5              TTTAATAGTTGTAAAACAGGAGTTTCATTACAATTTATATATTTAAAGAGGCGAATGATT
RM12579              ATTAATAGTTGTAAAACAGGAGTTTCATTACAATTTATATATTTAAAGAGGCGAATGATT
CB9615               ATTAATAGTTGTAAAACAGGAGTTTCATTACAATTTATATATTTAAAGAGGCGAATGATT
Xuzhou21             ATTAATAGTTGTAAAACAGGAGTTTCATTACAATTTATATATTTAAAGAGGCGAATGATT
TW14359              ATTAATAGTTGTAAAACAGGAGTTTCATTACAATTTATATATTTAAAGAGGCGAATGATT
EC4115               ATTAATAGTTGTAAAACAGGAGTTTCATTACAATTTATATATTTAAAGAGGCGAATGATT
EDL933               ATTAATAGTTGTAAAACAGGAGTTTCATTACAATTTATATATTTAAAGAGGCGAATGATT
Sakaix28             ATTAATAGTTGTAAAACAGGAGTTTCATTACAATTTATATATTTAAAGAGGCGAATGATT
                      ***********************************************************
Prim.cons.           ATTAATAGTTGTAAAACAGGAGTTTCATTACAATTTATATATTTAAAGAGGCGAATGATT

                            310       320       330       340       350       360
                              |         |         |         |         |         |
MG1655               ATGACTGAAATCGTTGCAGATAAAACGGTAGAAGTAGTTAAAAACGCAATCGAAACCGCA
MDS42x1              ATGACTGAAATCGTTGCAGATAAAACGGTAGAAGTAGTTAAAAACGCAATCGAAACCGCA
W3110x2              ATGACTGAAATCGTTGCAGATAAAACGGTAGAAGTAGTTAAAAACGCAATCGAAACCGCA
DH1NC_0176381        ATGACTGAAATCGTTGCAGATAAAACGGTAGAAGTAGTTAAAAACGCAATCGAAACCGCA
ETECxx4              ATGACTGAAATCGTTGCAGATAAAACGGTAGAAGTAGTTAAAAACGCAATCGAAACCGCA
P12bxx5              ATGACTGAAATCGTTGCAGATAAAACGGTAGAAGTAGTTAAAAACGCAATCGAAACCGCA
BW2952               ATGACTGAAATCGTTGCAGATAAAACGGTAGAAGTAGTTAAAAACGCAATCGAAACCGCA
DH10Bx7              ATGACTGAAATCGTTGCAGATAAAACGGTAGAAGTAGTTAAAAACGCAATCGAAACCGCA
C321deltaA           ATGACTGAAATCGTTGCAGATAAAACGGTAGAAGTAGTTAAAAACGCAATCGAAACCGCA
Wxxxxx20             ATGACTGAAATCGTTGCAGATAAAACGGTAGAAGTAGTTAAAAACGCAATCGAAACTGCA
KO11FL               ATGACTGAAATCGTTGCAGATAAAACGGTAGAAGTAGTTAAAAACGCAATCGAAACTGCA
LY180x19             ATGACTGAAATCGTTGCAGATAAAACGGTAGAAGTAGTTAAAAACGCAATCGAAACTGCA
E24377A              ATGACTGAAATCGTTGCAGATAAAACGGTAGAAGTAGTTAAAAACGCAATCGAAACTGCA
SE11xx14             ATGACTGAAATCGTTGCAGATAAAACGGTAGAAGTAGTTAAAAACGCAATCGAAACTGCA
2009EL_2050          ATGACTGAAATCGTTGCAGATAAAACGGTAGAAGTAGTTAAAAACGCAATCGAAACTGCA
2011C_3493           ATGACTGAAATCGTTGCAGATAAAACGGTAGAAGTAGTTAAAAACGCAATCGAAACTGCA
55989x18             ATGACTGAAATCGTTGCAGATAAAACGGTAGAAGTAGTTAAAAACGCAATCGAAACTGCA
IAI1xx13             ATGACTGAAATCGTTGCAGATAAAACGGTAGAAGTAGTTAAAAACGCAATCGAAACTGCA
O103H2               ATGACTGAAATCGTTGCAGATAAAACGGTAGAAGTAGTTAAAAACGCAATCGAAACTGCA
APECxx11             ATGACTGAAATCGTTGCAGATAAAACGGTAGAAGTAGTTAAAAACGCAATCGAAACTGCA
UMNK88               ATGACTGAAATCGTTGCAGATAAAACGGTAGAAGTAGTTAAAAACGCAATCGAAACCGCA
HSxxxx39             ATGACTGAAATCGTTGCAGATAAAACGGTAGAAGTAGTTAAAAACGCAATCGAAACCGCA
BL21DE3              ATGACTGAAATCGTTGCAGATAAAACGGTAGAAGTAGTTAAAAACGCAATCGAAACCGCA
REL606               ATGACTGAAATCGTTGCAGATAAAACGGTAGAAGTAGTTAAAAACGCAATCGAAACCGCA
BL21_GoldDE3pLysS    ATGACTGAAATCGTTGCAGATAAAACGGTAGAAGTAGTTAAAAACGCAATCGAAACCGCA
ATCCxx37             ATGACTGAAATCGTTGCAGATAAAACGGTAGAAGTAGTTAAAAACGCAATCGAAACCGCA
O111H_               ATGACTGAAATCGTTGCAGATAAAACGGTAGAAGTAGTTAAAAACGCAATCGAAACCGCA
O26H11               ATGACTGAAATCGTTGCAGATAAAACGGTAGAAGTAGTTAAAAACGCAATCGAAACCGCA
UMN026               ATGACTGAAATCGTTGCAGATAAAACGGTAGAGGTAGTTAAAAACGCAATCGAAACCGCA
042NC_0176261        ATGACTGAAATCGTTGCAGATAAAACGGTAGAGGTAGTTAAAAACGCAATCGAAACCGCA
IAI39x23             ATGACTGAAATCGTTGCAGATAAAACGGTAGAGGTAGTTAAAAACGCAATCGAAACCGCA
O7K1xx29             ATGACTGAAATCGTTGCAGATAAAACGGTAGAGGTAGTTAAAAACGCAATCGAAACCGCA
SMS_3_5              ATGACTGAAATCGTTGCAGATAAAACGGTAGAGGTAGTTAAAAACGCAATCGAAACCGCA
RM12579              ATGACTGAAATCGTTGCAGATAAAACGGTAGAGGTAGTTAAAAACGCAATCGAAACCGCA
CB9615               ATGACTGAAATCGTTGCAGATAAAACGGTAGAGGTAGTTAAAAACGCAATCGAAACCGCA
Xuzhou21             ATGACTGAAATCGTTGCAGATAAAACGGTAGAGGTAGTTAAAAACGCAATCGAAACCGCA
TW14359              ATGACTGAAATCGTTGCAGATAAAACGGTAGAGGTAGTTAAAAACGCAATCGAAACCGCA
EC4115               ATGACTGAAATCGTTGCAGATAAAACGGTAGAGGTAGTTAAAAACGCAATCGAAACCGCA
EDL933               ATGACTGAAATCGTTGCAGATAAAACGGTAGAGGTAGTTAAAAACGCAATCGAAACCGCA
Sakaix28             ATGACTGAAATCGTTGCAGATAAAACGGTAGAGGTAGTTAAAAACGCAATCGAAACCGCA
                     ******************************** *********************** ***
Prim.cons.           ATGACTGAAATCGTTGCAGATAAAACGGTAGAAGTAGTTAAAAACGCAATCGAAACCGCA

                            370       380       390       400       410       420
```

```
                                 |         |         |         |         |         |
MG1655                    GATGGAGCATTAGATCTTTATAATAAATATCTCGATCAGGTCATCCCCTGGCAGACCTTT
MDS42x1                   GATGGAGCATTAGATCTTTATAATAAATATCTCGATCAGGTCATCCCCTGGCAGACCTTT
W3110x2                   GATGGAGCATTAGATCTTTATAATAAATATCTCGATCAGGTCATCCCCTGGCAGACCTTT
DH1NC_0176381             GATGGAGCATTAGATCTTTATAATAAATATCTCGATCAGGTCATCCCCTGGCAGACCTTT
ETECxx4                   GATGGAGCATTAGATCTTTATAATAAATATCTCGATCAGGTCATCCCCTGGCAGACCTTT
P12bxx5                   GATGGAGCATTAGATCTTTATAATAAATATCTCGATCAGGTCATCCCCTGGCAGACCTTT
BW2952                    GATGGAGCATTAGATCTTTATAATAAATATCTCGATCAGGTCATCCCCTGGCAGACCTTT
DH10Bx7                   GATGGAGCATTAGATCTTTATAATAAATATCTCGATCAGGTCATCCCCTGGCAGACCTTT
C321deltaA                GATGGAGCATTAGATCTTTATAATAAATATCTCGATCAGGTCATCCCCTGGCAGACCTTT
Wxxxxx20                  GATGGAGCATTAGATCTTTATAATAAATATCTCGATCAGGTCATCCCCTGGCAGACCTTT
KO11FL                    GATGGAGCATTAGATCTTTATAATAAATATCTCGATCAGGTCATCCCCTGGCAGACCTTT
LY180x19                  GATGGAGCATTAGATCTTTATAATAAATATCTCGATCAGGTCATCCCCTGGCAGACCTTT
E24377A                   GATGGAGCATTAGATCTTTATAATAAATATCTCGATCAGGTCATCCCCTGGCAGACCTTT
SE11xx14                  GATGGAGCATTAGATCTTTATAATAAATATCTCGATCAGGTCATCCCCTGGCAGACCTTT
2009EL_2050               GATGGAGCATTAGATCTTTATAATAAATATCTCGATCAGGTCATCCCCTAGCAGACCTTT
2011C_3493                GATGGAGCATTAGATCTTTATAATAAATATCTCGATCAGGTCATCCCCTAGCAGACCTTT
55989x18                  GATGGAGCATTAGATCTTTATAATAAATATCTCGATCAGGTCATCCCCTAGCAGACCTTT
IAI1xx13                  GATGGAGCATTAGATCTTTATAATAAATATCTCGATCAGGTCATCCCCTGGCAGACCTTT
O103H2                    GATGGAGCATTAGATCTTTATAATAAATATCTCGATCAGGTCATCCCCTGGCAGACCTTT
APECxx11                  GATGGAGCATTAGATCTTTATAATAAATATCTCGATCAGGTCATCCCCTGGCAGACCTTT
UMNK88                    GATGGAGCATTAGATCTTTATAATAAATATCTCGATCAGGTCATCCCCTGGCAGACCTTT
HSxxxx39                  GATGGAGCATTAGATCTTTATAATAAATATCTCGATCAGGTCATCCCCTGGCAGACCTTT
BL21DE3                   GATGGAGCATTAGATCTTTATAATAAATATCTCGATCAGGTCATCCCCTGGCAGACCTTT
REL606                    GATGGAGCATTAGATCTTTATAATAAATATCTCGATCAGGTCATCCCCTGGCAGACCTTT
BL21_GoldDE3pLysS         GATGGAGCATTAGATCTTTATAATAAATATCTCGATCAGGTCATCCCCTGGCAGACCTTT
ATCCxx37                  GATGGAGCATTAGATCTTTATAATAAATATCTCGATCAGGTCATCCCCTGGCAGACCTTT
O111H_                    GATGGAGCATTAGATCTTTATAATAAATATCTCGATCAGGTCATCCCCTGGCAGACCTTT
O26H11                    GATGGAGCATTAGATCTTTATAATAAATATCTCGATCAGGTCATCCCCTGGCAGACCTTT
UMN026                    GATGGAGCATTAGACCTTTATAATAAATATCTCGATCAGGTCATCCCCTGGCAGACCTTC
042NC_0176261             GATGGAGCATTAGACCTTTATAATAAATATCTCGATCAGGTCATCCCCTGGCAGACCTTC
IAI39x23                  GATGGAGCATTAGATCTTTATAATAAATATCTCGATCAGGTCATCCCCTGGCAGACCTTT
O7K1xx29                  GATGGAGCATTAGATCTTTATAATAAATATCTCGATCAGGTCATCCCCTGGCAGACCTTT
SMS_3_5                   GATGGAGCATTAGATCTTTATAATAAATATCTCGATCAGGTCATCCCCTGGCAGACCTTT
RM12579                   GATGGAGCATTGGATCTTTATAATAAATATCTCGATCAGGTCATCCCCTGGCAGACCTTT
CB9615                    GATGGAGCATTGGATCTTTATAATAAATATCTCGATCAGGTCATCCCCTGGCAGACCTTT
Xuzhou21                  GATGGAGCATTGGATCTTTATAATAAATATCTCGATCAGGTCATCCCCTGGCAGACCTTT
TW14359                   GATGGAGCATTGGATCTTTATAATAAATATCTCGATCAGGTCATCCCCTGGCAGACCTTT
EC4115                    GATGGAGCATTGGATCTTTATAATAAATATCTCGATCAGGTCATCCCCTGGCAGACCTTT
EDL933                    GATGGAGCATTGGATCTTTATAATAAATATCTCGATCAGGTCATCCCCTGGCAGACCTTT
Sakaix28                  GATGGAGCATTGGATCTTTATAATAAATATCTCGATCAGGTCATCCCCTGGCAGACCTTT
                          *********** ** ********************************** *********

Prim.cons.                GATGGAGCATTAGATCTTTATAATAAATATCTCGATCAGGTCATCCCCTGGCAGACCTTT

                                430       440       450       460       470       480
                                 |         |         |         |         |         |
MG1655                    GATGAAACCATAAAAGAGTTAAGTCGCTTTAAACAGGAGTATTCACAGGCAGCCTCCGTT
MDS42x1                   GATGAAACCATAAAAGAGTTAAGTCGCTTTAAACAGGAGTATTCACAGGCAGCCTCCGTT
W3110x2                   GATGAAACCATAAAAGAGTTAAGTCGCTTTAAACAGGAGTATTCACAGGCAGCCTCCGTT
DH1NC_0176381             GATGAAACCATAAAAGAGTTAAGTCGCTTTAAACAGGAGTATTCACAGGCAGCCTCCGTT
ETECxx4                   GATGAAACCATAAAAGAGTTAAGTCGCTTTAAACAGGAGTATTCACAGGCAGCCTCCGTT
P12bxx5                   GATGAAACCATAAAAGAGTTAAGTCGCTTTAAACAGGAGTATTCACAGGCAGCCTCCGTT
BW2952                    GATGAAACCATAAAAGAGTTAAGTCGCTTTAAACAGGAGTATTCACAGGCAGCCTCCGTT
DH10Bx7                   GATGAAACCATAAAAGAGTTAAGTCGCTTTAAACAGGAGTATTCACAGGCAGCCTCCGTT
C321deltaA                GATGAAACCATAAAAGAGTTAAGTCGCTTTAAACAGGAGTATTCACAGGCAGCCTCCGTT
Wxxxxx20                  GATGAAACCATAAAAGAGTTAAGTCGCTTTAAACAGGAGTATTCACAGGCAGCCTCCGTT
KO11FL                    GATGAAACCATAAAAGAGTTAAGTCGCTTTAAACAGGAGTATTCACAGGCAGCCTCCGTT
LY180x19                  GATGAAACCATAAAAGAGTTAAGTCGCTTTAAACAGGAGTATTCACAGGCAGCCTCCGTT
E24377A                   GATGAAACCATAAAAGAGTTAAGTCGCTTTAAACAGGAGTATTCACAGGCAGCCTCCGTT
SE11xx14                  GATGAAACCATAAAAGAGTTAAGTCGCTTTAAACAGGAGTATTCACAGGCAGCCTCCGTT
2009EL_2050               GATGAAACCATAAAAGAGTTAAGTCGCTTTAAACAGGAGTATTCACAGGCAGCCTCCGTT
2011C_3493                GATGAAACCATAAAAGAGTTAAGTCGCTTTAAACAGGAGTATTCACAGGCAGCCTCCGTT
55989x18                  GATGAAACCATAAAAGAGTTAAGTCGCTTTAAACAGGAGTATTCACAGGCAGCCTCCGTT
IAI1xx13                  GATGAAACCATAAAAGAGTTAAGTCGCTTTAAACAGGAGTATTCACAGGCAGCCTCCGTT
O103H2                    GATGAAACCATAAAAGAGTTAAGTCGCTTTAAACAGGAGTATTCACAGGCAGCCTCCGTT
APECxx11                  GATGAAACCATAAAAGAGTTAAGTCGCTTTAAACAGGAGTATTCACAGGCAGCCTCCGTT
UMNK88                    GATGAAACCATAAAAGAGTTAAGTCGCTTTAAACAGGAGTATTCACAGGCAGCCTCCGTT
HSxxxx39                  GATGAAACCATAAAAGAGTTAAGTCGCTTTAAACAGGAGTATTAACAGGCAGCCTCCGTT
```

```
BL21DE3              GATGAAACCATAAAAGAGTTAAGTCGCTTTAAACAGGAGTATTCACAGGCAGCCTCCGTT
REL606               GATGAAACCATAAAAGAGTTAAGTCGCTTTAAACAGGAGTATTCACAGGCAGCCTCCGTT
BL21_GoldDE3pLysS    GATGAAACCATAAAAGAGTTAAGTCGCTTTAAACAGGAGTATTCACAGGCAGCCTCCGTT
ATCCxx37             GATGAAACCATAAAAGAGTTAAGTCGCTTTAAACAGGAGTATTCACAGGCAGCCTCCGTT
O111H_               GATGAAACCATAAAAGAGTTAAGTCGCTTTAAACAGGAGTATTCACAGGCAGCCTCCGTT
O26H11               GATGAAACCATAAAAGAGTTAAGTCGCTTTAAACAGGAGTATTCACAGGCAGCCTCCGTT
UMN026               GATGAAACCATAAAAGAGTTAAGTCGCTTTAAACAGGAGTATTCACAGGCAGCCTCCGTT
042NC_0176261        GATGAAACCATAAAAGAGTTAAGTCGCTTTAAACAGGAGTATTCACAGGCAGCCTCCGTT
IAI39x23             GATGAAACCATAAAAGAGTTAAGTCGCTTTAAACAGGAGTATTCACAGGCAGCCTCCGTT
O7K1xx29             GATGAAACCATAAAAGAGTTAAGTCGCTTTAAACAGGAGTATTCACAGGCAGCCTCCGTT
SMS_3_5              GATGAAACCATAAAAGAGTTAAGTCGCTTTAAACAGGAGTATTCACAGGCAGCCTCCGTT
RM12579              GATGAAACCATAAAAGAGTTAAGTCGCTTTAAACAGGAGTATTCACAGGCAGCCTCCGTT
CB9615               GATGAAACCATAAAAGAGTTAAGTCGCTTTAAACAGGAGTATTCACAGGCAGCCTCCGTT
Xuzhou21             GATGAAACCATAAAAGAGTTAAGTCGCTTTAAACAGGAGTATTCACAGGCAGCCTCCGTT
TW14359              GATGAAACCATAAAAGAGTTAAGTCGCTTTAAACAGGAGTATTCACAGGCAGCCTCCGTT
EC4115               GATGAAACCATAAAAGAGTTAAGTCGCTTTAAACAGGAGTATTCACAGGCAGCCTCCGTT
EDL933               GATGAAACCATAAAAGAGTTAAGTCGCTTTAAACAGGAGTATTCACAGGCAGCCTCCGTT
Sakaix28             GATGAAACCATAAAAGAGTTAAGTCGCTTTAAACAGGAGTATTCACAGGCAGCCTCCGTT
                     ******************************************* ****************

Prim.cons.           GATGAAACCATAAAAGAGTTAAGTCGCTTTAAACAGGAGTATTCACAGGCAGCCTCCGTT

                                    490       500       510       520       530       540
                                      |         |         |         |         |         |
MG1655               TTAGTCGGCGATATTAAAACCTTACTTATGGATAGCCAGGATAAGTATTTTGAAGCAACC
MDS42x1              TTAGTCGGCGATATTAAAACCTTACTTATGGATAGCCAGGATAAGTATTTTGAAGCAACC
W3110x2              TTAGTCGGCGATATTAAAACCTTACTTATGGATAGCCAGGATAAGTATTTTGAAGCAACC
DH1NC_0176381        TTAGTCGGCGATATTAAAACCTTACTTATGGATAGCCAGGATAAGTATTTTGAAGCAACC
ETECxx4              TTAGTCGGCGATATTAAAACCTTACTTATGGATAGCCAGGATAAGTATTTTGAAGCAACC
P12bxx5              TTAGTCGGCGATATTAAAACCTTACTTATGGATAGCCAGGATAAGTATTTTGAAGCAACC
BW2952               TTAGTCGGCGATATTAAAACCTTACTTATGGATAGCCAGGATAAGTATTTTGAAGCAACC
DH10Bx7              TTAGTCGGCGATATTAAAACCTTACTTATGGATAGCCAGGATAAGTATTTTGAAGCAACC
C321deltaA           TTAGTCGGCGATATTAAAACCTTACTTATGGATAGCCAGGATAAGTATTTTGAAGCAACC
Wxxxxx20             TTAGTCGGCGATATTAAAACCTTACTTATGGATAGC-AGGATAAGTATTTTGAAGCAACC
KO11FL               TTAGTCGGCGATATTAAAACCTTACTTATGGATAGC-AGGATAAGTATTTTGAAGCAACC
LY180x19             TTAGTCGGCGATATTAAAACCTTACTTATGGATAGC-AGGATAAGTATTTTGAAGCAACC
E24377A              TTAGTCGGCGATATTAAAACCTTACTTATGGATAGCCAGGATAAGTATTTTGAAGCAACC
SE11xx14             TTAGTCGGCGATATTAAAACCTTACTTATGGATAGCCAGGATAAGTATTTTGAAGCAACC
2009EL_2050          TTAGTCGGCGATATTAAAACCTTACTTATGGATAGCCAGGATAAGTATTTTGAAGCAACC
2011C_3493           TTAGTCGGCGATATTAAAACCTTACTTATGGATAGCCAGGATAAGTATTTTGAAGCAACC
55989x18             TTAGTCGGCGATATTAAAACCTTACTTATGGATAGCCAGGATAAGTATTTTGAAGCAACC
IAI1xx13             TTAGTCGGCGATATTAAAACCTTACTTATGGATAGCCAGGATAAGTATTTTGAAGCAACC
O103H2               TTAGTCGGCGATATTAAAACCTTACTTATGGATAGCCAGGATAAGTATTTTGAAGCAACC
APECxx11             TTAGTCGGCGATATTAAAACCTTACTTATGGATAGCCAGGATAAGTATTTTGAAGCAACC
UMNK88               TTAGTCGGCGATATTAAAACCTTACTTATGGATAGCCAGGATAAGTATTTTGAAGCAACC
HSxxxx39             TTAGTCGGCGATATTAAAACCTTACTTATGGATAGCCAGGATAAGTATTTTGAAGCAACC
BL21DE3              TTAGTCGGCGATATTAAAACCTTACTTATGGATAGCCAGGATAAGTATTTTGAAGCAACC
REL606               TTAGTCGGCGATATTAAAACCTTACTTATGGATAGCCAGGATAAGTATTTTGAAGCAACC
BL21_GoldDE3pLysS    TTAGTCGGCGATATTAAAACCTTACTTATGGATAGCCAGGATAAGTATTTTGAAGCAACC
ATCCxx37             TTAGTCGGCGATATTAAAACCTTACTTATGGATAGCCAGGATAAGTATTTTGAAGCAACC
O111H_               TTAGTCGGCGATATTAAAACCTTACTTATGGATAGCCAGGATAAGTATTTTGAAGCAACC
O26H11               TTAGTCGGCGATATTAAAACCTTACTTATGGATAGCCAGGATAAGTATTTTGAAGCAACC
UMN026               TTAGTTGGCGATATTAAAACCTTACTTATGGATAGCCAGGATAAGTATTTTGAAGCAACC
042NC_0176261        TTAGTTGGCGATATTAAAACCTTACTTATGGATAGCCAGGATAAGTATTTTGAAGCAACC
IAI39x23             TTAGTTGGCGATATTAAAACCTTACTTATGGATAGCCAGGATAAGTATTTTGAAGCAACC
O7K1xx29             TTAGTTGGCGATATTAAAACCTTACTTATGGATAGCCAGGATAAGTATTTTGAAGCAACC
SMS_3_5              TTAGTTGGCGATATTAAAACCTTACTTATGGATAGCCAGGATAAGTATTTTGAAGCAACC
RM12579              TTAGTTGGCAATATTAAAACCTTACTAATGGATAGCCAGGATAAGTATTTTGAAGCAACC
CB9615               TTAGTTGGCAATATTAAAACCTTACTAATGGATAGCCAGGATAAGTATTTTGAAGCAACC
Xuzhou21             TTAGTTGGCAATATTAAAACCTTACTAATGGATAGCCAGGATAAGTATTTTGAAGCAACC
TW14359              TTAGTTGGCAATATTAAAACCTTACTAATGGATAGCCAGGATAAGTATTTTGAAGCAACC
EC4115               TTAGTTGGCAATATTAAAACCTTACTAATGGATAGCCAGGATAAGTATTTTGAAGCAACC
EDL933               TTAGTTGGCAATATTAAAACCTTACTAATGGATAGCCAGGATAAGTATTTTGAAGCAACC
Sakaix28             TTAGTTGGCAATATTAAAACCTTACTAATGGATAGCCAGGATAAGTATTTTGAAGCAACC
                     ***** *** **************** ********* ***********************

Prim.cons.           TTAGTCGGCGATATTAAAACCTTACTTATGGATAGCCAGGATAAGTATTTTGAAGCAACC
```

```
                                     550       560       570       580       590       600
                                       |         |         |         |         |         |
MG1655                     CAAACAGTGTATGAATGGTGTGGTGTTGCGACGCAATTGCTCGCAGCGTATATTTTGCTA
MDS42x1                    CAAACAGTGTATGAATGGTGTGGTGTTGCGACGCAATTGCTCGCAGCGTATATTTTGCTA
W3110x2                    CAAACAGTGTATGAATGGTGTGGTGTTGCGACGCAATTGCTCGCAGCGTATATTTTGCTA
DH1NC_0176381              CAAACAGTGTATGAATGGTGTGGTGTTGCGACGCAATTGCTCGCAGCGTATATTTTGCTA
ETECxx4                    CAAACAGTGTATGAATGGTGTGGTGTTGCGACGCAATTGCTCGCAGCGTATATTTTGCTA
P12bxx5                    CAAACAGTGTATGAATGGTGTGGTGTTGCGACGCAATTGCTCGCAGCGTATATTTTGCTA
BW2952                     CAAACAGTGTATGAATGGTGTGGTGTTGCGACGCAATTGCTCGCAGCGTATATTTTGCTA
DH10Bx7                    CAAACAGTGTATGAATGGTGTGGTGTTGCGACGCAATTGCTCGCAGCGTATATTTTGCTA
C321deltaA                 CAAACAGTGTATGAATGGTGTGGTGTTGCGACGCAATTGCTCGCAGCGTATATTTTGCTA
Wxxxxx20                   CAAACAGTGTATGAATGGTGTGGTGTTGCGACGCAATTGCTCGCAGCGTATATTTTTCTA
KO11FL                     CAAACAGTGTATGAATGGTGTGGTGTTGCGACGCAATTGCTCGCAGCGTATATTTTTCTA
LY180x19                   CAAACAGTGTATGAATGGTGTGGTGTTGCGACGCAATTGCTCGCAGCGTATATTTTTCTA
E24377A                    CAAACAGTGTATGAATGGTGTGGTGTTGCGACGCAATTGCTCGCAGCGTATATTTTTCTA
SE11xx14                   CAAACAGTGTATGAATGGTGTGGTGTTGCGACGCAATTGCTCGCAGCGTATATTTTTCTA
2009EL_2050                CAAACAGTGTATGAATGGTGTGGTGTTGCGACGCAATTGCTCGCAGCGTATATTTTTCTA
2011C_3493                 CAAACAGTGTATGAATGGTGTGGTGTTGCGACGCAATTGCTCGCAGCGTATATTTTTCTA
55989x18                   CAAACAGTGTATGAATGGTGTGGTGTTGCGACGCAATTGCTCGCAGCGTATATTTTTCTA
IAI1xx13                   CAAACAGTGTATGAATGGTGTGGTGTTGCGACGCAATTGCTCGCAGCGTATATTTTTCTA
O103H2                     CAAACAGTGTATGAATGGTGTGGTGTTGCGACGCAATTGCTCGCAGCGTATATTTTTCTA
APECxx11                   CAAACAGTGTATGAATGGTGTGGTGTTGCGACGCAATTGCTCGCAGCGTATATTTTTCTA
UMNK88                     CAAACAGTGTATGAATGGTTTGGTGTTGCGACGCAATTGCTCGCAGCGTATATTTTGCTA
HSxxxx39                   CAAACAGTGTATGAATGGTGTGGTGTTGCGACGCAATTGCTCGCAGCGTATATTTTGCTA
BL21DE3                    CAAACAGTGTATGAATGGTGTGGTGTTGCGACGCAATTGCTCGCAGCGTATATTTTGCTA
REL606                     CAAACAGTGTATGAATGGTGTGGTGTTGCGACGCAATTGCTCGCAGCGTATATTTTGCTA
BL21_GoldDE3pLysS          CAAACAGTGTATGAATGGTGTGGTGTTGCGACGCAATTGCTCGCAGCGTATATTTTGCTA
ATCCxx37                   CAAACAGTGTATGAATGGTGTGGTGTTGCGACGCAATTGCTCGCAGCGTATATTTTGCTA
O111H_                     CAAACAGTGTATGAATGGTGTGGTGTTGCGACGCAATTGCTCGCAGCGTATATTTTGCTA
O26H11                     CAAACAGTGTATGAATGGTGTGGTGTTGCGACGCAATTGCTCGCAGCGTATATTTTGCTA
UMN026                     CAAACGGTGTATGAATGGTGTGGTGTTGCGACGCAATTGCTCGCAGCGTATATTTTGCTA
042NC_0176261              CAAACGGTGTATGAATGGTGTGGTGTTGCGACACAATTGCTCGCAGCATATATTTTGCTA
IAI39x23                   CAAACAGTGTATGAATGGTGTGGTGTTGCGACGCAATTGCTCGCAGCGTATATTTTGCTA
O7K1xx29                   CAAACAGTGTATGAATGGTGTGGTGTTGCGACGCAATTGCTCGCAGCGTATATTTTGCTA
SMS_3_5                    CAAACAGTGTATGAATGGTGTGGTGTTGCGACGCAATTGCTCGCAGCGTATATTTTGCTA
RM12579                    CAAACAGTGTATGAATGGTGTGGTGTTGCGACGCAATTGCTCGCAGCGTATATTTTGCTA
CB9615                     CAAACAGTGTATGAATGGTGTGGTGTTGCGACGCAATTGCTCGCAGCGTATATTTTGCTA
Xuzhou21                   CAAACAGTGTATGAATGGTGTGGTGTTGCGACGCAATTGCTCGCAGCGTATATTTTGCTA
TW14359                    CAAACAGTGTATGAATGGTGTGGTGTTGCGACGCAATTGCTCGCAGCGTATATTTTGCTA
EC4115                     CAAACAGTGTATGAATGGTGTGGTGTTGCGACGCAATTGCTCGCAGCGTATATTTTGCTA
EDL933                     CAAACAGTGTATGAATGGTGTGGTGTTGCGACGCAATTGCTCGCAGCGTATATTTTGCTA
Sakaix28                   CAAACAGTGTATGAATGGTGTGGTGTTGCGACGCAATTGCTCGCAGCGTATATTTTGCTA
                           ***** ************* ************ ************** ******** ***

Prim.cons.                 CAAACAGTGTATGAATGGTGTGGTGTTGCGACGCAATTGCTCGCAGCGTATATTTTGCTA

                                     610       620       630       640       650       660
                                       |         |         |         |         |         |
MG1655                     TTTGATGAGTACAATGAGAAGAAAGCATCCGCCCAGAAAGACATTCTCATTAAGGTACTG
MDS42x1                    TTTGATGAGTACAATGAGAAGAAAGCATCCGCCCAGAAAGACATTCTCATTAAGGTACTG
W3110x2                    TTTGATGAGTACAATGAGAAGAAAGCATCCGCCCAGAAAGACATTCTCATTAAGGTACTG
DH1NC_0176381              TTTGATGAGTACAATGAGAAGAAAGCATCCGCCCAGAAAGACATTCTCATTAAGGTACTG
ETECxx4                    TTTGATGAGTACAATGAGAAGAAAGCATCCGCCCAGAAAGACATTCTCATTAAGGTACTG
P12bxx5                    TTTGATGAGTACAATGAGAAGAAAGCATCCGCCCAGAAAGACATTCTCATTAAGGTACTG
BW2952                     TTTGATGAGTACAATGAGAAGAAAGCATCCGCCCAGAAAGACATTCTCATTAAGGTACTG
DH10Bx7                    TTTGATGAGTACAATGAGAAGAAAGCATCCGCCCAGAAAGACATTCTCATTAAGGTACTG
C321deltaA                 TTTGATGAGTACAATGAGAAGAAAGCATCCGCCCAGAAAGACATTCTCATTAAGGTACTG
Wxxxxx20                   TTTGATGAGTACAATGAGAAGAAAGCATCCGCCCAGAAAGACATTCTCATTAAGGTACTG
KO11FL                     TTTGATGAGTACAATGAGAAGAAAGCATCCGCCCAGAAAGACATTCTCATTAAGGTACTG
LY180x19                   TTTGATGAGTACAATGAGAAGAAAGCATCCGCCCAGAAAGACATTCTCATTAAGGTACTG
E24377A                    TTTGATGAGTACAATGAGAAGAAAGCATCCGCCCAGAAAGACATTCTCATTAAGGTACTG
SE11xx14                   TTTGATGAGTACAATGAGAAGAAAGCATCCGCCCAGAAAGACATTCTCATTAAGGTACTG
2009EL_2050                TTTGATGAGTACAATGAGAAGAAAGCATCCGCCCAGAAAGACATTCTCATTAAGGTACTG
2011C_3493                 TTTGATGAGTACAATGAGAAGAAAGCATCCGCCCAGAAAGACATTCTCATTAAGGTACTG
55989x18                   TTTGATGAGTACAATGAGAAGAAAGCATCCGCCCAGAAAGACATTCTCATTAAGGTACTG
IAI1xx13                   TTTGATGAGTACAATGAGAAGAAAGCATCCGCCCAGAAAGACATTCTCATTAAGGTACTG
O103H2                     TTTGATGAGTACAATGAGAAGAAAGCATCCGCCCAGAAAGACATTCTCATTAAGGTACTG
APECxx11                   TTTGATGAGTACAATGAGAAGAAAGCATCCGCCCAGAAAGACATTCTCATTAAGGTACTG
UMNK88                     TTTGATGAGTACAATGAGAAGAAAGCATCCGCCCAGAAAGACATTCTCATTAAGGTACTG
```

```
HSxxxx39              TTTGATGAGTACAATGAGAAGAAAGCATCCGCCCAGAAAGACATTCTCATTAAGGTACTG
BL21DE3               TTTGATGAGTACAATGAGAAGAAAGCATCCGCCCAGAAAGACATTCTCATTAAGGTACTG
REL606                TTTGATGAGTACAATGAGAAGAAAGCATCCGCCCAGAAAGACATTCTCATTAAGGTACTG
BL21_GoldDE3pLysS     TTTGATGAGTACAATGAGAAGAAAGCATCCGCCCAGAAAGACATTCTCATTAAGGTACTG
ATCCxx37              TTTGATGAGTACAATGAGAAGAAAGCATCCGCCCAGAAAGACATTCTCATTAAGGTACTG
O111H_                TTTGATGAGTACAATGAGAAGAAAGCATCCGCCCAGAAAGACATTCTCATTAAGGTACTG
O26H11                TTTGATGAGTACAATGAGAAGAAAGCATCCGCCCAGAAAGACATTCTCATTAAGGTACTG
UMN026                TTTGATGAGTACAATGAGAAGAAAGCATCCGCCCAGAAAGACATTCTCATTAAGGTACTG
042NC_0176261         TTTGATGAGTACAATGAGAAGAAAGCATCCGCCCAGAAAGACATTCTCATTAAGGTACTG
IAI39x23              TTTGATGAGTACAATGAGAAGAAAGCATCCGCCCAGAAAGACATTCTCATTAAGGTACTG
O7K1xx29              TTTGATGAGTACAATGAGAAGAAAGCATCCGCCCAGAAAGACATTCTCATTAAGGTACTG
SMS_3_5               TTTGATGAGTACAATGAGAAGAAAGCATCCGCCCAGAAAGACATTCTCATTAAGGTACTG
RM12579               TTTGATGAGTATAATGAGAAGAAAGCATCCGCCCAGAAAGACATTCTCATTAAGTTACTG
CB9615                TTTGATGAGTATAATGAGAAGAAAGCATCCGCCCAGAAAGACATTCTCATTAAGTTACTG
Xuzhou21              TTTGATGAGTATAATGAGAAGAAAGCATCCGCCCAGAAAGACATTCTCATTAAGGTACTG
TW14359               TTTGATGAGTATAATGAGAAGAAAGCATCCGCCCAGAAAGACATTCTCATTAAGGTACTG
EC4115                TTTGATGAGTATAATGAGAAGAAAGCATCCGCCCAGAAAGACATTCTCATTAAGGTACTG
EDL933                TTTGATGAGTATAATGAGAAGAAAGCATCCGCCCAGAAAGACATTCTCATTAAGGTACTG
Sakaix28              TTTGATGAGTATAATGAGAAGAAAGCATCCGCCCAGAAAGACATTCTCATTAAGGTACTG
                      *********** ****************************************** *****
Prim.cons.            TTTGATGAGTACAATGAGAAGAAAGCATCCGCCCAGAAAGACATTCTCATTAAGGTACTG

                             670       680       690       700       710       720
                               |         |         |         |         |         |
MG1655                GATGACGGCATCACGAAGCTGAATGAAGCGCAAAAATCCCTGCTGGTAAGCTCACAAAGT
MDS42x1               GATGACGGCATCACGAAGCTGAATGAAGCGCAAAAATCCCTGCTGGTAAGCTCACAAAGT
W3110x2               GATGACGGCATCACGAAGCTGAATGAAGCGCAAAAATCCCTGCTGGTAAGCTCACAAAGT
DH1NC_0176381         GATGACGGCATCACGAAGCTGAATGAAGCGCAAAAATCCCTGCTGGTAAGCTCACAAAGT
ETECxx4               GATGACGGCATCACGAAGCTGAATGAAGCGCAAAAATCCCTGCTGGTAAGCTCACAAAGT
P12bxx5               GATGACGGCATCACGAAGCTGAATGAAGCGCAAAAATCCCTGCTGGTAAGCTCACAAAGT
BW2952                GATGACGGCATCACGAAGCTGAATGAAGCGCAAAAATCCCTGCTGGTAAGCTCACAAAGT
DH10Bx7               GATGACGGCATCACGAAGCTGAATGAAGCGCAAAAATCCCTGCTGGTAAGCTCACAAAGT
C321deltaA            GATGACGGCATCACGAAGCTGAATGAAGCGCAAAAATCCCTGCTGGTAAGCTCACAAAGT
Wxxxxx20              GATGACGGCATCACGAAGCTGAATGAAGCGCAAAAATCCCTGCTGGTAAGCTCACAAAGT
KO11FL                GATGACGGCATCACGAAGCTGAATGAAGCGCAAAAATCCCTGCTGGTAAGCTCACAAAGT
LY180x19              GATGACGGCATCACGAAGCTGAATGAAGCGCAAAAATCCCTGCTGGTAAGCTCACAAAGT
E24377A               GATGACGGCATCACGAAGCTGAATGAAGCGCAAAAATCCCTGCTGGTAAGCTCACAAAGT
SE11xx14              GATGACGGCATCACGAAGCTGAATGAAGCGCAAAAATCCCTGCTGGTAAGCTCACAAAGT
2009EL_2050           GATGACGGCATCACGAAGCTGAATGAAGCGCAAAAATCCCTGCTGGTAAGCTCACAAAGT
2011C_3493            GATGACGGCATCACGAAGCTGAATGAAGCGCAAAAATCCCTGCTGGTAAGCTCACAAAGT
55989x18              GATGACGGCATCACGAAGCTGAATGAAGCGCAAAAATCCCTGCTGGTAAGCTCACAAAGT
IAI1xx13              GATGACGGCATCACGAAGCTGAATGAAGCGCAAAAATCCCTGCTGGTAAGCTCACAAAGT
O103H2                GATGACGGCATCACGAAGCTGAATGAAGCGCAAAAATCCCTGCTGGTAAGCTCACAAAGT
APECxx11              GATGACGGCATCACGAAGCTGAATGAAGCGCAAAAATCCCTGCTGGTAAGCTCACAAAGT
UMNK88                GATGACGGCATCACGAAGCTGAATGAAGCGCAAAAATCCCTGCTGGTAAGCTCACAAAGT
HSxxxx39              GATGACGGCATCACGAAGCTGAATGAAGCGCAAAAATCCCTGCTGGTAAGCTCACAAAGT
BL21DE3               GATGACGGCATCACGAAGCTGAATGAAGCGCAAAAATCCCTGCTGGTAAGCTCACAAAGT
REL606                GATGACGGCATCACGAAGCTGAATGAAGCGCAAAAATCCCTGCTGGTAAGCTCACAAAGT
BL21_GoldDE3pLysS     GATGACGGCATCACGAAGCTGAATGAAGCGCAAAAATCCCTGCTGGTAAGCTCACAAAGT
ATCCxx37              GATGACGGCATCACGAAGCTGAATGAAGCGCAAAAATCCCTGCTGGTAAGCTCACAAAGT
O111H_                GATGACGGCATCACGAAGCTGAATGAAGCGCAAAAATCCCTGCTGGTAAGCTCACAAAGT
O26H11                GATGACGGCATCACGAAGCTGAATGAAGCGCAAAAATCCCTGCTGGTAAGCTCACAAAGT
UMN026                GATGACGGCATCACGAAGCTGAATGAAGCGCAAAAATCCCTGCTGGTAAGCTCACAAAGT
042NC_0176261         GATGACGGCATCACGAAGCTGAATGAAGCGCAAAAATCTCTGCTGGTAAGCTCACAAAGT
IAI39x23              GATGACGGTATCACGAAGCTGAATGAAGCGCAAAAATCCCTGCTGGTAAGCTCACAAAGT
O7K1xx29              GATGACGGTATCACGAAGCTGAATGAAGCGCAAAAATCCCTGCTGGTAAGCTCACAAAGT
SMS_3_5               GATGACGGTATCACGAAGCTGAATGAAGCGCAAAAATCCCTGCTGGTAAGCTCACAAAGT
RM12579               GATGACGGCATCACGAAGCTGAATGAAGCGCAAAAATCCCTGCTGGTAAGCTCACAAAGT
CB9615                GATGACGGCATCACGAAGCTGAATGAAGCGCAAAAATCCCTGCTGGTAAGCTCACAAAGT
Xuzhou21              GATGACGGCATCACGAAGCTGAATGAAGCGCAAAAATCCCTGCTGGTAAGCTCACAAAGT
TW14359               GATGACGGCATCACGAAGCTGAATGAAGCGCAAAAATCCCTGCTGGTAAGCTCACAAAGT
EC4115                GATGACGGCATCACGAAGCTGAATGAAGCGCAAAAATCCCTGCTGGTAAGCTCACAAAGT
EDL933                GATGACGGCATCACGAAGCTGAATGAAGCGCAAAAATCCCTGCTGGTAAGCTCACAAAGT
Sakaix28              GATGACGGCATCACGAAGCTGAATGAAGCGCAAAAATCCCTGCTGGTAAGCTCACAAAGT
                      ******** ***************************** *********************
Prim.cons.            GATGACGGCATCACGAAGCTGAATGAAGCGCAAAAATCCCTGCTGGTAAGCTCACAAAGT
```

```
                                    730       740       750       760       770       780
                                      |         |         |         |         |         |
MG1655                    TTCAACAACGCTTCCGGGAAACTGCTGGCGTTAGATAGCCAGTTAACCAATGATTTTTCA
MDS42x1                   TTCAACAACGCTTCCGGGAAACTGCTGGCGTTAGATAGCCAGTTAACCAATGATTTTTCA
W3110x2                   TTCAACAACGCTTCCGGGAAACTGCTGGCGTTAGATAGCCAGTTAACCAATGATTTTTCA
DH1NC_0176381             TTCAACAACGCTTCCGGGAAACTGCTGGCGTTAGATAGCCAGTTAACCAATGATTTTTCA
ETECxx4                   TTCAACAACGCTTCCGGGAAACTGCTGGCGTTAGATAGCCAGTTAACCAATGATTTTTCA
P12bxx5                   TTCAACAACGCTTCCGGGAAACTGCTGGCGTTAGATAGCCAGTTAACCAATGATTTTTCA
BW2952                    TTCAACAACGCTTCCGGGAAACTGCTGGCGTTAGATAGCCAGTTAACCAATGATTTTTCA
DH10Bx7                   TTCAACAACGCTTCCGGGAAACTGCTGGCGTTAGATAGCCAGTTAACCAATGATTTTTCA
C321deltaA                TTCAACAACGCTTCCGGGAAACTGCTGGCGTTAGATAGCCAGTTAACCAATGATTTTTCA
Wxxxxx20                  TTCAACAACGCTTCCGGGAAACTGCTGGCGTTAGATAGCCAGTTAACCAATGATTTTTCA
KO11FL                    TTCAACAACGCTTCCGGGAAACTGCTGGCGTTAGATAGCCAGTTAACCAATGATTTTTCA
LY180x19                  TTCAACAACGCTTCCGGGAAACTGCTGGCGTTAGATAGCCAGTTAACCAATGATTTTTCA
E24377A                   TTCAACAACGCTTCCGGGAAACTGCTGGCGTTAGATAGCCAGTTAACCAATGATTTTTCA
SE11xx14                  TTCAACAACGCTTCCGGGAAACTGCTGGCGTTAGATAGCCAGTTAACCAATGATTTTTCA
2009EL_2050               TTCAACAACGCTTCCGGGAAACTGCTGGCGTTAGATAGCCAGTTAACCAATGATTTTTCA
2011C_3493                TTCAACAACGCTTCCGGGAAACTGCTGGCGTTAGATAGCCAGTTAACCAATGATTTTTCA
55989x18                  TTCAACAACGCTTCCGGGAAACTGCTGGCGTTAGATAGCCAGTTAACCAATGATTTTTCA
IAI1xx13                  TTCAACAACGCTTCCGGGAAACTGCTGGCGTTAGATAGCCAGTTAACCAATGATTTTTCA
O103H2                    TTCAACAACGCTTCCGGGAAACTGCTGGCGTTAGATAGCCAGTTAACCAATGATTTTTCA
APECxx11                  TTCAACAACGCTTCCGGGAAACTGCTGGCGTTAGATAGCCAGTTAACCAATGATTTTTCA
UMNK88                    TTCAACAACGCTTCCGGGAAACTGCTGGCGTTAGATAGCCAGTTAACCAATGATTTTTCA
HSxxxx39                  TTCAACAACGCTTCCGGGAAACTGCTGGCGTTAGATAGCCAGTTAACCAATGATTTTTCA
BL21DE3                   TTCAACAACGCTTCCGGGAAACTGCTGGCGTTAGATAGCCAGTTAACCAATGATTTTTCA
REL606                    TTCAACAACGCTTCCGGGAAACTGCTGGCGTTAGATAGCCAGTTAACCAATGATTTTTCA
BL21_GoldDE3pLysS         TTCAACAACGCTTCCGGGAAACTGCTGGCGTTAGATAGCCAGTTAACCAATGATTTTTCA
ATCCxx37                  TTCAACAACGCTTCCGGGAAACTGCTGGCGTTAGATAGCCAGTTAACCAATGATTTTTCA
O111H_                    TTCAACAACGCTTCCGGGAAACTGCTGGCGTTAGATAGCCAGTTAACCAATGATTTTTCA
O26H11                    TTCAACAACGCTTCCGGGAAACTGCTGGCGTTAGATAGCCAGTTAACCAATGATTTTTCA
UMN026                    TTCAACAACGCTTCCGGGAAACTGCTGGCGTTAGATAGCCAGTTAACCAATGATTTTTCA
042NC_0176261             TTCAACAACGCTTCCGGAAAACTGCTGGCGTTAGATAGCCAGTTAACCAATGATTTTTCA
IAI39x23                  TTCAACAACGCTTCCGGGAAACTGCTGGCGTTAGATAGCCAGTTAACCAATGATTTTTCA
O7K1xx29                  TTCAACAACGCTTCCGGGAAACTGCTGGCGTTAGATAGCCAGTTAACCAATGATTTTTCA
SMS_3_5                   TTCAACAACGCTTCCGGGAAACTGCTGGCGTTAGATAGCCAGTTAACCAATGATTTTTCA
RM12579                   TTCAACAACGCTTCCGGGAAACTGCTGGCGTTAGATAGCCAGTTAACCAATGATTTTTCA
CB9615                    TTCAACAACGCTTCCGGGAAACTGCTGGCGTTAGATAGCCAGTTAACCAATGATTTTTCA
Xuzhou21                  TTCAACAACGCTTCCGGGAAACTGCTGGCGTTAGATAGCCAGTTAACCAATGATTTTTCA
TW14359                   TTCAACAACGCTTCCGGGAAACTGCTGGCGTTAGATAGCCAGTTAACCAATGATTTTTCA
EC4115                    TTCAACAACGCTTCCGGGAAACTGCTGGCGTTAGATAGCCAGTTAACCAATGATTTTTCA
EDL933                    TTCAACAACGCTTCCGGGAAACTGCTGGCGTTAGATAGCCAGTTAACCAATGATTTTTCA
Sakaix28                  TTCAACAACGCTTCCGGGAAACTGCTGGCGTTAGATAGCCAGTTAACCAATGATTTTTCA
                          ***************** ******************************************

Prim.cons.                TTCAACAACGCTTCCGGGAAACTGCTGGCGTTAGATAGCCAGTTAACCAATGATTTTTCA

                                    790       800       810       820       830       840
                                      |         |         |         |         |         |
MG1655                    GAAAAAAG--CAGCTATTTCCAGTCACAGGTAGATAAAATCAGGAAGGAAGCATATGCCG
MDS42x1                   GAAAAAAG--CAGCTATTTCCAGTCACAGGTAGATAAAATCAGGAAGGAAGCATATGCCG
W3110x2                   GAAAAAAG--CAGCTATTTCCAGTCACAGGTAGATAAAATCAGGAAGGAAGCATATGCCG
DH1NC_0176381             GAAAAAAG--CAGCTATTTCCAGTCACAGGTAGATAAAATCAGGAAGGAAGCATATGCCG
ETECxx4                   GAAAAAAG--CAGCTATTTCCAGTCACAGGTAGATAAAATCAGGAAGGAAGCATATGCCG
P12bxx5                   GAAAAAAG--CAGCTATTTCCAGTCACAGGTAGATAAAATCAGGAAGGAAGCATATGCCG
BW2952                    GAAAAAAG--CAGCTATTTCCAGTCACAGGTAGATAAAATCAGGAAGGAAGCATATGCCG
DH10Bx7                   GAAAAAAG--CAGCTATTTCCAGTCACAGGTAGATAAAATCAGGAAGGAAGCATATGCCG
C321deltaA                GAAAAAAG--CAGCTATTTCCAGTCACAGGTAGATAAAATCAGGAAGGAAGCATATGCCG
Wxxxxx20                  GAAAAAAG--CAGCT-TTTCCAGTCACAGGTAGATAAAATCAGGAAGGAAGCGTATGCCG
KO11FL                    GAAAAAAG--CAGCT-TTTCCAGTCACAGGTAGATAAAATCAGGAAGGAAGCGTATGCCG
LY180x19                  GAAAAAAG--CAGCT-TTTCCAGTCACAGGTAGATAAAATCAGGAAGGAAGCGTATGCCG
E24377A                   GAAAAAAG--CAGCT-TTTCCAGTCACAGGTAGATAAAATCAGGAAGGAAGCGTATGCCG
SE11xx14                  GAAAAAAG--CAGCT-TTTCCAGTCACAGGTAGATAAAATCAGGAAGGAAGCGTATGCCG
2009EL_2050               GAAAAAAG--CAGCT-TTTCCAGTCACAGGTAGATAAAATCAGGAAGGAAGCGTATGCCG
2011C_3493                GAAAAAAG--CAGCT-TTTCCAGTCACAGGTAGATAAAATCAGGAAGGAAGCGTATGCCG
55989x18                  GAAAAAAG--CAGCT-TTTCCAGTCACAGGTAGATAAAATCAGGAAGGAAGCGTATGCCG
IAI1xx13                  GAAAAAAG--CAGCT-TTTCCAGTCACAGGTAGATAAAATCAGGAAGGAAGCGTATGCCG
O103H2                    GAAAAAAG--CAGCT-TTTCCAGTCACAGGTAGATAAAATCAGGAAGGAAGCGTATGCCG
APECxx11                  GAAAAAAG--CAGCT-TTTCCAGTCACAGGTAGATAAAATCAGGAAGGAAGCGTATGCCG
UMNK88                    GAAAAAAAA-CAGCTATTTCCAGTCACAGGTAGATAAAATCAGGAAGGAAGCGTATGCCG
```

```
HSxxxx39               GAAAAAAAAACAGCTATTTCCAGTCACAGGTAGATAAAATCAGGAAGGAAGCGTATGCCG
BL21DE3                GAAAAAAAA-CAGCTATTTCCAGTCACAGGTAGATAAAATCAGGAAGGAAGCGTATGCCG
REL606                 GAAAAAAAA-CAGCTATTTCCAGTCACAGGTAGATAAAATCAGGAAGGAAGCGTATGCCG
BL21_GoldDE3pLysS      GAAAAAAAA-CAGCTATTTCCAGTCACAGGTAGATAAAATCAGGAAGGAAGCGTATGCCG
ATCCxx37               GAAAAAAAA-CAGCTATTTCCAGTCACAGGTAGATAAAATCAGGAAGGAAGCGTATGCCG
O111H_                 GAAAAAAG--CAGCTATTTCCAGTCACAGGTAGATAAAATCAGGAAGGAAGCGTATGCCG
O26H11                 GAAAAAAG--CAGCTATTTCCAGTCACAGGTAGATAAAATCAGGAAGGAAGCGTATGCCG
UMN026                 GAAAAAAG--CAGCTATTTCCAGTCACAGGTAGATAAAATCAGGAAGGAAGCGTATGCCG
042NC_0176261          GAAAAAAG--CAGCTATTTCCAGTCACAGGTAGATAAAATCAGGAAGGAAGCGTATGCCG
IAI39x23               GAAAAAAG--CAGCTATTTCCAGTCACAGGTAGATAAAATCAGGAAGGAAGCGTATGCCG
O7K1xx29               GAAAAAAG--CAGCTATTTCCAGTCACAGGTAGATAAAATCAGGAAGGAAGCGTATGCCG
SMS_3_5                GAAAAAAG--CAGCTATTTCCAGTCACAGGTAGATAAAATCAGGAAGGAAGCGTATGCCG
RM12579                GAAAAAAG--CAGCTATTTCCAGTCACAGGTAGATAAAATCAGGAAGGAAGCGTATGCCG
CB9615                 GAAAAAAG--CAGCTATTTCCAGTCACAGGTAGATAAAATCAGGAAGGAAGCGTATGCCG
Xuzhou21               GAAAAAAG--CAGCTATTTCCAGTCACAGGTAGATAAAATCAGGAAGGAAGCGTATGCCG
TW14359                GAAAAAAG--CAGCTATTTCCAGTCACAGGTAGATAAAATCAGGAAGGAAGCGTATGCCG
EC4115                 GAAAAAAG--CAGCTATTTCCAGTCACAGGTAGATAAAATCAGGAAGGAAGCGTATGCCG
EDL933                 GAAAAAAG--CAGCTATTTCCAGTCACAGGTAGATAAAATCAGGAAGGAAGCGTATGCCG
Sakaix28               GAAAAAAG--CAGCTATTTCCAGTCACAGGTAGATAAAATCAGGAAGGAAGCGTATGCCG
                       *******   ***** ************************************ *******
Prim.cons.             GAAAAAAGAACAGCTATTTCCAGTCACAGGTAGATAAAATCAGGAAGGAAGCGTATGCCG

                              850       860       870       880       890       900
                                |         |         |         |         |         |
MG1655                 GTGCCGCAGCCGGTGTCGTCGCCGGTCCATTTGGATTAATCATTTCCTATTCTATTGCTG
MDS42x1                GTGCCGCAGCCGGTGTCGTCGCCGGTCCATTTGGATTAATCATTTCCTATTCTATTGCTG
W3110x2                GTGCCGCAGCCGGTGTCGTCGCCGGTCCATTTGGATTAATCATTTCCTATTCTATTGCTG
DH1NC_0176381          GTGCCGCAGCCGGTGTCGTCGCCGGTCCATTTGGATTAATCATTTCCTATTCTATTGCTG
ETECxx4                GTGCCGCAGCCGGTGTCGTCGCCGGTCCATTTGGATTAATCATTTCCTATTCTATTGCTG
P12bxx5                GTGCCGCAGCCGGTGTCGTCGCCGGTCCATTTGGATTAATCATTTCCTATTCTATTGCTG
BW2952                 GTGCCGCAGCCGGTGTCGTCGCCGGTCCATTTGGATTAATCATTTCCTATTCTATTGCTG
DH10Bx7                GTGCCGCAGCCGGTGTCGTCGCCGGTCCATTTGGATTAATCATTTCCTATTCTATTGCTG
C321deltaA             GTGCCGCAGCCGGTGTCGTCGCCGGTCCATTTGGATTAATCATTTCCTATTCTATTGCTG
Wxxxxx20               GTGCCGCAGCCGGTGTCGTCGCCGGTCCATTTGGATTAATCATTTCCTATTCTATTGCTG
KO11FL                 GTGCCGCAGCCGGTGTCGTCGCCGGTCCATTTGGATTAATCATTTCCTATTCTATTGCTG
LY180x19               GTGCCGCAGCCGGTGTCGTCGCCGGTCCATTTGGATTAATCATTTCCTATTCTATTGCTG
E24377A                GTGCCGCAGCCGGTGTCGTCGCCGGTCCATTTGGATTAATCATTTCCTATTCTATTGCTG
SE11xx14               GTGCCGCAGCCGGTGTCGTCGCCGGTCCATTTGGATTAATCATTTCCTATTCTATTGCTG
2009EL_2050            GTGCCGCAGCCGGTGTCGTCGCCGGTCCATTTGGATTAATCATTTCCTATTCTATTGCTG
2011C_3493             GTGCCGCAGCCGGTGTCGTCGCCGGTCCATTTGGATTAATCATTTCCTATTCTATTGCTG
55989x18               GTGCCGCAGCCGGTGTCGTCGCCGGTCCATTTGGATTAATCATTTCCTATTCTATTGCTG
IAI1xx13               GTGCCGCAGCCGGTGTCGTCGCCGGTCCATTTGGATTAATCATTTCCTATTCTATTGCTG
O103H2                 GTGCCGCAGCCGGTGTCGTCGCCGGTCCATTTGGATTAATCATTTCCTATTCTATTGCTG
APECxx11               GTGCCGCAGCCGGTGTCGTCGCCGGTCCATTTGGATTAATCATTTCCTATTCTATTGCTG
UMNK88                 GT------------GTCGTCGCCGGTCCATTTGGATTAATCATTTCCTATTCTATTGCTG
HSxxxx39               GT------------GTCGTCGCCGGTCCATTTGGATTAATCATTTCCTATTCTATTGCTG
BL21DE3                GT------------GTCGTCGCCGGTCCATTTGGATTAATCATTTCCTATTCTATTGCTG
REL606                 GT------------GTCGTCGCCGGTCCATTTGGATTAATCATTTCCTATTCTATTGCTG
BL21_GoldDE3pLysS      GT------------GTCGTCGCCGGTCCATTTGGATTAATCATTTCCTATTCTATTGCTG
ATCCxx37               GT------------GTCGTCGCCGGTCCATTTGGATTAATCATTTCCTATTCTATTGCTG
O111H_                 GTGCCGCAGCCGGTGTCGTCGCCGGTCCATTTGGATTAATCATTTCCTATTCTATTGCTG
O26H11                 GTGCCGCAGCCGGTGTCGTCGCCGGTCCATTTGGATTAATCATTTCCTATTCTATTGCTG
UMN026                 GTGCCGCAGCCGGTGTCGTCGCCGGTCCATTTGGATTAATCATTTCCTATTCTATTGCTG
042NC_0176261          GTGCCGCAGCCGGTGTCGTCGCCGGTCCATTTGGATTAATCATTTCCTATTCTATTGCTG
IAI39x23               GTGCCGCAGCCGGTGTCGTCGCCGGTCCATTTGGTTTAATCATTTCCTATTCTATTGCTG
O7K1xx29               GTGCCGCAGCCGGTGTCGTCGCCGGTCCATTTGGTTTAATCATTTCCTATTCTATTGCTG
SMS_3_5                GTGCCGCAGCCGGTGTCGTCGCCGGTCCATTTGGTTTAATCATTTCCTATTCTATTGCTG
RM12579                GTGCCGCAGCCGGTGTCGTCGCCGGTCCATTTGGTTTAATCATTTCCTATTCTATTGCTG
CB9615                 GTGCCGCAGCCGGTGTCGTCGCCGGTCCATTTGGTTTAATCATTTCCTATTCTATTGCTG
Xuzhou21               GTGCCGCAGCCGGTGTCGTCGCCGGTCCATTTGGTTTAATCATTTCCTATTCTATTGCTG
TW14359                GTGCCGCAGCCGGTGTCGTCGCCGGTCCATTTGGTTTAATCATTTCCTATTCTATTGCTG
EC4115                 GTGCCGCAGCCGGTGTCGTCGCCGGTCCATTTGGTTTAATCATTTCCTATTCTATTGCTG
EDL933                 GTGCCGCAGCCGGTGTCGTCGCCGGTCCATTTGGTTTAATCATTTCCTATTCTATTGCTG
Sakaix28               GTGCCGCAGCCGGTGTCGTCGCCGGTCCATTTGGTTTAATCATTTCCTATTCTATTGCTG
                       **            ******************** *************************
Prim.cons.             GTGCCGCAGCCGGTGTCGTCGCCGGTCCATTTGGATTAATCATTTCCTATTCTATTGCTG
```

```
                           910       920       930       940       950       960
                             |         |         |         |         |         |
MG1655              CGGGCGTAGTTGAAGGAAAACTGATTCCAGAATTGAAGAACAAGTTAAAATCTGTGCAGA
MDS42x1             CGGGCGTAGTTGAAGGAAAACTGATTCCAGAATTGAAGAACAAGTTAAAATCTGTGCAGA
W3110x2             CGGGCGTAGTTGAAGGAAAACTGATTCCAGAATTGAAGAACAAGTTAAAATCTGTGCAGA
DH1NC_0176381       CGGGCGTAGTTGAAGGAAAACTGATTCCAGAATTGAAGAACAAGTTAAAATCTGTGCAGA
ETECxx4             CGGGCGTAGTTGAAGGAAAACTGATTCCAGAATTGAAGAACAAGTTAAAATCTGTGCAGA
P12bxx5             CGGGCGTAGTTGAAGGAAAACTGATTCCAGAATTGAAGAACAAGTTAAAATCTGTGCAGA
BW2952              CGGGCGTAGTTGAAGGAAAACTGATTCCAGAATTGAAGAACAAGTTAAAATCTGTGCAGA
DH10Bx7             CGGGCGTAGTTGAAGGAAAACTGATTCCAGAATTGAAGAACAAGTTAAAATCTGTGCAGA
C321deltaA          CGGGCGTAGTTGAAGGAAAACTGATTCCAGAATTGAAGAACAAGTTAAAATCTGTGCAGA
Wxxxxx20            CGGGCGTAGTTGAAGGGAAACTGATTCCAGAATTGAAGAACAAGTTAAAATCTGTGCAGA
KO11FL              CGGGCGTAGTTGAAGGGAAACTGATTCCAGAATTGAAGAACAAGTTAAAATCTGTGCAGA
LY180x19            CGGGCGTAGTTGAAGGGAAACTGATTCCAGAATTGAAGAACAAGTTAAAATCTGTGCAGA
E24377A             CGGGCGTAGTTGAAGGGAAACTGATTCCAGAATTGAAGAACAAGTTAAAATCTGTGCAGA
SE11xx14            CGGGCGTAGTTGAAGGGAAACTGATTCCAGAATTGAAGAACAAGTTAAAATCTGTGCAGA
2009EL_2050         CGGGCGTAGTTGAAGGGAAACTGATTCCAGAATTGAAGAACAAGTTAAAATCTGTGCAGA
2011C_3493          CGGGCGTAGTTGAAGGGAAACTGATTCCAGAATTGAAGAACAAGTTAAAATCTGTGCAGA
55989x18            CGGGCGTAGTTGAAGGGAAACTGATTCCAGAATTGAAGAACAAGTTAAAATCTGTGCAGA
IAI1xx13            CGGGCGTAGTTGAAGGGAAACTGATTCCAGAATTGAAGAACAAGTTAAAATCTGTGCAGA
O103H2              CGGGCGTAGTTGAAGGGAAACTGATTCCAGAATTGAAGAACAAGTTAAAATCTGTGCAGA
APECxx11            CGGGCGTAGTTGAAGGGAAACTGATTCCAGAATTGAAGAACAAGTTAAAATCTGTGCAGA
UMNK88              CGGGCGTAGTTGAAGGGAAACTGATTCCAGAATTGAAGAACAAGTTAAAATCTGTGCAGA
HSxxxx39            CGGGCGTAGTTGAAGGGAAACTGATTCCAGAATTGAAGAACAAGTTAAAATCTGTGCAGA
BL21DE3             CGGGCGTAGTTGAAGGGAAACTGATTCCAGAATTGAAGAACAAGTTAAAATCTGTGCAGA
REL606              CGGGCGTAGTTGAAGGGAAACTGATTCCAGAATTGAAGAACAAGTTAAAATCTGTGCAGA
BL21_GoldDE3pLysS   CGGGCGTAGTTGAAGGGAAACTGATTCCAGAATTGAAGAACAAGTTAAAATCTGTGCAGA
ATCCxx37            CGGGCGTAGTTGAAGGGAAACTGATTCCAGAATTGAAGAACAAGTTAAAATCTGTGCAGA
O111H_              CGGGCGTAGTTGAAGGGAAACTGATTCCAGAATTGAAGAACAAGTTAAAATCTGTGCAGA
O26H11              CGGGCGTAGTTGAAGGGAAACTGATTCCAGAATTGAAGAACAAGTTAAAATCTGTGCAGA
UMN026              CGGGCGTAGTTGAAGGGAAACTGATTCCAGAATTGAAGAACAAGTTAAAATCTGTGCAGA
042NC_0176261       CGGGCGTAGTTGAAGGGAAACTGATTCCAGAATTGAAGAACAAGTTAAAGTCTGTGCAGA
IAI39x23            CGGGCGTAGTTGAAGGGAAACTGATTCCAGAATTGAAGAACAAGTTAAAATCTGTGCAGA
O7K1xx29            CGGGCGTAGTTGAAGGGAAACTGATTCCAGAATTGAAGAACAAGTTAAAATCTGTGCAGA
SMS_3_5             CGGGCGTAGTTGAAGGGAAACTGATTCCAGAATTGAAGAACAAGTTAAAATCTGTGCAGA
RM12579             CGGGCGTAGTTGAAGGGAAACTGATTCCAGAATTGAAGAACAAGTTAAAATCTGTGCAGA
CB9615              CGGGCGTAGTTGAAGGGAAACTGATTCCAGAATTGAAGAACAAGTTAAAATCTGTGCAGA
Xuzhou21            CGGGCGTAGTTGAAGGGAAACTGATTCCAGAATTGAAGAACAAGTTAAAATCTGTGCAGA
TW14359             CGGGCGTAGTTGAAGGGAAACTGATTCCAGAATTGAAGAACAAGTTAAAATCTGTGCAGA
EC4115              CGGGCGTAGTTGAAGGGAAACTGATTCCAGAATTGAAGAACAAGTTAAAATCTGTGCAGA
EDL933              CGGGCGTAGTTGAAGGGAAACTGATTCCAGAATTGAAGAACAAGTTAAAATCTGTGCAGA
Sakaix28            CGGGCGTAGTTGAAGGGAAACTGATTCCAGAATTGAAGAACAAGTTAAAATCTGTGCAGA
                    **************** ******************************** **********

Prim.cons.          CGGGCGTAGTTGAAGGGAAACTGATTCCAGAATTGAAGAACAAGTTAAAATCTGTGCAGA

                           970       980       990      1000      1010      1020
                             |         |         |         |         |         |
MG1655              ATTTCTTTACCACCCTGTCTAACACGGTTAAACAAGCGAATAAAGATATCGATGCCGCCA
MDS42x1             ATTTCTTTACCACCCTGTCTAACACGGTTAAACAAGCGAATAAAGATATCGATGCCGCCA
W3110x2             ATTTCTTTACCACCCTGTCTAACACGGTTAAACAAGCGAATAAAGATATCGATGCCGCCA
DH1NC_0176381       ATTTCTTTACCACCCTGTCTAACACGGTTAAACAAGCGAATAAAGATATCGATGCCGCCA
ETECxx4             ATTTCTTTACCACCCTGTCTAACACGGTTAAACAAGCGAATAAAGATATCGATGCCGCCA
P12bxx5             ATTTCTTTACCACCCTGTCTAACACGGTTAAACAAGCGAATAAAGATATCGATGCCGCCA
BW2952              ATTTCTTTACCACCCTGTCTAACACGGTTAAACAAGCGAATAAAGATATCGATGCCGCCA
DH10Bx7             ATTTCTTTACCACCCTGTCTAACACGGTTAAACAAGCGAATAAAGATATCGATGCCGCCA
C321deltaA          ATTTCTTTACCACCCTGTCTAACACGGTTAAACAAGCGAATAAAGATATCGATGCCGCCA
Wxxxxx20            ATTTCTTTACCACCCTGTCTAACACGGTTAAACAAGCGAATAAAGATATCGATGCCGCCA
KO11FL              ATTTCTTTACCACCCTGTCTAACACGGTTAAACAAGCGAATAAAGATATCGATGCCGCCA
LY180x19            ATTTCTTTACCACCCTGTCTAACACGGTTAAACAAGCGAATAAAGATATCGATGCCGCCA
E24377A             ATTTCTTTACCACCCTGTCTAACACGGTTAAACAAGCGAATAAAGATATCGATGCCGCCA
SE11xx14            ATTTCTTTACCACCCTGTCTAACACGGTTAAACAAGCGAATAAAGATATCGATGCCGCCA
2009EL_2050         ATTTCTTTACCACCCTGTCTAACACGGTTAAACAAGCGAATAAAGATATCGATGCCGCCA
2011C_3493          ATTTCTTTACCACCCTGTCTAACACGGTTAAACAAGCGAATAAAGATATCGATGCCGCCA
55989x18            ATTTCTTTACCACCCTGTCTAACACGGTTAAACAAGCGAATAAAGATATCGATGCCGCCA
IAI1xx13            ATTTCTTTACCACCCTGTCTAACACGGTTAAACAAGCGAATAAAGATATCGATGCCGCCA
O103H2              ATTTCTTTACCACCCTGTCTAACACGGTTAAACAAGCGAATAAAGATATCGATGCCGCCA
APECxx11            ATTTCTTTACCACCCTGTCTAACACGGTTAAACAAGCGAATAAAGATATCGATGCCGCCA
UMNK88              ATTTCTTTACCACCCTGTCAAACACGGTTAAACAAGCGAATAAAGATATCGATGCCGCCA
```

```
HSxxxx39            ATTTCTTTACCACCCTGTCAAACACGGTTAAACAAGCGAATAAAGATATCGATGCCGCCA
BL21DE3             ATTTCTTTACCACCCTGTCAAACACGGTTAAACAAGCGAATAAAGATATCGATGCCGCCA
REL606              ATTTCTTTACCACCCTGTCAAACACGGTTAAACAAGCGAATAAAGATATCGATGCCGCCA
BL21_GoldDE3pLysS   ATTTCTTTACCACCCTGTCAAACACGGTTAAACAAGCGAATAAAGATATCGATGCCGCCA
ATCCxx37            ATTTCTTTACCACCCTGTCAAACACGGTTAAACAAGCGAATAAAGATATCGATGCCGCCA
O111H_              ATTTCTTTACCACCCTGTCTAACACGGTTAAACAAGCGAATAAAGATATCGATGCCGCCA
O26H11              ATTTCTTTACCACCCTGTCTAACACGGTTAAACAAGCGAATAAAGATATCGATGCCGCCA
UMN026              GTTTCTTTACCACCCTGTCTAACACGGTTAAACAAGCGAATAAAGATATCGATGCCGCCA
042NC_0176261       GTTTCTTTACCACCCTGTCTAACACGGTTAAACAAGCGAATAAAGATATCGATGCCGCCA
IAI39x23            GTTTCTTTACCACCCTGTCTAACACGGTTAAACAAGCGAATAAAGATATCGATGCCGCCA
O7K1xx29            GTTTCTTTACCACCCTGTCTAACACGGTTAAACAAGCGAATAAAGATATCGATGCCGCCA
SMS_3_5             GTTTCTTTACCACCCTGTCTAACACGGTTAAACAAGCGAATAAAGATATCGATGCCGCCA
RM12579             GTTTCTTTACCACCCTGTCTAACACGGTTAAACAAGCGAATAAAGATATCGATGCCGCCA
CB9615              GTTTCTTTACCACCCTGTCTAACACGGTTAAACAAGCGAATAAAGATATCGATGCCGCCA
Xuzhou21            GTTTCTTTACCACCCTGTCTAACACGGTTAAACAAGCGAATAAAGATATCGATGCCGCCA
TW14359             GTTTCTTTACCACCCTGTCTAACACGGTTAAACAAGCGAATAAAGATATCGATGCCGCCA
EC4115              GTTTCTTTACCACCCTGTCTAACACGGTTAAACAAGCGAATAAAGATATCGATGCCGCCA
EDL933              GTTTCTTTACCACCCTGTCTAACACGGTTAAACAAGCGAATAAAGATATCGATGCCGCCA
Sakaix28            GTTTCTTTACCACCCTGTCTAACACGGTTAAACAAGCGAATAAAGATATCGATGCCGCCA
                     ****************** ****************************************
Prim.cons.          ATTTCTTTACCACCCTGTCTAACACGGTTAAACAAGCGAATAAAGATATCGATGCCGCCA

                          1030      1040      1050      1060      1070      1080
                             |         |         |         |         |         |
MG1655              AATTGAAATTAACCACCGAAATAGCCGCCATCGGTGAGATAAAAACGGAAACTGAAACAA
MDS42x1             AATTGAAATTAACCACCGAAATAGCCGCCATCGGTGAGATAAAAACGGAAACTGAAACAA
W3110x2             AATTGAAATTAACCACCGAAATAGCCGCCATCGGTGAGATAAAAACGGAAACTGAAACAA
DH1NC_0176381       AATTGAAATTAACCACCGAAATAGCCGCCATCGGTGAGATAAAAACGGAAACTGAAACAA
ETECxx4             AATTGAAATTAACCACCGAAATAGCCGCCATCGGTGAGATAAAAACGGAAACTGAAACAA
P12bxx5             AATTGAAATTAACCACCGAAATAGCCGCCATCGGTGAGATAAAAACGGAAACTGAAACAA
BW2952              AATTGAAATTAACCACCGAAATAGCCGCCATCGGTGAGATAAAAACGGAAACTGAAACAA
DH10Bx7             AATTGAAATTAACCACCGAAATAGCCGCCATCGGTGAGATAAAAACGGAAACTGAAACAA
C321deltaA          AATTGAAATTAACCACCGAAATAGCCGCCATCGGTGAGATAAAAACGGAAACTGAAACAA
Wxxxxx20            AATTGAAATTAACCACCGAAATAGCCGCCATCGGGGAGATAAAAACGGAAACTGAAACAA
KO11FL              AATTGAAATTAACCACCGAAATAGCCGCCATCGGGGAGATAAAAACGGAAACTGAAACAA
LY180x19            AATTGAAATTAACCACCGAAATAGCCGCCATCGGGGAGATAAAAACGGAAACTGAAACAA
E24377A             AATTGAAATTAACCACCGAAATAGCCGCCATCGGGGAGATAAAAACGGAAACTGAAACAA
SE11xx14            AATTGAAATTAACCACCGAAATAGCCGCCATCGGGGAGATAAAAACGGAAACTGAAACAA
2009EL_2050         AATTGAAATTAACCACCGAAATAGCCGCCATCGGGGAGATAAAAACGGAAACTGAAACAA
2011C_3493          AATTGAAATTAACCACCGAAATAGCCGCCATCGGGGAGATAAAAACGGAAACTGAAACAA
55989x18            AATTGAAATTAACCACCGAAATAGCCGCCATCGGGGAGATAAAAACGGAAACTGAAACAA
IAI1xx13            AATTGAAATTAACCACCGAAATAGCCGCCATCGGGGAGATAAAAACGGAAACTGAAACAA
O103H2              AATTGAAATTAACCACCGAAATAGCCGCCATCGGGGAGATAAAAACGGAAACTGAAACAA
APECxx11            AATTGAAATTAACCACCGAAATAGCCGCCATCGGGGAGATAAAAACGGAAACTGAAACAA
UMNK88              AATTGAAATTAACCACCGAAATAGTCGCCATCGGGGAGATAAAAACGGAAACTGAAACAA
HSxxxx39            AATTGAAATTAACCACCGAAATAGTCGCCATCGGGGAGATAAAAACGGAAACTGAAACAA
BL21DE3             AATTGAAATTAACCACCGAAATAGTCGCCATCGGGGAGATAAAAACGGAAACTGAAACAA
REL606              AATTGAAATTAACCACCGAAATAGTCGCCATCGGGGAGATAAAAACGGAAACTGAAACAA
BL21_GoldDE3pLysS   AATTGAAATTAACCACCGAAATAGTCGCCATCGGGGAGATAAAAACGGAAACTGAAACAA
ATCCxx37            AATTGAAATTAACCACCGAAATAGTCGCCATCGGGGAGATAAAAACGGAAACTGAAACAA
O111H_              AATTGAAATTAACCACCGAAATAGCCGCCATCGGGGAGATAAAAACGGAAACTGAAACAA
O26H11              AATTGAAATTAACCACCGAAATAGCCGCCATCGGGGAGATAAAAACGGAAACTGAAACAA
UMN026              AATTGAAATTAACCACCGAAATAGCCGCCATCGGGGAGATAAAAACGGAAACTGAAACAA
042NC_0176261       AATTGAAATTAACCACCGAAATAGCCGCCATCGGGGAGATAAAAACGGAAACTGAAACAA
IAI39x23            AATTGAAATTAACCACCGAAATAGCCGCCATCGGGGAGATAAAAACGGAAACTGAAACAA
O7K1xx29            AATTGAAATTAACCACCGAAATAGCCGCCATCGGGGAGATAAAAACGGAAACTGAAACAA
SMS_3_5             AATTGAAATTAACCACCGAAATAGCCGCCATCGGGGAGATAAAAACGGAAACTGAAACAA
RM12579             AATTGAAATTAACCACCGAAATAGCCGCCATCGGGGAGATAAAAACGGAAACTGAAACAA
CB9615              AATTGAAATTAACCACCGAAATAGCCGCCATCGGGGAGATAAAAACGGAAACTGAAACAA
Xuzhou21            AATTGAAATTAACCACCGAAATAGCCGCCATCGGGGAGATAAAAACGGAAACTGAAACAA
TW14359             AATTGAAATTAACCACCGAAATAGCCGCCATCGGGGAGATAAAAACGGAAACTGAAACAA
EC4115              AATTGAAATTAACCACCGAAATAGCCGCCATCGGGGAGATAAAAACGGAAACTGAAACAA
EDL933              AATTGAAATTAACCACCGAAATAGCCGCCATCGGGGAGATAAAAACGGAAACTGAAACAA
Sakaix28            AATTGAAATTAACCACCGAAATAGCCGCCATCGGGGAGATAAAAACGGAAACTGAAACAA
                    ************************ ********* *************************
Prim.cons.          AATTGAAATTAACCACCGAAATAGCCGCCATCGGGGAGATAAAAACGGAAACTGAAACAA
```

```
                            1090      1100      1110      1120      1130      1140
                               |         |         |         |         |         |
MG1655                CCAGATTCTACGTTGATTATGATGATTTAATGCTTTCTTTGCTAAAAGAAGCGGCCAAAA
MDS42x1               CCAGATTCTACGTTGATTATGATGATTTAATGCTTTCTTTGCTAAAAGAAGCGGCCAAAA
W3110x2               CCAGATTCTACGTTGATTATGATGATTTAATGCTTTCTTTGCTAAAAGAAGCGGCCAAAA
DH1NC_0176381         CCAGATTCTACGTTGATTATGATGATTTAATGCTTTCTTTGCTAAAAGAAGCGGCCAAAA
ETECxx4               CCAGATTCTACGTTGATTATGATGATTTAATGCTTTCTTTGCTAAAAGAAGCGGCCAAAA
P12bxx5               CCAGATTCTACGTTGATTATGATGATTTAATGCTTTCTTTGCTAAAAGAAGCGGCCAAAA
BW2952                CCAGATTCTACGTTGATTATGATGATTTAATGCTTTCTTTGCTAAAAGAAGCGGCCAAAA
DH10Bx7               CCAGATTCTACGTTGATTATGATGATTTAATGCTTTCTTTGCTAAAAGAAGCGGCCAAAA
C321deltaA            CCAGATTCTACGTTGATTATGATGATTTAATGCTTTCTTTGCTAAAAGAAGCGGCCAAAA
Wxxxxx20              CCAGATTCTATGTTGATTATGATGATTTAATGCTTTCTTTGCTAAAAGAAGCGGCAAAAA
KO11FL                CCAGATTCTATGTTGATTATGATGATTTAATGCTTTCTTTGCTAAAAGAAGCGGCAAAAA
LY180x19              CCAGATTCTATGTTGATTATGATGATTTAATGCTTTCTTTGCTAAAAGAAGCGGCAAAAA
E24377A               CCAGATTCTATGTTGATTATGATGATTTAATGCTTTCTTTGCTAAAAGAAGCGGCAAAAA
SE11xx14              CCAGATTCTATGTTGATTATGATGATTTAATGCTTTCTTTGCTAAAAGAAGCGGCAAAAA
2009EL_2050           CCAGATTCTATGTTGATTATGATGATTTAATGCTTTCTTTGCTAAAAGAAGCGGCAAAAA
2011C_3493            CCAGATTCTATGTTGATTATGATGATTTAATGCTTTCTTTGCTAAAAGAAGCGGCAAAAA
55989x18              CCAGATTCTATGTTGATTATGATGATTTAATGCTTTCTTTGCTAAAAGAAGCGGCAAAAA
IAI1xx13              CCAGATTCTATGTTGATTATGATGATTTAATGCTTTCTTTGCTAAAAGAAGCGGCAAAAA
O103H2                CCAGATTCTATGTTGATTATGATGATTTAATGCTTTCTTTGCTAAAAGAAGCGGCAAAAA
APECxx11              CCAGATTCTATGTTGATTATGATGATTTAATGCTTTCTTTGCTAAAAGAAGCGGCAAAAA
UMNK88                CCAGATTCTATGTTGATTATGATGATTTAATGCTTTCTTTGCTAAAAGAAGCGGCCAAAA
HSxxxx39              CCAGATTCTATGTTGATTATGATGATTTAATGCTTTCTTTGCTAAAAGAAGCGGCCAAAA
BL21DE3               CCAGATTCTATGTTGATTATGATGATTTAATGCTTTCTTTGCTAAAAGAAGCGGCCAAAA
REL606                CCAGATTCTATGTTGATTATGATGATTTAATGCTTTCTTTGCTAAAAGAAGCGGCCAAAA
BL21_GoldDE3pLysS     CCAGATTCTATGTTGATTATGATGATTTAATGCTTTCTTTGCTAAAAGAAGCGGCCAAAA
ATCCxx37              CCAGATTCTATGTTGATTATGATGATTTAATGCTTTCTTTGCTAAAAGAAGCGGCCAAAA
O111H_                CCAGATTCTATGTTGATTATGATGATTTAATGCTTTCTTTGCTAAAAGAAGCGGCAAAAA
O26H11                CCAGATTCTATGTTGATTATGATGATTTAATGCTTTCTTTGCTAAAAGAAGCGGCAAAAA
UMN026                CCAGATTCTACGTTGATTATGATGATTTAATGCTTTCTTTGCTAAAAGAAGCGGCCAAAA
O42NC_0176261         CCAGATTCTACGTTGATTATGATGATTTAATGCTTTCTTTGCTAAAAGAAGCGGCAAAAA
IAI39x23              CCAGATTCTATGTTGATTATGATGATTTAATGCTTTCTTTGCTAAAAGAAGCGGCCAAAA
O7K1xx29              CCAGATTCTATGTTGATTATGATGATTTAATGCTTTCTTTGCTAAAAGAAGCGGCCAAAA
SMS_3_5               CCAGATTCTACGTTGATTATGATGATTTAATGCTTTCTTTGCTAAAAGAAGCGGCAAAAA
RM12579               CCAGATTCTATGTTGATTATGATGATTTAATGCTTTCTTTGCTAAAAGAAGCGGCCAATA
CB9615                CCAGATTCTATGTTGATTATGATGATTTAATGCTTTCTTTGCTAAAAGAAGCGGCCAATA
Xuzhou21              CCAGATTCTATGTTGATTATGATGATTTAATGCTTTCTTTGCTAAAAGAAGCGGCCAATA
TW14359               CCAGATTCTATGTTGATTATGATGATTTAATGCTTTCTTTGCTAAAAGAAGCGGCCAATA
EC4115                CCAGATTCTATGTTGATTATGATGATTTAATGCTTTCTTTGCTAAAAGAAGCGGCCAATA
EDL933                CCAGATTCTATGTTGATTATGATGATTTAATGCTTTCTTTGCTAAAAGAAGCGGCCAATA
Sakaix28              CCAGATTCTATGTTGATTATGATGATTTAATGCTTTCTTTGCTAAAAGAAGCGGCCAATA
                      ********** ******************************************** ** *

Prim.cons.            CCAGATTCTATGTTGATTATGATGATTTAATGCTTTCTTTGCTAAAAGAAGCGGCCAAAA

                            1150      1160      1170      1180      1190      1200
                               |         |         |         |         |         |
MG1655                AAA-TGATTAACACCTGTAATGAGTATCAGAAAAGACACGGTAAAAAGACACTCTTTGAG
MDS42x1               AAA-TGATTAACACCTGTAATGAGTATCAGAAAAGACACGGTAAAAAGACACTCTTTGAG
W3110x2               AAA-TGATTAACACCTGTAATGAGTATCAGAAAAGACACGGTAAAAAGACACTCTTTGAG
DH1NC_0176381         AAA-TGATTAACACCTGTAATGAGTATCAGAAAAGACACGGTAAAAAGACACTCTTTGAG
ETECxx4               AAA-TGATTAACACCTGTAATGAGTATCAGAAAAGACACGGTAAAAAGACACTCTTTGAG
P12bxx5               AAA-TGATTAACACCTGTAATGAGTATCAGAAAAGACACGGTAAAAAGACACTCTTTGAG
BW2952                AAA-TGATTAACACCTGTAATGAGTATCAGAAAAGACACGGTAAAAAGACACTCTTTGAG
DH10Bx7               AAA-TGATTAACACCTGTAATGAGTATCAGAAAAGACACGGTAAAAAGACACTCTTTGAG
C321deltaA            AAA-TGATTAACACCTGTAATGAGTATCAGAAAAGACACGGTAAAAAGACACTCTTTGAG
Wxxxxx20              AAA-TGATTAACACCTGTAATGAGTATCAGAAAAGACACGGTAAAAAGACACTCTTTGAG
KO11FL                AAA-TGATTAACACCTGTAATGAGTATCAGAAAAGACACGGTAAAAAGACACTCTTTGAG
LY180x19              AAA-TGATTAACACCTGTAATGAGTATCAGAAAAGACACGGTAAAAAGACACTCTTTGAG
E24377A               AAA-TGATTAACACCTGTAATGAGTATCAGAAAAGACACGGTAAAAAGACACTCTTTGAG
SE11xx14              AAA-TGATTAACACCTGTAATGAGTATCAGAAAAGACACGGTAAAAAGACACTCTTTGAG
2009EL_2050           AAA-TGATTAACACCTGTAATGAGTATCAGAAAAGACACGGTAAAAAGACACTCTTTGAG
2011C_3493            AAA-TGATTAACACCTGTAATGAGTATCAGAAAAGACACGGTAAAAAGACACTCTTTGAG
55989x18              AAA-TGATTAACACCTGTAATGAGTATCAGAAAAGACACGGTAAAAAGACACTCTTTGAG
IAI1xx13              AAA-TGATTAACACCTGTAATGAGTATCAGAAAAGACACGGTAAAAAGACACTCTTTGAG
O103H2                AAA-TGATTAACACCTGTAATGAGTATCAGAAAAGACACGGTAAAAAGACACTCTTTGAG
APECxx11              AAA-TGATTAACACCTGTAATGAGTATCAGAAAAGACACGGTAAAAAGACACTCTTTGAG
UMNK88                AAA-TGATTAACACCTGTAATGAGTATCAGAAAAGACACGGTAAAAAGACACTCTTTGAG
```

```
HSxxxx39              AAA-TGATTAACACCTGTAATGAGTATCAGAAAAGACACGGTAAAAAGACACTCTTTGAG
BL21DE3               AAA-TGATTAACACCTGTAATGAGTATCAGAAAAGACACGGTAAAAAGACACTCTTTGAG
REL606                AAA-TGATTAACACCTGTAATGAGTATCAGAAAAGACACGGTAAAAAGACACTCTTTGAG
BL21_GoldDE3pLysS     AAA-TGATTAACACCTGTAATGAGTATCAGAAAAGACACGGTAAAAAGACACTCTTTGAG
ATCCxx37              AAA-TGATTAACACCTGTAATGAGTATCAGAAAAGACACGGTAAAAAGACACTCTTTGAG
O111H_                AAA-TGATTAACACCTGTAATGAGTATCAGAAAAGACACGGTAAAAAGACACTCTTTGAG
O26H11                AAA-TGATTAACACCTGTAATGAGTATCAGAAAAGACACGGTAAAAAGACACTCTTTGAG
UMN026                AAA-TGATTAACACCTGTAATGAGTATCAGAAAAGACACGGTAAGAAGACACTCTTTGAG
042NC_0176261         AAA-TGATTAACACCTGTAATGAGTATCAGAAAAGACACGGTAAGAAGACACTCTTTGAG
IAI39x23              AAA-TGATTAACACCTGTAATGAGTATCAGAAAAGACACGGTAAGAAGACACTCTTTGAG
O7K1xx29              AAAATGATTAACACCTGTAATGAGTATCAGAAAAGACACGGTAAGAAGACACTCTTTGAG
SMS_3_5               AAA-TGATTAACACCTGTAATGAGTATCAGAAAAGACACGGTAAGAAGACACTCTTTGAG
RM12579               AAA-TGATTAACACCTGTAATGAGTATCAGAAAAGACACGGTAAGAAGACACTCTTTGAG
CB9615                AAA-TGATTAACACCTGTAATGAGTATCAGAAAAGACACGGTAAGAAGACACTCTTTGAG
Xuzhou21              AAA-TGATTAACACCTGTAATGAGTATCAGAAAAGACACGGTAAGAAGACACTCTTTGAG
TW14359               AAA-TGATTAACACCTGTAATGAGTATCAGAAAAGACACGGTAAGAAGACACTCTTTGAG
EC4115                AAA-TGATTAACACCTGTAATGAGTATCAGAAAAGACACGGTAAGAAGACACTCTTTGAG
EDL933                AAA-TGATTAACACCTGTAATGAGTATCAGAAAAGACACGGTAAGAAGACACTCTTTGAG
Sakaix28              AAA-TGATTAACACCTGTAATGAGTATCAGAAAAGACACGGTAAGAAGACACTCTTTGAG
                      *** **************************************** ***************
Prim.cons.            AAAATGATTAACACCTGTAATGAGTATCAGAAAAGACACGGTAAAAAGACACTCTTTGAG

                            1210
                               |
MG1655                GTACCTGAAGTCTGA   .... 1230694
MDS42x1               GTACCTGAAGTCTGA
W3110x2               GTACCTGAAGTCTGA
DH1NC_0176381         GTACCTGAAGTCTGA
ETECxx4               GTACCTGAAGTCTGA
P12bxx5               GTACCTGAAGTCTGA
BW2952                GTACCTGAAGTCTGA
DH10Bx7               GTACCTGAAGTCTGA
C321deltaA            GTACCTGAAGTCTGA
Wxxxxx20              GTACCTGAAGTCTGA
KO11FL                GTACCTGAAGTCTGA
LY180x19              GTACCTGAAGTCTGA
E24377A               GTACCTGAAGTCTGA
SE11xx14              GTACCTGAAGTCTGA
2009EL_2050           GTACCTGAAGTCTGA
2011C_3493            GTACCTGAAGTCTGA
55989x18              GTACCTGAAGTCTGA
IAI1xx13              GTACCTGAAGTCTGA
O103H2                GTACCTGAAGTCTGA
APECxx11              GTACCTGAAGTCTGA
UMNK88                GTACCTGAAGTCTGA
HSxxxx39              GTACCTGAAGTCTGA
BL21DE3               GTACCTGAAGTCTGA
REL606                GTACCTGAAGTCTGA
BL21_GoldDE3pLysS     GTACCTGAAGTCTGA
ATCCxx37              GTACCTGAAGTCTGA
O111H_                GTACCTGAAGTCTGA
O26H11                GTACCTGAAGTCTGA
UMN026                GTACCTGAAGTCTGA
042NC_0176261         GTACCTGAAGTCTGA
IAI39x23              GTACCTGAAGTCTGA
O7K1xx29              GTACCTGAAGTCTGA
SMS_3_5               GTACCTGAAGTCTGA
RM12579               GTACCTGAAGTCTGA
CB9615                GTACCTGAAGTCTGA
Xuzhou21              GTACCTGAAGTCTGA
TW14359               GTACCTGAAGTCTGA
EC4115                GTACCTGAAGTCTGA
EDL933                GTACCTGAAGTCTGA
Sakaix28              GTACCTGAAGTCTGA
                      ***************
Prim.cons.            GTACCTGAAGTCTGA
```

Figure S1:

The open access soft ware programs
BLAST: (http://blast.ncbi.nlm.nih.gov/Blast.cgi) to search for sequence and strains with similarities to the *clyA*/*hlyE* gene, and
ClustalW: (http://pbil.ibcp.fr/htm/index.php) were used for alignment of the sequences with the *clyA* gene sequences of *E. coli* strain MG1655 (accession number of NC_000913.3) as the reference sequence. It includes the 1.2 kb region between nucleotide coordinates 1229483 and 1230694 and represents the entire *clyA* coding sequence and 300 base pairs upstream of the ATG start codon. The sequence of *clyA* was compared with the corresponding sequences of *clyA* in other *E. coli* strains (both IPEC and ExPEC).
Black nucleotide lettering and dashes indicates position where there are differences in the otherwise red letter compilation.
The translational start sequences (ATG and ribosome binding motif) are marked in green.
The transcriptional start sequences -35 and -10 motifs and the initiating nucleotide are marked in blue.
The binding site for the CRP/FNR regulators is marked in grey.
The binding sequences for SlyA are underlined.
