# Supplementary material for: Elevated recombinant clyA gene expression in the uropathogenic Escherichia coli strain 536, a clue to explain pathoadaptive mutations in a subset of extraintestinal E. coli strains
Source: BMC Microbiol. 2014 Sep 2;14:216. doi: 10.1186/s12866-014-0216-4 (PMC4164713; doi:10.1186/s12866-014-0216-4)
Supplement: Additional file 2: — Effect of Polymyxin B on the growth of E. coli K-12 derivatives MC4100 and MWK11 in batch cultures of medium AUM at 37°C. [file 12866_2014_216_MOESM2_ESM.pdf]

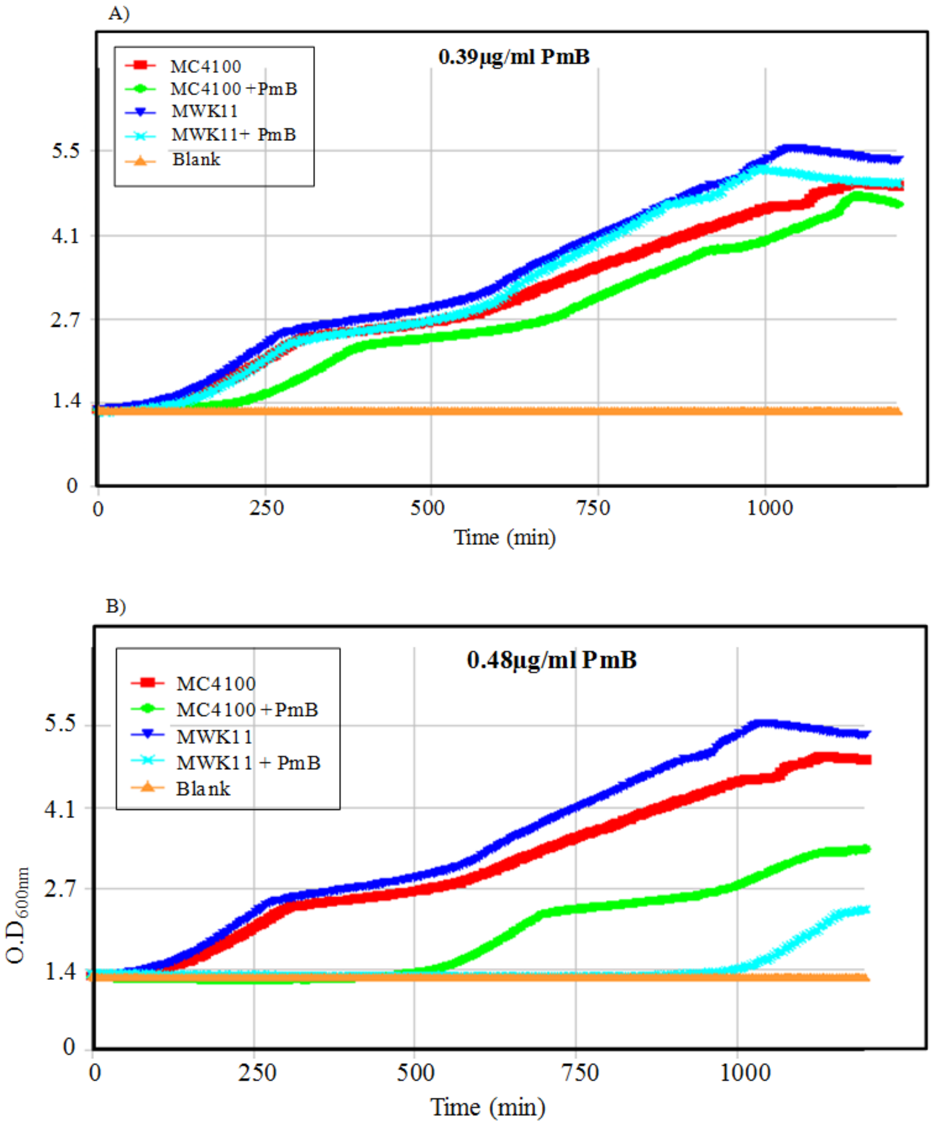

Figure S2:
Effect of Polymyxin B on the growth of *E. coli* K-12 derivatives MC4100 and MWK11 in batch cultures of medium AUM at 37°C.
